# Multi-Strategy Improved Connected Banking System Optimizer for Numerical Optimization and Real Problems

**DOI:** 10.3390/biomimetics11070487

**Published:** 2026-07-10

**Authors:** Song Liu, Xiaodan Tang, Chengpeng Li

**Affiliations:** 1 School of Economics, South-Central Minzu University, Wuhan 430073, China; 2 School of Financial, Southwestern University of Finance and Economics, Chengdu 611130, China; 3 Taizhou Institute of Zhejiang University, Taizhou 318000, China; 4 State Key Laboratory of Industrial Control Technology, College of Control Science and Engineering, Zhejiang University, Hangzhou 310027, China

**Keywords:** metaheuristic algorithms, swarm intelligence, 3D UAV path planning, connected banking system optimizer, numerical optimization

## Abstract

This paper proposes a Multi-Strategy Improved Connected Banking System Optimizer, named MICBSO, for numerical optimization and three-dimensional UAV path planning. MICBSO enhances the original CBSO through three coordinated strategies. First, a chaos–opposition learning initialization strategy is introduced to improve initial population quality and search coverage. Second, a Gaussian perturbation-based multi-elite guidance mechanism is designed to reduce dependence on a single best solution and strengthen the balance between exploration and exploitation. Third, a hybrid boundary control strategy combining reflective correction and random reinitialization is developed to improve solution feasibility and maintain population diversity. The proposed algorithm is evaluated on the CEC2017 benchmark suite and compared with 11 representative algorithms. Experimental results show that MICBSO achieves competitive convergence accuracy, stability, and robustness across different dimensional settings. In addition, MICBSO is applied to three-dimensional UAV path planning in four complex terrain scenarios. The results demonstrate that MICBSO can generate feasible and safe flight paths with lower comprehensive cost. Overall, the proposed method provides an effective optimization framework for both benchmark optimization and constrained UAV path planning tasks.

## 1. Introduction

In recent years, the rapid development of the low-altitude economy and the continuous optimization of airspace management policies have made low-altitude airspace an emerging field with substantial economic potential. The low-altitude economy covers logistics transportation [1], emergency response [2], precision agriculture [3], aerial monitoring [4], and various other application domains [5], all of which have significantly accelerated the advancement and widespread adoption of unmanned aerial vehicle (UAV) technologies. As a core carrier of low-altitude intelligent services, UAVs demonstrate remarkable advantages in autonomy, flexibility, and collaborative operation. With the growing scale and complexity of their applications, higher requirements are being placed on the underlying decision-making and optimization capabilities that support UAV operations. Among these capabilities, path planning remains one of the most critical components.

UAV path planning aims to generate a safe, feasible, and cost-efficient trajectory from a starting point to a target location within a complex three-dimensional environment [6]. This process must consider multiple constraints, including obstacle avoidance, no-fly zones, energy consumption, communication limits, and mission requirements. The inherently high dimensionality, nonlinearity, multimodality, and multi-constraint characteristics of UAV path planning make it a challenging global optimization problem. Traditional analytical or deterministic optimization approaches often fail to meet practical needs due to their strong dependence on problem models and limited adaptability to complex or dynamic environments. Consequently, heuristic and meta-heuristic algorithms have become mainstream solutions for UAV path planning due to their gradient-free nature and strong capability in navigating complex search spaces. For example, Wu et al. analyzed the significance of multi-UAV cooperative flight and proposed an optimization model for multi-UAV cooperative route planning [7]. They then developed a multi-UAV cooperative path planning algorithm based on co-evolutionary optimization. Experimental results demonstrate that the proposed algorithm effectively addresses the challenge of multi-UAV cooperative path planning in complex environments. Wang et al. developed a path planning model incorporating multiple constraints based on rugged mountainous terrain and dynamic obstacles [8]. They proposed an arithmetic optimization algorithm combining adaptive thermal conduction search, quadratic interpolation, and elite population genetic strategies (TQGAOA). Comparative experiments across six mountainous terrain scenarios with dynamic obstacles demonstrated that TQGAOA flexibly adapts to varying levels of complexity, consistently generating high-quality drone planning paths in a stable and efficient manner. Zhang et al. proposed a heuristic cross-search and rescue optimization algorithm by integrating a heuristic crossover strategy with the basic SAR [9]. They employed cubic B-spline interpolation to smooth the generated paths, effectively addressing the path planning challenge for unmanned aerial vehicles.

However, existing algorithms still face challenges such as insufficient convergence speed, premature convergence, and limited global search performance when confronted with increasingly complex UAV scenarios. Many researchers have made targeted improvements to algorithms for specific problems to achieve better solutions. For instance, Qin et al. proposed an enhanced Redtail Hawk algorithm based on multiple elite strategies and chaotic mapping to address task scheduling problems [10]. Combined with the proposed scheduling model, this approach optimizes task scheduling efficiency in cloud computing environments. The development and validation of the ERTH algorithm holds significant importance for advancing the application of intelligent optimization algorithms in cloud computing. Fu et al. proposed an accurate elimination and generation mechanism for point cloud registration and improved LSHADE-SPACMA by incorporating a modified semi-parametric adaptive strategy and a mutation strategy based on rank-based selection pressure [11]. Simulation analyses across 25 point cloud registration cases demonstrated excellent results. Zhou et al. integrated a sine-chaotic mapping strategy [12], a Levy flight strategy, and an adaptive weight variation operator to enhance the DBO, thereby establishing the SLPDBO-BP data asset valuation model. Experimental results demonstrate that the proposed SLPDBO-BP model exhibits excellent evaluation accuracy, outperforming existing state-of-the-art algorithms across all metrics. Xu et al. employed a reduced-order sliding mode observer to achieve real-time fault detection and isolation, proposing a fault-tolerant control framework suitable for low-altitude UAV swarms. Experimental results demonstrate that the fault-tolerant control system exhibits exceptional accuracy, stability, and computational efficiency across various fault scenarios, fully validating its feasibility for practical UAV swarm applications. This framework offers a reliable and scalable solution for ensuring the continuous operation of UAV swarms during mission-critical tasks [13].

The Connection bank system optimizer (CBSO) is a heuristic algorithm proposed by Nemati et al. in 2024 [14]. By modeling interactions such as resource flow, risk adjustment, and information transfer among banking entities, CBSO introduces a novel mechanism balancing exploration and exploitation, achieving outstanding results in truss size and layout optimization. As CBSO requires no additional parameter configuration, this algorithm holds broad application prospects as a robust, parameter-free optimization method.

Therefore, motivated by the aforementioned studies and aiming to effectively solve the three-dimensional drone path planning problem investigated in this paper, a multi-strategy improved connection bank system optimizer (MICBSO) is proposed. Although chaotic initialization, opposition-based learning, elite guidance, Gaussian perturbation, and boundary handling have been widely used in improved metaheuristic algorithms, the proposed MICBSO is not a simple combination of these existing operators. Its novelty lies in the stage-aware integration of these strategies into the search structure of CBSO. Specifically, the chaos–opposition learning strategy is embedded into the initialization stage to improve the distribution quality of banking entities; the Gaussian perturbation-based multi-elite guidance mechanism is designed to overcome the single-best-guided search tendency of CBSO; and the hybrid boundary control strategy adaptively combines reflective correction and random reinitialization to preserve feasible search directions while maintaining diversity. Compared with similar improved metaheuristic algorithms that introduce enhancement operators independently, MICBSO constructs a coordinated framework that jointly considers initialization quality, elite-guided search stability, boundary feasibility, and UAV path planning constraints.

The main contributions of this study are summarized as follows.

1. A multi-strategy improved Connected Banking System Optimizer, named MICBSO, is proposed for numerical optimization and three-dimensional UAV path planning in complex terrain. Different from existing improved swarm intelligence algorithms that simply combine general enhancement operators, MICBSO integrates the proposed strategies into the search structure of CBSO in a stage-aware manner, aiming to improve initialization quality, elite-guided search, and solution feasibility simultaneously.
2. A chaos–opposition learning-based initialization strategy is designed to improve the quality and diversity of the initial banking entities. Unlike conventional random initialization or single chaotic initialization, the proposed strategy combines Logistic chaotic mapping with opposition-based learning to generate candidate solutions from two complementary directions, thereby expanding the initial search coverage and providing a more reliable starting population for the subsequent CBSO updating process.
3. A Gaussian perturbation-based multi-elite guidance mechanism is developed to overcome the overdependence of CBSO on a single best solution. Instead of disturbing only the current global optimum, MICBSO uses the collective information of multiple elite individuals to construct a more stable guidance direction, while Gaussian perturbation is introduced to enhance local refinement and reduce the probability of premature convergence.
4. A hybrid boundary control strategy combining reflective correction and random reinitialization is proposed to handle boundary violations during the optimization process. Different from simple clipping or direct random resetting, the proposed mechanism treats minor and severe boundary violations differently: reflection is used to preserve useful search direction information, whereas random reinitialization is adopted to avoid repeated boundary oscillation and maintain population diversity.
5. The proposed MICBSO is systematically evaluated on 30 benchmark functions from the IEEE CEC2017 test suite and compared with 11 representative optimization algorithms. In addition, comprehensive statistical analyses are conducted to verify its optimization accuracy, stability, and robustness. Furthermore, MICBSO is applied to three-dimensional UAV path planning under four complex threat scenarios, and the simulation results further demonstrate its effectiveness, feasibility, and practical applicability in constrained path planning tasks.

The remainder of this paper is organized as follows. Section 2 briefly introduces the CBSO. Section 3 presents the three improved strategies proposed to address the UAV path planning problem. In Section 4, MICBSO is evaluated through numerical optimization experiments, and the corresponding results are analyzed in detail. Section 5 applies the proposed algorithm to UAV path planning in a realistic environment and provides a comprehensive discussion of its advantages and limitations. Finally, Section 6 concludes the paper and outlines directions for future research.

## 2. Connected Banking System Optimizer (CBSO)

Connected Banking System Optimizer (CBSO) is inspired by the complex interconnected structure of the global banking system: banks transact through multiple paths, constantly selecting the fastest and most reliable transfer route in a high-risk environment where system crashes are possible. The core idea is to treat interbank transaction paths as search agents, using transaction completion time as the optimization objective, and considering characteristics of the financial system such as the “influence of core banks,” the propagation of cascading failures, and rerouting after random crashes. This results in a three-stage metaheuristic optimization algorithm that simulates the dynamic decision-making process of the banking system. The specific mathematical model is shown below.

### 2.1. Algorithm Initialization

Similar to other metaheuristic algorithms, the initial position of the search agent in CBSO is randomly determined by the upper and lower bounds of the optimization problem. The search agent, also known as a candidate solution, is denoted as X and can be represented by Equation (1).

(1) $$X=\left[\begin{array}{c} X_{1}\\ \vdots \\ X_{i}\\ \vdots \\ X_{n} \end{array}\right]=\left[\begin{array}{ccccc} x_{1}^{1} & \ldots & x_{1}^{j} & \ldots & x_{1}^{d}\\ \vdots & \ddots & \vdots & \ddots & \vdots \\ x_{i}^{1} & \ldots & x_{i}^{j} & \ldots & x_{i}^{d}\\ \vdots & \ddots & \vdots & \ddots & \vdots \\ x_{n}^{1} & \ldots & x_{n}^{j} & \ldots & x_{n}^{d} \end{array}\right],\;\;\left\{\begin{array}{c} i=1,2,\ldots ,n.\\ j=1,2,\ldots ,d. \end{array}\right.$$

where n represents the number of candidate solutions, and d represents the dimension of the problem. $x_{i}^{j}$ represents the j-th decision variable at the i-th initial position, which can be calculated using Equation (2).

(2) $$x_{i}^{j}=x_{i,min}^{j}+r\cdot (x_{i,max}^{j}-x_{i,min}^{j}),\;\;\left\{\begin{array}{c} i=1,2,\ldots ,n.\\ j=1,2,\ldots ,d. \end{array}\right.$$

where $x_{i,max}^{j}$ and $x_{i,min}^{j}$ are the upper and lower bounds of the decision variables in the optimization problem, respectively. r represents a random number between 0 and 1. During the algorithm’s iteration process, the position of candidate solutions is updated through three stages defined by CBSO, as described below.

### 2.2. Phase 1

In the iteration process of the CBSO algorithm, the first 20% of the iteration process is defined as phase 1, and its position update method can be expressed as Equation (3).

(3) $$\vec{NewX_{i}}=\vec{X_{i}}+\vec{{RN}_{1}}⊗((r1\cdot \vec{BB}-\vec{X_{i}}))i=1,\ldots ,n$$

where $\vec{BB}$ denotes the current best solution found by the population at the current iteration, r1 represents a random number between 0 and 1, $\vec{NewX_{i}}$ represents the new search agent, $\vec{X_{i}}$ represents the current search agent, and $\vec{{RN}_{1}}$ represents a normally distributed random number vector.

### 2.3. Phase 2

In CBSO, the search process is divided according to the normalized iteration ratio $t/t_{max}$, where t is the current iteration number and $t_{max}$ is the preset maximum number of iterations. Therefore, when $0.2<t/t_{max}<0.4$, the algorithm enters Phase 2, and the candidate solutions are divided into two parts and updated using different formulas, which can be expressed by Equation (4).

(4) $$\left\{\begin{array}{c} \vec{NewX_{i}}=\vec{BB}+rand\left(1-\frac{t}{t_{max}}\right)\left(\vec{{RN}_{2}}⊗(r2\cdot \vec{BB}-\vec{{XS1}_{i}})\right)\\ \vec{NewX_{i}}=\vec{X_{i}}+\vec{LD}⊗(\vec{BB}-\vec{LD}⊗\vec{{XS2}_{i}}) \end{array}\right.i=1,\ldots ,\;n$$

Equation (4) indicates that half of the value of $\vec{NewX_{i}}$ comes from the first mathematical equation, and the remainder comes from the second formula. Therefore, in the second stage of CBSO, the division of search agents is determined by Equation (4), where $\vec{LD}$ represents a random number vector with a Levy distribution, t and $t_{max}$ represent the current iteration number and the maximum iteration number, respectively. $\vec{{XS1}_{i}}$ and $\vec{{XS2}_{i}}$ are two search agents randomly selected during the iteration process, r2 is a random number between 0 and 1, and $\vec{{RN}_{2}}$ is a random number vector with a normal distribution.

### 2.4. Phase 3

The third stage of CBSO utilizes the algorithm’s development capabilities to search for promising regions during the optimization process for local searches, thereby obtaining a higher-quality solution. This can be specifically expressed as Equation (5).

(5) $$\vec{NewX_{i}}=\vec{BB}+rand\left(1-\frac{t}{t_{max}}\right)\left(\vec{LD}⊗(\vec{LD}⊗\vec{XS1_{i}}-\vec{XS2_{i}})\right)i=1,\ldots ,\;n$$

**Figure 1:**
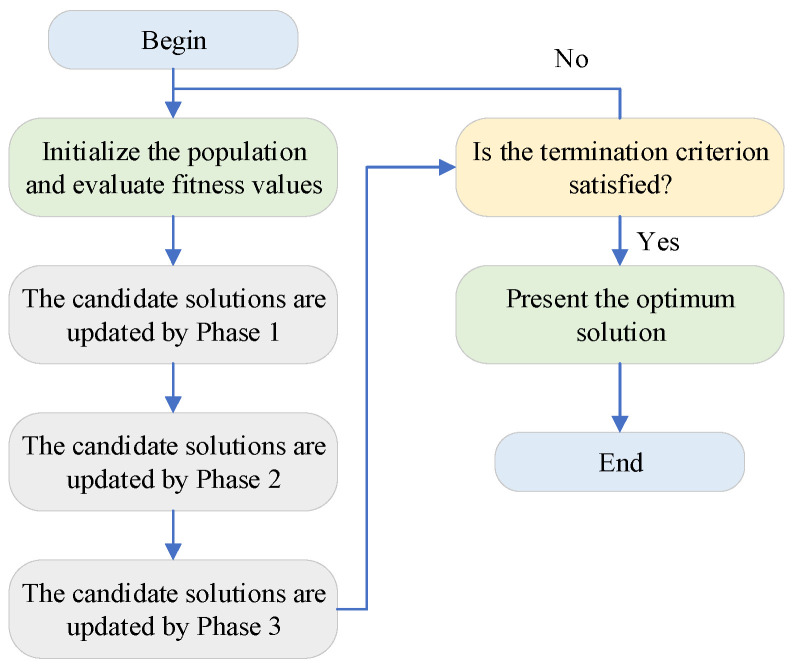
CBSO Execution Flowchart.

To improve the performance of CBSO, in addition to the mechanism for updating solutions at each search stage of the algorithm, CBSO also employs a complementary update mechanism, which performs a more robust update mechanism in the search space. This complementary update is achieved by simulating potential failures in the financial banking system. Specifically, it can be described as follows: assuming a 20% probability of system collapse, if no collapse occurs, CBSO will consider the possibility of a network attack on the system, which may sometimes even terminate the financial banking system. Therefore, CBSO encodes the solution in a way that its mathematical model can be expressed as Equation (6).

(6) $$\left\{\begin{array}{c} \vec{UPX_{i}}=\vec{NewX_{i}}+rand\left(rand\left(1-\frac{t}{t_{max}}\right)LB+rand\left(UB-LB\right)\right)ifRU<0.2\\ \vec{UPX_{i}}=\vec{NewX_{i}}+(rand.rand)(\vec{XS1_{i}}-\vec{XS2_{i}})Otherwsie. \end{array}\right.$$

where UB and LB represent the upper and lower bounds of the decision variable, respectively, RU is a random number between 0 and 1, and $\vec{UPX_{i}}$ represents the updated solution considering the collapse and encoding mechanisms. Figure 1 shows the execution flowchart of CBSO.

## 3. Proposed MICBSO

While CBSO performs well on 15-, 18-, and 25-rod problems, No Free Lunch theorems state that each algorithm requires modification for specific problems to improve its performance. Therefore, this paper proposes three effective strategies to enhance CBSO, described in detail below.

Throughout this paper, random variables are denoted in a unified manner. Unless otherwise specified, r, $r_{1}$, $r_{2}$, and $r_{u}$ represent mutually independent scalar random variables uniformly distributed in (0, 1). Similarly, r denotes a random vector whose elements are independently sampled from U(0, 1). The vectors $n_{1}$ and $n_{2}$ denote Gaussian random vectors sampled from N(0, I), and L denotes a Lévy-distributed random vector. In addition, $x_{0}$ is used only as the initial seed of the Logistic chaotic map, and it is randomly sampled from (0, 1). With this notation, repeated explanations such as “a random number between 0 and 1” are omitted in the following equations.

### 3.1. Population Initialization Strategy Based on Chaos-Opposition Learning

During the population initialization phase, the CBSO employs a random initialization method to obtain the initial population, which may result in inconsistent quality among initial populations [15]. To further improve the distribution quality and global exploration capability of the initial population, MICBSO introduces a joint strategy of Chaotic Map and Opposition-based Learning (OBL) in the algorithm initialization stage, forming a more diverse and globally comprehensive initialization method. In this method, a chaotic sequence is first generated through a Logistic mapping [16], which can be specifically represented by Equation (7).

(7) $$x_{t+1}=f\left(x_{t}\right)=\mu x_{t}\left(1-x_{t}\right),\;\;x_{t}\in (0,1)$$

where μ is a control parameter with a value of 4. In this study, the Logistic map with μ = 4 is adopted to generate the chaotic sequence for population initialization. The main reason is that, when μ = 4, the Logistic map exhibits strong chaotic behavior, simple mathematical implementation, and low computational cost, which makes it suitable for large-scale repeated optimization experiments. Compared with some other chaotic maps, such as Tent, Sine, Circle, and Chebyshev maps, the Logistic map does not require complicated parameter adjustment and can generate a sufficiently irregular sequence within the interval (0, 1). This property is consistent with the initialization requirement of MICBSO, where the chaotic sequence is directly mapped into the search space to improve population coverage. $x_{0}$ is a random number between 0 and 1, and then through long-term iteration, a uniformly distributed sequence is obtained. The initial value $x_{0}$ is an important factor affecting the trajectory of a chaotic sequence. In the proposed MICBSO, $x_{0}$ is not treated as a manually tuned control parameter, but is randomly generated within the interval (0, 1) for each independent run. This design avoids the dependence of the algorithm on a fixed chaotic seed and reduces the risk that the initial population is biased by a specific $x_{0}$. In addition, μ is set to 4 so that the Logistic map enters a fully chaotic state, enabling the generated sequence to exhibit strong randomness and ergodicity. To avoid degenerate cases, $x_{0}$ should not be set to boundary or special values such as 0, 0.25, 0.5, 0.75, or 1, which may lead to fixed points or short-period behavior. Since $x_{0}$ is randomly sampled in each run and all algorithms are independently executed 30 times, the influence of different initial chaotic seeds is statistically averaged in the reported results. Therefore, the performance of MICBSO is not determined by a single fixed $x_{0}$, but by the overall population distribution generated through random chaotic initialization and opposition-based learning. Then, by linearly mapping $x_{t}$ to the search space of the current dimension, we can obtain the initial search agent, which can be expressed by Equation (8). To clarify the rationale for selecting the Logistic map, Table 1 compares several commonly used chaotic maps across dimensions such as mathematical form, parameter settings, implementation complexity, and applicability to the proposed MICBSO initialization framework.

(8) $$X_{i,j}={lb}_{j}+x_{t}({ub}_{j}-{lb}_{j}),\;\;\left\{\begin{array}{c} i=1,2,\ldots ,n.\\ j=1,2,\ldots ,d. \end{array}\right.$$

Compared with traditional random initialization, chaotic mapping has better ergodicity and uniformity of distribution, which can avoid the initial solutions from being concentrated in local regions of the search space, thus providing a higher quality starting point for subsequent global searches.

After obtaining the chaotic initial population, this paper further introduces an opposition learning mechanism to construct its opposition solution for each individual in order to expand the search range. For the i-th individual, its opposite individual $X_{i}^{op}$ is calculated using Equation (9).

(9) $$X_{i,j}^{op}={lb}_{j}+{ub}_{j}-X_{i,j},\;\;\left\{\begin{array}{c} i=1,2,\ldots ,n.\\ j=1,2,\ldots ,d. \end{array}\right.$$

It can be seen that $X_{i}$ and $X_{i}^{op}$ are symmetrical about the center of the interval $\frac{{lb}_{j}+{ub}_{j}}{2}$. Thus, while generating a candidate solution, a “mirror solution” on the other side of the search space is also generated, which is equivalent to sampling the search space from two directions at the same time. Subsequently, fitness was calculated for the original individual and its counterpart using Equation (10).

(10) $$\left\{\begin{array}{c} f_{i}=f(X_{i})\\ f_{i}^{op}=f(X_{i}^{op}) \end{array}\right.$$

In this study, a one-to-one competition selection mechanism is adopted after evaluating the original and opposite solutions. Specifically, each original individual $X_{i}$ only competes with its corresponding opposite individual $X_{i}^{op}$, rather than selecting the best N individuals from the combined 2N candidates. For a minimization problem, the final initialized individual is determined as follows:

(11) $$X_{i}^{init}=\left\{\begin{array}{c} X_{i},\mathrm{if}f(X_{i})\leq f(X_{i}^{op}),\\ X_{i}^{op},otherwise. \end{array}\right.$$

Through this one-to-one selection rule, the population size remains N, and each original–opposite pair contributes one candidate solution to the final initial population. Compared with selecting the best N individuals from all 2N candidates, the one-to-one competition avoids excessive concentration of the initial population around several high-quality regions, thereby preserving the complementary sampling property of opposition-based learning. Meanwhile, this selection process does not introduce additional sorting operations and only requires 2N fitness evaluations during initialization.

Following the principle of “survival of the fittest,” individuals with better fitness were selected as the final initialization individuals. In summary, the proposed chaotic-opposition joint initialization strategy generates initial candidate solutions through a Logistic chaotic mapping, ensuring good ergodicity and uniform distribution of solutions in the search space. For each candidate solution, an opposite solution is constructed and selectively retained. Sampling is performed from two directions in the search space to expand the search range. The resulting initial population exhibits both high diversity and high average fitness. Compared to traditional random initialization methods, the chaotic-opposition joint strategy significantly improves the algorithm’s global search capability, reduces the probability of the algorithm getting trapped in local optima, and accelerates early convergence.

As shown in Table 1, although Tent, Sine, Circle, and Chebyshev maps also have chaotic properties and can be used for population initialization, they may introduce piecewise computation, additional control parameters, stronger oscillation, or extra normalization procedures. In contrast, the Logistic map with μ = 4 has a compact mathematical expression, requires no additional parameter adjustment, and can generate chaotic sequences directly within the interval (0, 1). This makes it suitable for the proposed chaos–opposition learning initialization strategy, where the chaotic individuals and their opposite solutions are jointly evaluated to improve initial search coverage. Therefore, the Logistic map is selected as the chaotic sequence generator in MICBSO.

### 3.2. Multi-Elite Guidance Mechanism Based on Gaussian Perturbation

To enhance the exploitation capability of CBSO while maintaining sufficient global search ability, a Gaussian perturbation-based multi-elite guidance mechanism is proposed in MICBSO. In the original CBSO, the population update process is strongly influenced by the current best solution. Although this mechanism can accelerate convergence, it may also cause excessive dependence on a single elite individual. Once the current best solution is located near a local optimum, the search direction of the whole population may be misled, resulting in premature convergence and insufficient population diversity.

To alleviate this problem, MICBSO introduces a multi-elite guidance mechanism. Instead of using only the current global best solution as the guiding point, the proposed mechanism uses the average information of the top K elite individuals to construct a more stable guidance vector. Meanwhile, Gaussian perturbation is introduced to provide stochastic search ability around the elite region, which helps the population escape local optima and improves the balance between exploration and exploitation. The position update formula of the proposed multi-elite guidance mechanism is expressed as follows:

(12) $$X_{i}(t+1)=X_{i}(t)+\alpha (t)\cdot \left(E(t)-X_{i}(t)\right)+\beta (t)\cdot \Delta_{i}(t),$$

where $X_{i}(t)$ denotes the position of the i-th individual at iteration t, E(t) is the multi-elite guidance vector, and $\Delta_{i}(t)$ represents the Gaussian perturbation direction of the i-th individual. The adaptive coefficients α(t) and β(t) are used to control the influence of multi-elite guidance and Gaussian perturbation, respectively.

The coefficient α(t) controls the attraction strength toward the multi-elite guidance center. It is defined as a linearly decreasing function:

(13) $$\alpha (t)=\alpha_{0}\left(1-\frac{t}{t_{max}}\right)$$

where $\alpha_{0}$ is the initial guidance coefficient, t is the current iteration number, and $t_{max}$ is the maximum number of iterations. In the early stage, a larger α(t) helps individuals move toward promising regions identified by multiple elites. As the iteration proceeds, α(t) gradually decreases, which prevents excessive attraction to the elite center and reduces the risk of population aggregation in the later stage.

The coefficient β(t) controls the influence of the Gaussian perturbation term. It is defined as an exponentially decreasing function:

(14) $$\beta (t)=\beta_{0}exp\left[-k{\left(\frac{t}{t_{max}}\right)}^{2}\right]$$

where $\beta_{0}$ is the initial perturbation coefficient and k is the exponential decay factor. The exponential decay form enables the Gaussian perturbation to play a stronger role in the early search stage and decrease rapidly in the middle and later stages. Therefore, the algorithm can maintain stronger exploration ability at the beginning and reduce unnecessary random disturbance during local refinement.

In Equation (12), $\Delta_{i}(t)$ denotes the perturbation direction generated by the Gaussian-mutated elite solution. To clarify its relationship with the Gaussian-mutated solution, $X_{mut}(t)$ is first constructed around the current best solution:

(15) $$X_{mut}(t)=X_{best}(t)+\alpha (t)\cdot N(0,I)$$

where $X_{best}(t)$ denotes the current best solution, and N(0,I) represents a standard Gaussian random vector with zero mean and unit variance. Then, the perturbation direction of the i-th individual is defined as the difference between the Gaussian-mutated elite solution and the current individual:

(16) $$\Delta_{i}(t)=X_{mut}(t)-X_{i}(t)=X_{best}(t)+\alpha (t)\cdot N(0,I)-X_{i}(t)$$

Thus, Equation (12) can be further understood as a combination of two search directions: the first one is the attraction direction from the current individual to the multi-elite guidance center E(t), and the second one is the stochastic perturbation direction from the current individual to the Gaussian-mutated elite solution $X_{mut}(t)$. This formulation enables MICBSO to exploit high-quality elite information while maintaining a certain degree of randomness.

The multi-elite guidance vector E(t) is calculated by averaging the positions of the top K elite individuals:

(17) $$E(t)=\frac{1}{K}\sum_{r=1}^{K}\;X^{\left(r\right)}(t)$$

where $X^{\left(r\right)}(t)$ denotes the r-th elite individual after ranking the population according to fitness values. In this study, K=5 is adopted. Compared with single-best guidance, the use of multiple elite individuals can reduce the risk of search bias caused by one potentially misleading best solution. However, if K is too small, the algorithm may still suffer from excessive dependence on a few individuals; if K is too large, medium-quality individuals may weaken the guidance effect of high-quality solutions. Therefore, a parameter sensitivity analysis for K is conducted in Section 4.8 to justify this setting.

Although N(0,I) in Equation (15) is a standard Gaussian random vector with fixed unit variance, the actual perturbation intensity in MICBSO is not fixed. This is because the Gaussian random vector is scaled by α(t) in Equation (15), and its influence on the final position update is further controlled by β(t) in Equation (12). According to Equations (12), (15) and (16), the stochastic component introduced into the position update is mainly determined by

(18) β(t)α(t)N(0,I)

Therefore, the effective variance of the stochastic Gaussian perturbation can be expressed as

(19) $$\sigma_{eff}^{2}(t)={\left[\alpha (t)\beta (t)\right]}^{2}$$

Since α(t) decreases linearly and β(t) decreases exponentially with the iteration process, the effective perturbation variance $\sigma_{eff}^{2}(t)$ also gradually decreases. In the early stage, a relatively large effective variance helps the population explore a wider search region and avoid premature convergence. In the later stage, the reduced effective variance weakens random disturbance and allows the algorithm to focus on local exploitation around promising regions. Therefore, although the standard Gaussian distribution itself has a fixed unit variance, the effective perturbation strength in MICBSO is adaptively controlled by α(t) and β(t).

The parameters $\alpha_{0}$, $\beta_{0}$, and k are used to determine the decreasing trend of the multi-elite guidance strength and Gaussian perturbation intensity. In this study, $\alpha_{0}=0.8$, $\beta_{0}=1$, and k=5 are adopted. Specifically, $\alpha_{0}=0.8$ provides a relatively strong but not excessive attraction toward the multi-elite guidance center in the early search stage. Since α(t) gradually decreases to 0 as the iteration proceeds, it helps avoid excessive elite attraction in the later stage. The value $\beta_{0}=1$ keeps the initial Gaussian perturbation at a normal scale, while k=5 makes the perturbation term decrease rapidly in the middle and later stages. For example, when $t/t_{max}=0.5$, β(t)=exp(−1.25)≈0.2865, and when $t/t_{max}=1$, β(t)=exp(−5)≈0.0067. Therefore, Gaussian perturbation plays a stronger role in early exploration and becomes very weak during late exploitation.

The reason for adopting linear decay for α(t) and exponential decay for β(t) is that these two coefficients control different search behaviors. The multi-elite guidance term should decrease smoothly to avoid abrupt changes in the search direction; therefore, a linear decay function is used for α(t). In contrast, Gaussian perturbation should be strong in the early stage but rapidly weakened in the later stage to improve convergence stability; therefore, an exponential decay function is used for β(t). Compared with other adaptiv schedules, such as polynomial decay, cosine decay, sigmoid decay, or feedback-based adaptive adjustment, the adopted linear–exponential combination has a simpler structure and introduces fewer additional parameters. This helps MICBSO achieve a smooth transition from global exploration to local exploitation while maintaining low parameter complexity.

To further clarify the selection of the adaptive schedules, Table 2 provides a qualitative comparison of several commonly used decay functions.

As shown in Table 2, alternative adaptive schedules may provide more flexible adjustment, but they usually introduce additional parameters or require extra monitoring of the search process. In contrast, the proposed linear–exponential decay design provides a simple and effective two-level control mechanism. The linear decay of α(t) ensures that the multi-elite guidance strength decreases smoothly, while the exponential decay of β(t) rapidly reduces the influence of Gaussian perturbation in the later stage. Therefore, this mechanism can maintain sufficient exploration in the early stage and enhance convergence stability in the later stage without significantly increasing parameter complexity.

### 3.3. Hybrid Boundary Control Strategy Based on Random Reflection

In CBSO, it is common for individuals to go out of bounds during the search process. Out-of-bounds individuals not only lead to infeasible solutions but may also cause the loss of search direction information, resulting in premature convergence and insufficient utilization of the solution space. In particular, the CBSO algorithm has a strong tendency for jump-up updates; therefore, designing a robust and efficient boundary handling mechanism is crucial for improving algorithm performance. To this end, this paper adopts a hybrid boundary strategy that combines reflective boundary control with a random reset mechanism, which takes into account the feasibility of understanding, the consistency of search direction, and the maintenance of population diversity [22].

For boundary crossing situations, MICBSO first adopts a boundary reflection strategy. When an individual exceeds the search interval in a certain dimension, the boundary is used as the axis of symmetry to reflect the position back to the feasible region, which can be expressed as Equation (20).

(20) $$X_{ij}=\left\{\begin{array}{c} 2ub_{j}-X_{ij},\;\;if\;\;X_{ij}>ub_{j}\\ 2lb_{j}-X_{ij},\;\;if\;\;X_{ij}<lb_{j}\\ X_{ij},\;\;otherwise \end{array}\right.$$

**Figure 2:**
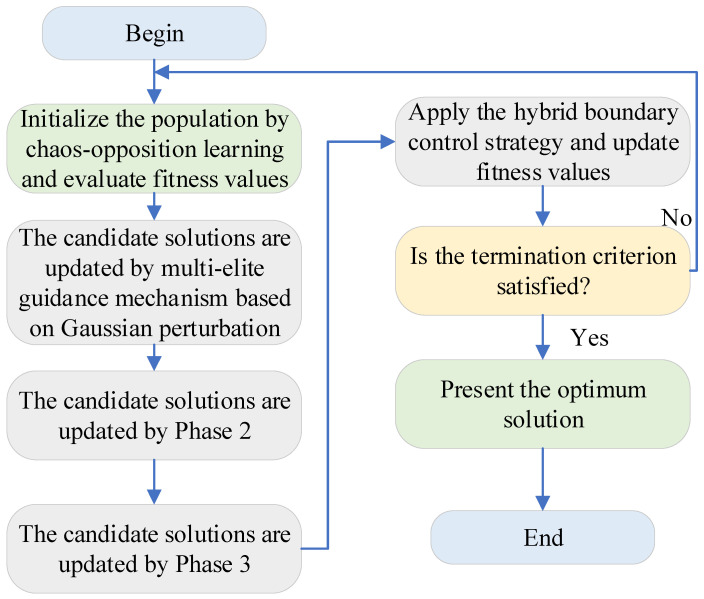
Flowchart of the proposed MICBSO.

Furthermore, while reflective boundaries can handle most out-of-bounds situations, for individuals with large overshoots or repeated violations, reflection alone may cause the individual to oscillate near the feasible region boundary. To avoid this problem, MICBSO introduces a random reinitialization strategy, which resamples the dimensions of large overshoots into the search space, as expressed in Equation (21).

(21) $$X_{ij}=lb_{j}+rand\cdot \left(ub_{j}-lb_{j}\right),$$

where rand is a random number between 0 and 1. Combining the two strategies mentioned above, MICBSO’s boundary control mechanism can be expressed as Equation (22).

(22) $$X_{ij}=\left\{\begin{array}{c} 2ub_{j}-X_{ij},\;\;0<X_{ij}-ub_{j}<\delta \\ 2lb_{j}-X_{ij},\;\;0<lb_{j}-X_{ij}<\delta \\ lb_{j}+rand\left(0,1\right)\left(ub_{j}-lb_{j}\right),\;\;otherwise \end{array}\right.$$

where δ is the reflection threshold, used to distinguish between minor and severe boundary violations. Specifically, it can be expressed by Equation (23). Where λ=0.2. Figure 2 shows the flowchart of MICBSO. It should be noted that MICBSO does not apply multiple reflections for repeatedly out-of-bounds individuals. The reason is that repeated reflection may cause individuals with large violations to oscillate near the boundary or even remain infeasible under large overshoot conditions. Therefore, the proposed hybrid boundary control strategy only applies a single reflection to minor boundary violations. Specifically, when $0<X_{ij}-ub_{j}<\delta$ or $0<lb_{j}-X_{ij}<\delta$, the individual is reflected once back to the feasible region. Since $\delta =0.2(ub_{j}-lb_{j})$ is smaller than the search range, the reflected position will still fall within the feasible interval. For example, if $X_{ij}>ub_{j}$ and $X_{ij}-ub_{j}<\delta$, then the reflected value satisfies $ub_{j}-\delta <X_{ij}^{new}<ub_{j}$, which is feasible. For severe boundary violations, reflection is not used. Instead, the violated dimension is randomly reinitialized within the feasible interval according to Equation (21). This design avoids repeated boundary oscillation and ensures that the corrected position always satisfies the boundary constraint. In addition, after boundary handling, a final feasibility check is conducted to guarantee that all decision variables remain within $\left[lb_{j},ub_{j}\right]$.

(23) $$\delta =\lambda \cdot \left(ub_{j}-lb_{j}\right)$$

The threshold coefficient 0.2 is adopted to provide a moderate boundary-handling range. If the threshold is too small, most out-of-bounds individuals will be regarded as severe violations and randomly reinitialized, which may weaken the continuity of the search direction. If the threshold is too large, most boundary violations will be handled by reflection, which may cause individuals to oscillate near the boundary and reduce population diversity. Therefore, $\delta =0.2(ub_{j}-lb_{j})$ is used as a balanced setting between reflective correction and random reinitialization. To further justify this setting, a parameter sensitivity analysis of λ in $\delta =\lambda (ub_{j}-lb_{j})$ is conducted in Section 4.8. The results show that λ=0.20 achieves more stable convergence performance than too small or too large threshold settings.

Although chaotic initialization, opposition-based learning, Gaussian perturbation, and boundary control have been employed in some existing improved swarm intelligence algorithms, the proposed MICBSO differs from these methods in its algorithm-specific integration with the three-stage search mechanism of CBSO. Existing studies usually introduce these strategies as independent enhancement operators to improve either initialization quality, local perturbation, or boundary repair separately. In contrast, MICBSO constructs a coordinated optimization framework in which the chaos–opposition learning strategy first improves the distribution quality and search coverage of the initial banking entities; the Gaussian perturbation-based multi-elite guidance mechanism then replaces the single-best-oriented guidance tendency of CBSO with a more stable elite-group-driven search direction; and the hybrid boundary control strategy adaptively combines reflection and random reinitialization to preserve feasible search directions while avoiding repeated boundary stagnation. Therefore, the novelty of MICBSO does not lie in using these operators in isolation, but in coupling them with the banking-system-inspired search process of CBSO to form a stage-aware balance among initialization diversity, elite-guided exploitation, and feasibility preservation. This coordinated design is particularly suitable for high-dimensional numerical optimization and constrained three-dimensional UAV path planning, where premature convergence, infeasible solutions, and unstable search trajectories frequently occur.

To further clarify the difference between the proposed hybrid boundary control strategy and common boundary-handling methods, several representative techniques are compared in Table 3, including saturation, periodic mapping, random repair, pure reflection, and the proposed hybrid strategy.

Compared with saturation and periodic mapping, the proposed hybrid boundary control strategy can avoid excessive concentration near the boundary and does not assume periodic characteristics of the search space. Compared with pure random repair, MICBSO preserves useful search direction information for minor boundary violations through reflective correction. Compared with pure reflection, MICBSO avoids repeated boundary oscillation by applying random reinitialization to severe violations. Therefore, the proposed hybrid strategy provides a more flexible boundary-handling mechanism by balancing feasibility preservation, search continuity, and population diversity.

### 3.4. Time Complexity Analysis and Execution Time Comparison

To further analyze the computational cost introduced by the proposed improvement strategies, the time complexity of MICBSO is discussed in this subsection. Assume that the population size is N, the problem dimension is D, the maximum number of iterations is T, the number of selected elite individuals is K, and the computational cost of one fitness evaluation is $C_{f}$.

For the original CBSO, the population initialization requires O(ND) operations, and the corresponding fitness evaluation requires $O(NC_{f})$. During each iteration, the position update of all candidate solutions requires approximately O(ND), and the fitness evaluation requires $O(NC_{f})$. Therefore, the overall time complexity of CBSO can be approximately expressed as:

(24) $$O_{CBSO}=O(ND+NC_{f})+O\left(T(ND+NC_{f})\right)$$

For the proposed MICBSO, the chaos–opposition learning initialization strategy introduces additional computational cost in the initialization stage. Specifically, the Logistic chaotic map is first used to generate N chaotic candidate solutions, which requires O(ND). Then, opposition-based learning generates another N opposite candidate solutions, which also requires O(ND). Since both the chaotic individuals and their opposite solutions need to be evaluated, the number of fitness evaluations in the initialization stage increases from N to 2N. Therefore, the initialization complexity of MICBSO can be expressed as:

(25) $$O_{init}=O(2ND+2NC_{f})$$

Although the opposition-based learning mechanism doubles the number of fitness evaluations during initialization, this additional cost is only introduced once before the iterative search begins. In contrast, the main computational cost of metaheuristic algorithms usually comes from repeated fitness evaluations during the iterative optimization process. If each individual is evaluated once per iteration, the total number of fitness evaluations in the original CBSO is approximately $FE_{CBSO}=N(T+1),$ where N evaluations are used for initialization and NT evaluations are used during iterations. For MICBSO, due to the additional opposite solutions in the initialization stage, the total number of fitness evaluations becomes $FE_{MICBSO}=N(T+2).$ Thus, the relative increase in fitness evaluations caused by the chaos–opposition learning initialization strategy is

(26) $$\frac{FE_{MICBSO}-FE_{CBSO}}{FE_{CBSO}}=\frac{N}{N(T+1)}=\frac{1}{T+1}$$

In this study, the maximum number of iterations is set to T=1000. Therefore, the additional fitness evaluation cost introduced by opposition-based learning is approximately 1/1001, namely about 0.10% of the total fitness evaluations. This indicates that although the initialization stage requires more evaluations, the additional computational burden is very limited in the whole optimization process.

In the iterative stage, the Gaussian perturbation-based multi-elite guidance mechanism introduces several additional operations. First, the population needs to be ranked or selected to obtain the top K elite individuals. This process requires O(NlogN) if sorting is used. Then, the elite guidance vector E(t) is constructed using the selected K elite individuals, requiring O(KD). The Gaussian perturbation and position update operations require O(ND). Since K is much smaller than N in this study, and K=5 is adopted, the additional cost of constructing the elite guidance vector is relatively small. Therefore, the time complexity of the multi-elite guidance mechanism can be approximately expressed as:

(27) $$O_{elite}=O(NlogN+KD+ND)$$

The hybrid boundary control strategy checks and repairs each dimension of each candidate solution. Therefore, its computational complexity is $O_{boundary}=O(ND).$ Combining the above analyses, the total time complexity of MICBSO can be expressed as $O_{MICBSO}=O(2ND+2NC_{f})+O\left(T(ND+NC_{f}+NlogN+KD)\right).$

Since K≪N and the fitness evaluation cost $C_{f}$ is usually the dominant part in benchmark optimization and UAV path planning problems, the additional computational cost introduced by the proposed strategies does not change the overall order of complexity. Therefore, the asymptotic time complexity of MICBSO can be simplified as $O_{MICBSO}=O\left(T(ND+NC_{f}+NlogN)\right).$ Compared with the original CBSO, MICBSO introduces extra computational operations mainly from three aspects: opposite solution evaluation during initialization, elite selection and guidance vector construction, and hybrid boundary repair. However, the opposition-based learning strategy only increases the initialization evaluations once, the number of elite individuals K is small, and the boundary control strategy only involves simple dimension-wise judgment and correction. Therefore, the additional computational burden of MICBSO is acceptable. More importantly, these strategies improve the initial population quality, reduce the risk of premature convergence, and enhance solution feasibility, which can help the algorithm obtain better solutions within the same maximum iteration number.

The theoretical comparison between CBSO and MICBSO is summarized in Table 4.

From Table 4, it can be observed that MICBSO has a slightly higher computational cost than CBSO due to the introduced improvement strategies. However, the increased cost is mainly reflected in the initialization stage and simple auxiliary update operations. The chaos–opposition learning strategy doubles the number of fitness evaluations only during initialization, while the total additional evaluation ratio is only 1/(T+1). Therefore, under the experimental setting of T=1000, the additional computational burden is very small compared with the entire optimization process. This demonstrates that MICBSO improves optimization performance while maintaining acceptable computational complexity.

In addition, we tested the various algorithms on the CEC2017 test set and recorded their actual execution times; the experimental results are shown below.

As shown in Table 5, the execution time of all algorithms increases as the problem dimension grows from 30 to 100, indicating that higher-dimensional optimization problems require more computational resources. Compared with the original CBSO, MICBSO introduces additional operations, including chaos–opposition-based initialization, multi-elite guidance, Gaussian perturbation, and hybrid boundary control. Therefore, its runtime is slightly higher than that of CBSO. Specifically, the runtime of MICBSO is 23.94 s, 48.33 s, and 172.33 s for Dim = 30, Dim = 50, and Dim = 100, respectively, while the corresponding runtime of CBSO is 22.60 s, 43.49 s, and 150.38 s. The relative increases are approximately 5.93%, 11.13%, and 14.60%, respectively. This indicates that the additional computational cost introduced by the proposed improvement strategies is limited. The proof of the global convergence of MICBSO is presented in Appendix A.

Although MICBSO is not the fastest algorithm, its runtime remains within an acceptable range. For example, under Dim = 30 and Dim = 50, MICBSO is faster than several comparison algorithms, such as ESC, HHWOA, and IGWO. Under Dim = 100, MICBSO is still much faster than HHWOA and IGWO, and its runtime is close to ESC. More importantly, the slightly increased runtime mainly comes from auxiliary operations rather than repeated expensive fitness evaluations. The chaos–opposition-based initialization only increases the number of fitness evaluations once at the beginning, while the multi-elite guidance and boundary control strategies mainly involve simple vector operations and boundary checks. Therefore, the proposed strategies do not cause excessive computational burden.

Overall, the execution time results demonstrate that MICBSO achieves a reasonable trade-off between computational cost and optimization performance. Although it requires slightly more time than CBSO, the additional runtime is acceptable considering its improved convergence accuracy, robustness, and solution quality. Therefore, MICBSO maintains practical computational efficiency while enhancing the overall optimization capability.

## 4. Experimental Results and Detailed Analyses

In this section, we use the IEEE CEC2017 test set to evaluate the performance of MICBSO. First, we provide a brief introduction to the IEEE CEC2017 test set, then explain the parameter settings for all compared algorithms. Furthermore, we compare MICBSO with other optimization algorithms to demonstrate its performance. Finally, to determine whether there are significant differences between MICBSO and other algorithms, we employ two statistical tests. The specific details are as follows.

### 4.1. Benchmark Test Functions

The IEEE CEC2017 benchmark set is one of the most widely used and influential benchmarks in the field of evolutionary computation and metaheuristic optimization. This benchmark set includes a variety of representative benchmark functions, covering various optimization characteristics such as unimodal, multimodal, separable and non-separable, rotational, and complex combinatorial structures, comprehensively examining the adaptability and robustness of algorithms in different types of search spaces. Due to its reasonable difficulty setting, diverse problem structures, and widespread adoption in research, CEC2017 has become the mainstream standard benchmark platform for evaluating the performance of metaheuristic algorithms [27].

In this study, we chose to use the CEC2017 benchmark set for experimental analysis, primarily because it is a popular benchmark for demonstrating the performance of metaheuristic algorithms, ensuring strong comparability and persuasiveness of the experimental results. Furthermore, its diverse testing environment can fully verify the effectiveness and generalization ability of the proposed algorithm in complex optimization scenarios.

### 4.2. Competitor Algorithms and Parameters Setting

This section evaluates the performance of MICBSO by comparing it with 11 state-of-the-art algorithms, including Particle Swarm Optimization (PSO), Snake Optimization (SO), Rime Optimization Algorithm (RIME), Dhole Optimization Algorithm (DOA), Secretary Bird Optimization Algorithm (SBOA), Gold Rush optimizer (GRO), Escape optimization algorithm (ESC), weighted mean of vectors optimization algorithm (INFO), hyper-heuristic whale optimization algorithm (HHWOA), improved grey wolf optimizer (IGWO), Connected Banking System Optimizer (CBSO). To ensure fairness, the population size for all compared algorithms was set to 50, and the maximum number of iterations was set to 1000. To reduce randomness, each algorithm was run independently 30 times, and the results were averaged for analysis. Table 6 summarizes the parameter settings of these algorithms for easy reading.

### 4.3. Exploration and Exploitation Analysis

Exploration and exploitation are two fundamental search behaviors of metaheuristic algorithms. Exploration refers to the ability of an algorithm to investigate different regions of the search space, which helps avoid premature convergence and improves the probability of finding the global optimum. Exploitation refers to the ability to refine solutions around promising regions, which is essential for improving convergence accuracy. An effective optimization algorithm should maintain sufficient exploration in the early stage and gradually strengthen exploitation in the later stage, thereby achieving a proper balance between global search and local refinement.

To quantitatively evaluate the exploration and exploitation behaviors of MICBSO, the population diversity is used as the basis for measurement. Let Div(t) denote the population diversity at iteration t, which reflects the dispersion degree of individuals in the search space. A larger Div(t) indicates stronger exploration ability, while a smaller Div(t) indicates that the population is more concentrated and the algorithm is performing stronger exploitation. The maximum diversity during the whole optimization process is denoted as $Div_{max}$, which can be expressed as:

(28) $$Div_{max}=\underset{1\leq t\leq t_{max}}{max}\;Div(t).$$

Then, the exploration percentage and exploitation percentage at iteration t are calculated as follows:

(29) $$Exploration(t)=\frac{Div(t)}{Div_{max}}\times 100\%,$$

(30) $$Exploitation(t)=\frac{\left|Div(t)-Div_{max}\right|}{Div_{max}}\times 100\%.$$

According to the above definitions, a higher exploration percentage indicates that the population is widely distributed in the search space, while a higher exploitation percentage indicates that the population gradually converges toward promising regions. Therefore, the dynamic variation of these two indicators can effectively reflect the search behavior of MICBSO during the optimization process.

**Figure 3:**
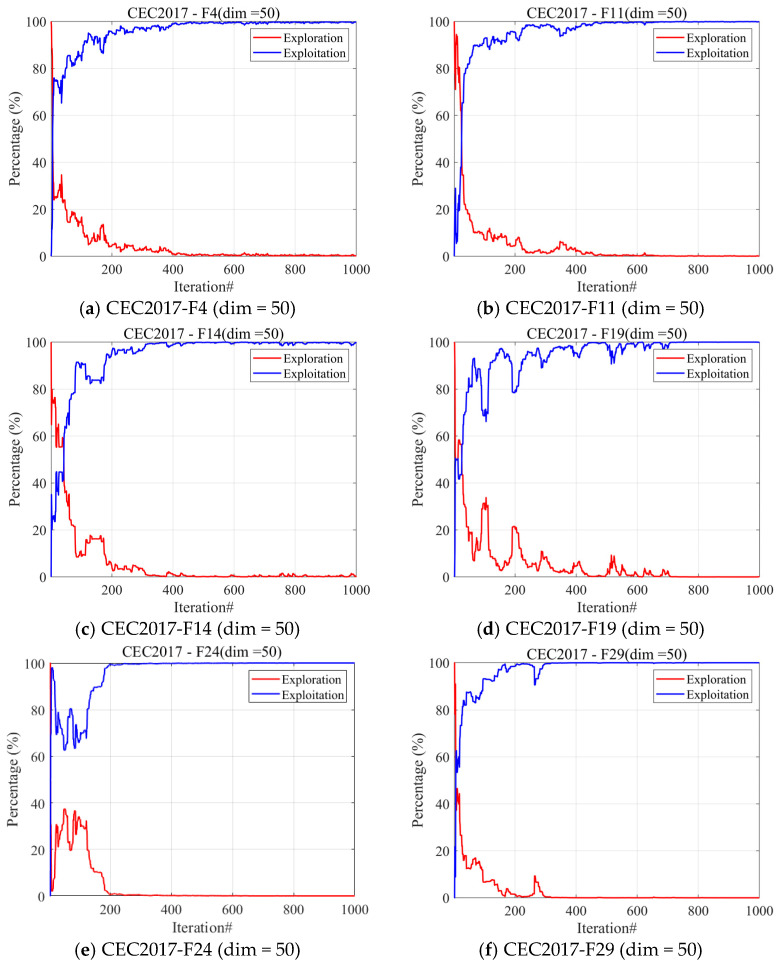
Exploration and exploitation analysis results of MICBSO on representative CEC2017 functions.

Figure 3 shows the exploration and exploitation curves of MICBSO on six representative CEC2017 benchmark functions with Dim = 50. It can be observed that MICBSO exhibits a clear transition from exploration to exploitation during the iterative process. In the early stage, the exploration percentage is relatively high, indicating that the population maintains sufficient diversity and can explore different regions of the search space. This behavior is mainly attributed to the chaos–opposition-based initialization strategy and Gaussian perturbation mechanism, which help expand the initial search coverage and enhance global search ability.

As the iteration proceeds, the exploration percentage gradually decreases, while the exploitation percentage increases rapidly and eventually remains at a high level. This indicates that MICBSO can effectively shift its search focus from global exploration to local exploitation. On F4, F11, F14, and F29, the exploitation percentage increases smoothly and approaches nearly 100% in the later stage, suggesting that the algorithm can gradually concentrate the population around high-quality regions and improve solution accuracy. On more complex functions such as F19 and F24, the exploration curves show several fluctuations in the early and middle stages, indicating that MICBSO still retains a certain level of exploratory behavior when facing complex landscapes. This helps the algorithm avoid premature convergence and search for more promising regions before final convergence.

Overall, the exploration and exploitation results demonstrate that MICBSO has a reasonable search transition mechanism. The proposed improvement strategies enable the algorithm to maintain sufficient exploration in the early stage and strengthen exploitation in the later stage. This balanced search behavior explains the good convergence performance and robustness of MICBSO on complex benchmark functions.

### 4.4. Population Diversity Analysis

Population diversity is an important indicator for evaluating the search behavior and robustness of metaheuristic algorithms. During the optimization process, sufficient population diversity enables the population to explore different regions of the search space and reduces the risk of premature convergence. If the diversity decreases too rapidly, individuals may gather around a local optimum at an early stage, resulting in insufficient global exploration and degraded solution quality. Conversely, if the diversity remains excessively high in the later stage, the algorithm may fail to effectively exploit promising regions and obtain high-precision solutions. Therefore, an effective optimizer should maintain relatively high diversity in the early search stage and gradually reduce diversity as the iteration proceeds, thereby achieving a reasonable balance between exploration and exploitation.

To quantitatively evaluate the population diversity of different algorithms, the diversity metric is calculated based on the average distance between individuals and the population center. Assume that the population size is N, the problem dimension is D, and $X_{i}(t)=[x_{i,1}(t),x_{i,2}(t),\;\ldots ,\;x_{i,D}(t)]$ represents the position of the i-th individual at iteration t. The center of the population in the j-th dimension can be calculated as follows:

(31) $${\bar{x}}_{j}(t)=\frac{1}{N}\sum_{i=1}^{N}\;x_{i,j}(t),j=1,2,\ldots ,D.$$

Then, the population diversity at iteration t is defined as:

(32) $$Div(t)=\frac{1}{N}\sum_{i=1}^{N}\sqrt{\frac{1}{D}\sum_{j=1}^{D}{\left(x_{i,j}\left(t\right)-{\bar{x}}_{j}(t)\right)}^{2}}.$$

where Div(t) represents the average dispersion degree of all individuals around the population center. A larger Div(t) indicates that individuals are more widely distributed in the search space, suggesting stronger exploration ability. In contrast, a smaller Div(t) means that individuals are more concentrated, indicating stronger exploitation behavior.

To facilitate comparison among different algorithms and different benchmark functions, the diversity value is further normalized as follows:

(33) $$NDiv(t)=\frac{Div(t)}{Div(0)}.$$

where Div(0) is the initial population diversity. Through this normalization, the diversity variation process of different algorithms can be compared on the same scale.

**Figure 4:**
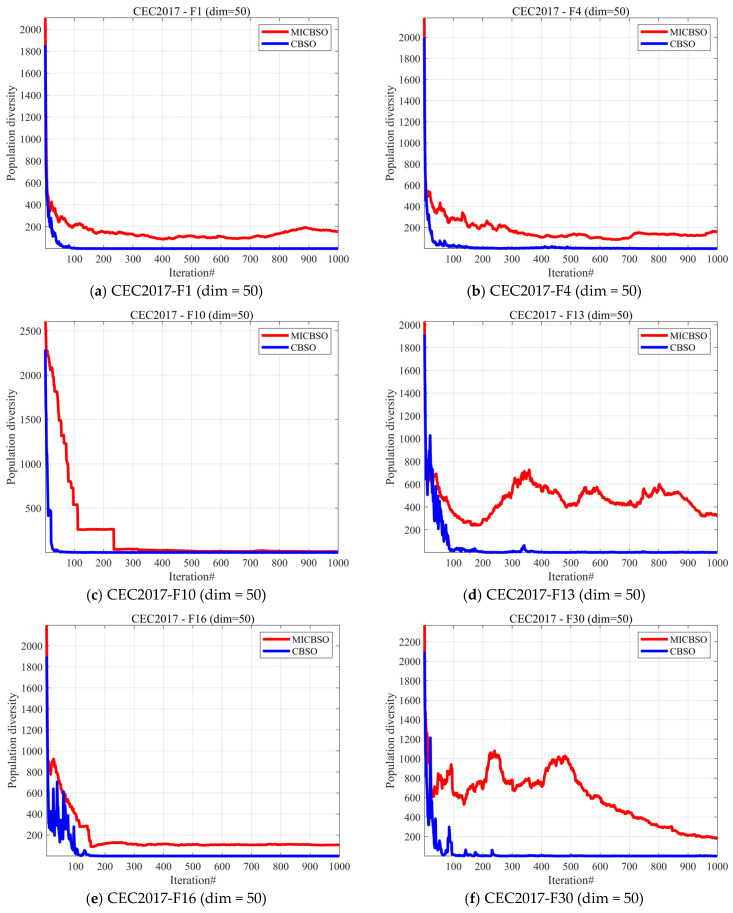
Population diversity curves of different algorithms on representative CEC2017 functions.

Figure 4 illustrates the population diversity curves of MICBSO and CBSO on six representative CEC2017 benchmark functions with Dim = 50. It can be observed that the population diversity of both algorithms decreases rapidly in the early stage, indicating that the population gradually shifts from global exploration to promising regions of the search space. However, compared with CBSO, MICBSO maintains a significantly higher diversity level on most test functions throughout the optimization process. In particular, on F1, F4, F13, F16, and F30, the diversity of CBSO drops sharply to a very low level within the first few iterations, suggesting that the original CBSO may suffer from rapid population aggregation and premature convergence. In contrast, MICBSO shows a smoother and more sustained diversity variation trend. This indicates that the chaos–opposition-based initialization strategy effectively expands the initial search coverage, while the Gaussian perturbation-based multi-elite guidance mechanism prevents the population from relying excessively on a single best individual. For complex functions such as F13 and F30, MICBSO still maintains relatively high diversity in the middle and later stages, which helps the algorithm continuously explore potential regions and avoid being trapped in local optima. Although the diversity of MICBSO also decreases as the iteration proceeds, it does not collapse prematurely like CBSO, demonstrating a better balance between exploration and exploitation. Therefore, the population diversity results further confirm that the proposed improvement strategies can enhance the search stability, maintain population diversity, and improve the robustness of MICBSO on complex optimization problems.

### 4.5. Performance Comparison on CEC2017 Benchmark Functions

**Figure 5:**
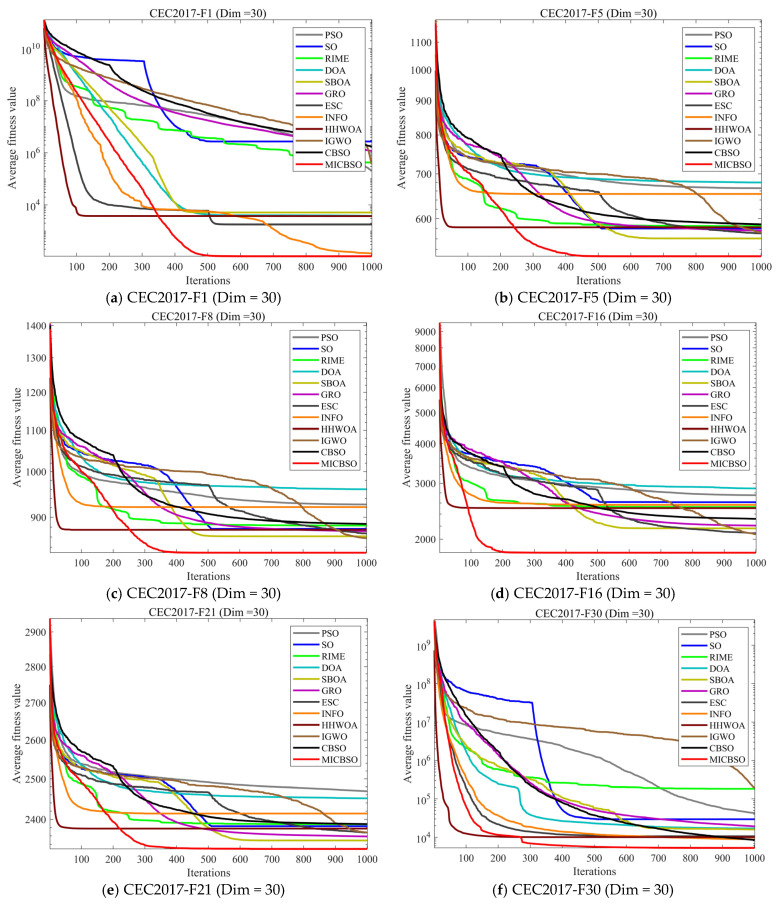

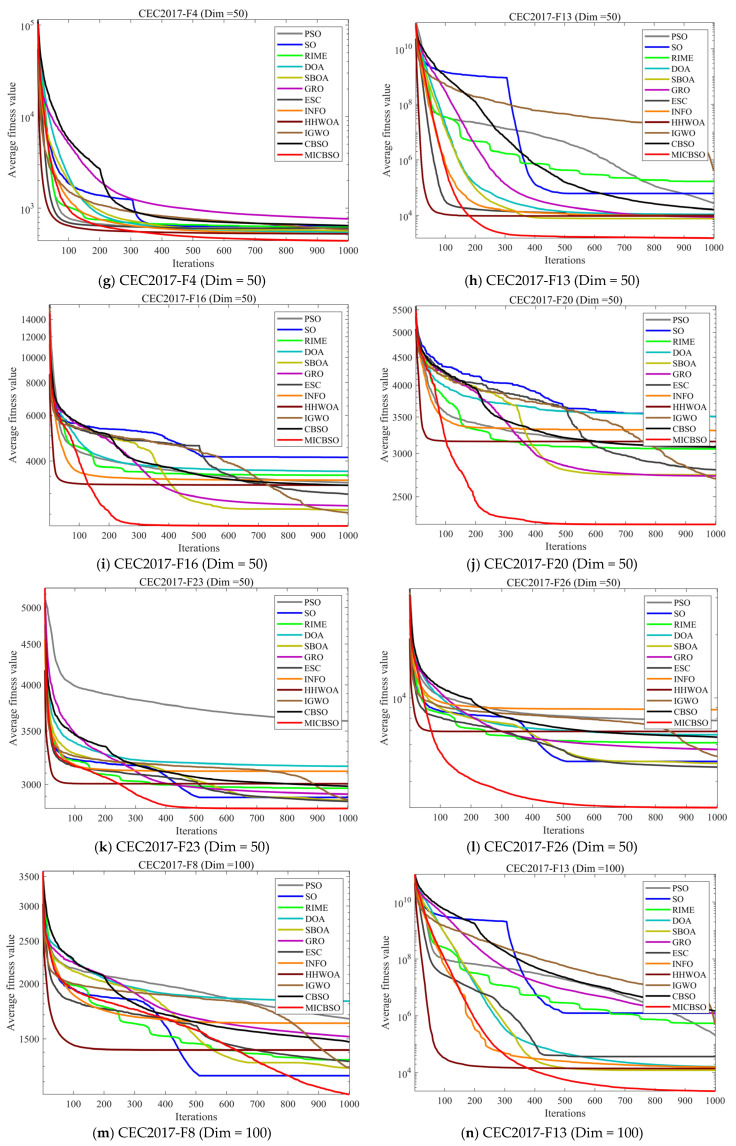

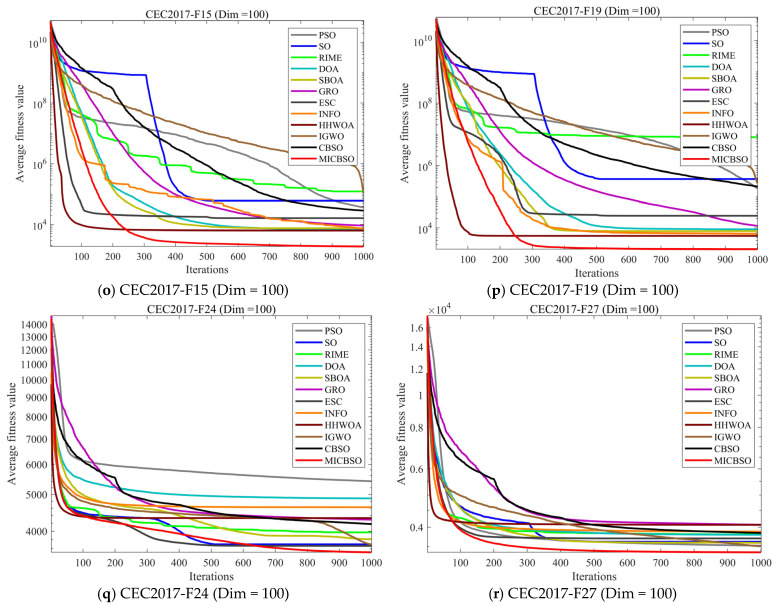
Comparison of convergence speed of different algorithms on CEC2017 test set.

**Figure 6:**
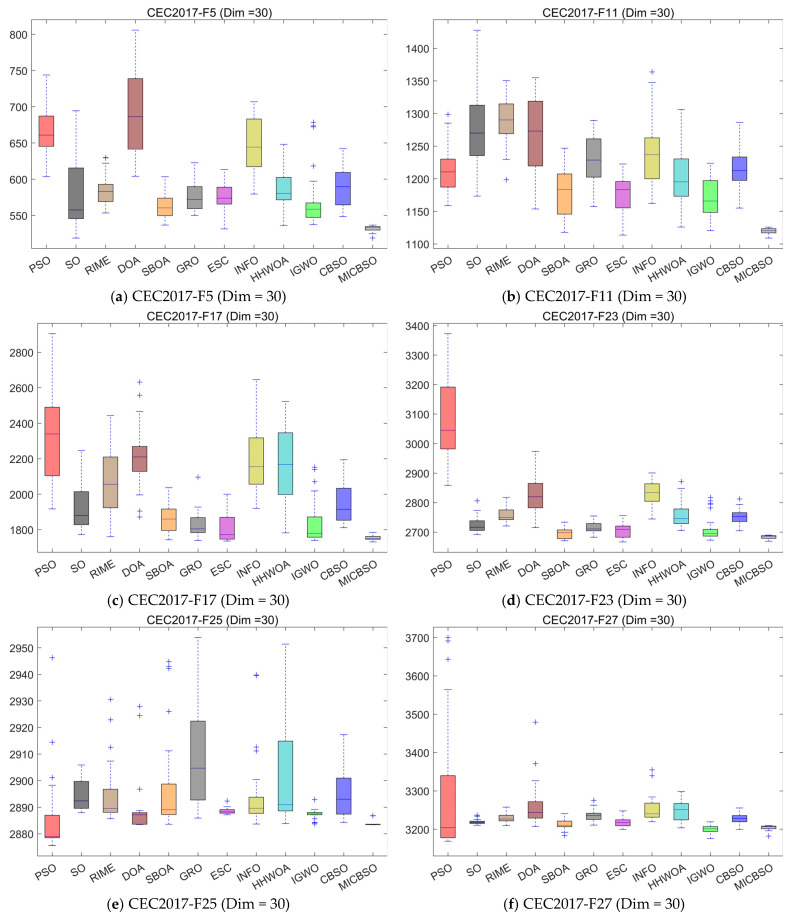

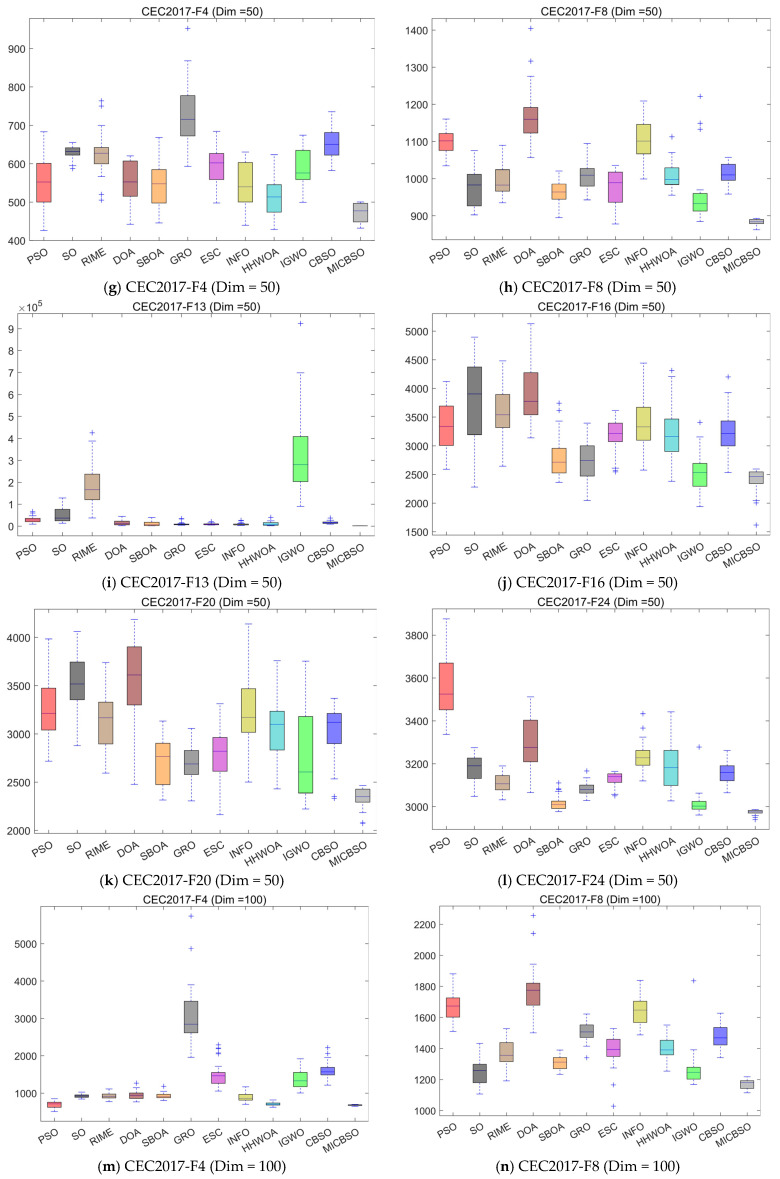

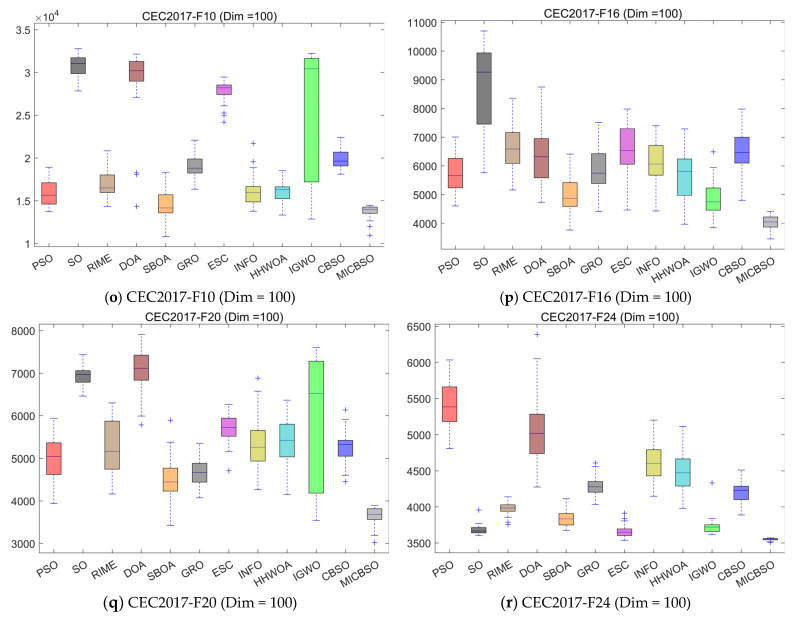
Boxplot analysis for different algorithms on the CEC2017 test set.

To test the performance of MICBSO, this section uses the CEC2017 test set for quantitative analysis experiments. We compare MICBSO with 11 benchmark algorithms. Figure 5 shows the average convergence curves of all 12 algorithms after 30 independent runs. Table 7, Table 8 and Table 9 list the performance metrics of these 12 algorithms on the CEC2017 test set in three dimensions. Here, “mean” represents the average of 30 independent runs, and “std” represents the standard deviation of the algorithm after 30 independent runs. To more intuitively illustrate the results and highlight the performance advantages of MICBSO, Figure 6 shows the box plots of each algorithm after 30 independent runs.

Figure 5 compares the convergence behavior of MICBSO and the other 11 algorithms on representative CEC2017 benchmark functions with different dimensional settings. Overall, MICBSO exhibits faster convergence speed and better final convergence accuracy on most test functions. In the early search stage, the fitness value of MICBSO decreases rapidly, indicating that the chaos–opposition learning initialization strategy provides a high-quality initial population and improves the global search efficiency. In the middle and later stages, MICBSO continues to refine the solutions and usually converges to lower objective values than the comparison algorithms, which demonstrates the effectiveness of the Gaussian perturbation-based multi-elite guidance mechanism in enhancing exploitation capability and avoiding premature convergence. In contrast, several comparison algorithms show slow convergence, stepwise stagnation, or insufficient final accuracy on some functions, especially in high-dimensional cases. For example, on representative multimodal, hybrid, and composition functions such as F13, F15, F16, F19, F20, and F26, MICBSO maintains a clear convergence advantage and reaches a lower fitness level within fewer iterations. Although the performance gap becomes smaller on several difficult high-dimensional functions, MICBSO still achieves competitive or superior convergence behavior, indicating that the proposed strategies can effectively improve both convergence speed and solution quality across different search landscapes. These results further confirm that MICBSO has a stronger balance between global exploration and local exploitation than the original CBSO and other comparison algorithms.

Figure 6 presents the boxplot results of different algorithms on representative CEC2017 benchmark functions after 30 independent runs. The boxplot can intuitively reflect both the optimization accuracy and stability of each algorithm, where a lower median value indicates better solution quality, and a smaller interquartile range represents stronger robustness. As shown in Figure 6, MICBSO generally achieves lower median fitness values and more compact box distributions than most comparison algorithms across different dimensional settings. In particular, on functions such as F5, F11, F17, F23, F24, F8, F16, and F20, the boxplots of MICBSO are located at relatively lower positions, indicating that it can obtain better solutions in repeated experiments. Meanwhile, the narrower boxes and shorter whiskers of MICBSO show that its optimization results fluctuate less across 30 runs, demonstrating better stability and reliability. In contrast, several comparison algorithms exhibit larger interquartile ranges or more outliers on some functions, suggesting that their performance is more sensitive to random initialization and complex search landscapes. This phenomenon is especially obvious on high-dimensional functions, where some algorithms show significantly dispersed distributions. Overall, the boxplot results further confirm that the proposed chaos–opposition learning initialization, Gaussian perturbation-based multi-elite guidance, and hybrid boundary control strategies can effectively improve the robustness of MICBSO and reduce the risk of unstable optimization performance.

Table 7, Table 8 and Table 9 report the mean and standard deviation values obtained by MICBSO and 11 comparison algorithms on the CEC2017 benchmark functions under 30-, 50-, and 100-dimensional settings. Overall, MICBSO demonstrates strong optimization accuracy and robustness across different dimensional cases. In the 30-dimensional experiments, MICBSO obtains the best mean values on 22 out of 30 functions and ranks within the top three on 27 functions, indicating its strong search capability in relatively low-dimensional problems. In the 50-dimensional case, MICBSO further maintains competitive performance, achieving the best results on 23 functions and ranking within the top three on 26 functions. For example, MICBSO achieves much lower mean fitness values on several complex functions such as F1, F10, F13, F16, F29, and F30, showing that the proposed strategies can effectively improve both global exploration and local exploitation. When the dimension increases to 100, the optimization difficulty becomes significantly higher, and the performance differences among algorithms become more obvious. Even under this challenging condition, MICBSO still obtains the best mean values on 18 functions and remains within the top three on 26 functions, demonstrating good scalability and robustness. Compared with the original CBSO, MICBSO achieves better mean values on most benchmark functions in all three dimensions, which verifies that the chaos–opposition learning initialization, Gaussian perturbation-based multi-elite guidance, and hybrid boundary control strategies can effectively enhance the original CBSO. In addition, MICBSO generally shows smaller or competitive standard deviations on many functions, suggesting that it can maintain stable performance over repeated independent runs. These results indicate that MICBSO has strong adaptability to unimodal, multimodal, hybrid, and composition functions, and can maintain reliable optimization performance as the problem dimension increases.

### 4.6. Statistical Analysis

To mitigate the impact of randomness in heuristic algorithms on experimental results and ensure the reliability and objectivity of comparative conclusions, it is necessary to conduct systematic statistical analysis of algorithm performance. Since heuristic algorithms may exhibit significant performance fluctuations across different runs, the results of a single experiment cannot fully reflect their true performance. Therefore, in this section, we conducted statistical tests and analyses on the algorithms involved. First, we used the Wilcoxon rank-sum test to perform pairwise comparisons of the performance differences among the 12 algorithms to determine whether the differences were statistically significant. Subsequently, we used the Friedman Mean Rank Test to evaluate the overall ranking performance of each algorithm, thereby providing a more comprehensive analysis of the relative performance of different algorithms across multiple sets of experiments. Specific statistical results are shown below.

#### 4.6.1. Wilcoxon Rank Sum Test

The Wilcoxon Rank Sum Test is a nonparametric statistical test commonly used to compare the differences between two independent samples in their location within a population distribution [38]. This method does not rely on the assumption of a normal distribution of the sample data, thus exhibiting strong robustness when dealing with experimental data that possesses randomness and non-normal distribution characteristics. The Wilcoxon Rank Sum Test determines whether a statistically significant difference exists between two groups by ranking the merged samples and comparing their rank sums; therefore, it is widely used in performance comparisons of heuristic algorithms and stochastic optimization algorithms. In this section, we performed a Wilcoxon Rank Sum Test on MICBSO. We set the significance level to 0.05. If *p* < 0.05, we reject the null hypothesis, indicating a significant difference; otherwise, we accept the null hypothesis, suggesting no significant difference. Table 10, Table 11 and Table 12 report the experimental results of 11 comparison algorithms across four evaluation dimensions on the CEC2017 benchmark set. The results indicate that MICBSO demonstrates statistically significant differences compared with most competing algorithms on the majority of test functions.

The statistical results reported in Table 10, Table 11 and Table 12 provide strong evidence for the superiority of MICBSO. The Wilcoxon rank-sum test results demonstrate that MICBSO achieves statistically significant performance improvements over most comparison algorithms on the majority of CEC2017 benchmark functions across different dimensional settings. Moreover, the Friedman Mean Rank Test shows that MICBSO consistently ranks first among all algorithms in 30-, 50-, and 100-dimensional problems, confirming its robustness and overall effectiveness in solving complex optimization tasks.

#### 4.6.2. Friedman Mean Rank Test

In this subsection, the Friedman Mean Rank Test was employed to analyze the performance of 12 algorithms [39]. As a nonparametric statistical test, it is widely used for comparing multiple algorithms over the same set of test problems without assuming a specific data distribution. The method ranks the performance of each algorithm on every test function and evaluates whether the observed differences in rankings are statistically significant. By averaging the ranks across all test functions, the overall relative performance of each algorithm can be effectively assessed. Table 13 shows the Friedman rankings of the 12 algorithms across three dimensions, where M.R represents the average ranking across the 30 functions, and T.R represents the final ranking of the 12 algorithms across the 30 functions.

The Friedman ranking results reported in Table 13 clearly demonstrate that MICBSO consistently achieves the best overall performance among all comparison algorithms across different dimensional settings. The lowest mean ranks and the first final rank obtained by MICBSO indicate its superior robustness and generalization ability when solving complex optimization problems.

### 4.7. Ablation Study Analysis

Ablation experiments are an important method for verifying the effectiveness of each improvement strategy in a proposed metaheuristic algorithm. Although the overall performance of MICBSO can be demonstrated through comparisons with other algorithms, it is still necessary to further analyze whether each introduced strategy contributes positively to the optimization process. Therefore, an ablation study is conducted in this subsection to evaluate the individual effects of the chaos–opposition learning initialization strategy, the Gaussian perturbation-based multi-elite guidance mechanism, and the hybrid boundary control strategy.

To clearly investigate the contribution of each strategy, several algorithm variants are constructed. The original CBSO is used as the baseline algorithm. CBSO+Chaos-OBL denotes the variant that only introduces the chaos–opposition learning initialization strategy. CBSO+Elite-Guidance denotes the variant that only introduces the Gaussian perturbation-based multi-elite guidance mechanism. CBSO+Boundary-Control denotes the variant that only introduces the hybrid boundary control strategy. MICBSO represents the complete algorithm integrating all three proposed improvement strategies. The ablation results are evaluated on the CEC2017 benchmark suite under 30-, 50-, and 100-dimensional settings. The Friedman mean rank and total rank are reported in Table 14, where a smaller rank indicates better overall performance.

As shown in Table 14, the original CBSO obtains the worst ranking results among all variants, with mean ranks of 3.73, 3.70, and 4.03 under 30-, 50-, and 100-dimensional conditions, respectively. This indicates that although CBSO has certain optimization capability, it still suffers from limitations in population initialization, search guidance, and boundary handling. After introducing the chaos–opposition learning strategy, CBSO+Chaos-OBL achieves better mean ranks than the original CBSO in all dimensional settings, demonstrating that the improved initialization strategy can enhance the distribution quality and search coverage of the initial population. This helps the algorithm obtain a more favorable starting point and improves its global exploration ability.

The CBSO+Elite-Guidance variant also shows clear improvement over the original CBSO. Its mean ranks are 3.30, 3.30, and 3.13 under 30, 50, and 100 dimensions, respectively. This result suggests that the multi-elite guidance mechanism can effectively reduce the dependence on a single best solution and provide a more stable search direction. By incorporating Gaussian perturbation, the algorithm can further enhance local refinement while maintaining a certain degree of randomness, which contributes to better convergence behavior.

For the CBSO+Boundary-Control variant, the mean ranks are 3.20, 3.37, and 2.93 under the three dimensional settings. Compared with CBSO, this variant achieves better ranking results, especially in the 100-dimensional case. This indicates that the hybrid boundary control strategy can effectively handle infeasible solutions generated during the search process. By combining reflective correction and random reinitialization, the strategy preserves useful search direction information for minor violations while maintaining diversity for severe boundary violations, thereby improving search stability.

Most importantly, the complete MICBSO obtains the best ranking results in all dimensional settings, with mean ranks of 1.40, 1.30, and 1.47, respectively. It ranks first under 30-, 50-, and 100-dimensional conditions, significantly outperforming all single-strategy variants. This demonstrates that the three proposed strategies are not simply independent enhancements, but can work cooperatively to improve the overall optimization capability of CBSO. Specifically, the chaos–opposition learning strategy improves initial population diversity, the multi-elite guidance mechanism strengthens search direction and exploitation capability, and the hybrid boundary control strategy enhances feasibility preservation and population stability. Their combined effect enables MICBSO to achieve a better balance between exploration and exploitation.

Overall, the ablation results confirm the effectiveness of each proposed improvement strategy. Each single strategy can improve the performance of CBSO to different degrees, while the complete MICBSO achieves the best overall performance. Therefore, the proposed three strategies are all necessary and complementary for enhancing the optimization accuracy, robustness, and scalability of MICBSO.

### 4.8. Parameter Sensitivity Analysis

Parameter sensitivity analysis is a crucial step in evaluating the robustness and applicability of meta-heuristic algorithms. An appropriate parameter configuration should ensure that the algorithm maintains stable convergence characteristics and achieves competitive optimization results across various test functions. Although the proposed MICBSO incorporates several improvement strategies to enhance population diversity, search guidance, and boundary handling, certain parameters within these strategies may influence the balance between exploration and exploitation. Therefore, it is essential to examine the sensitivity of MICBSO’s performance to these parameter settings. In this subsection, we conduct a parameter sensitivity analysis for MICBSO; the specific experimental results are presented below.

**Figure 7:**
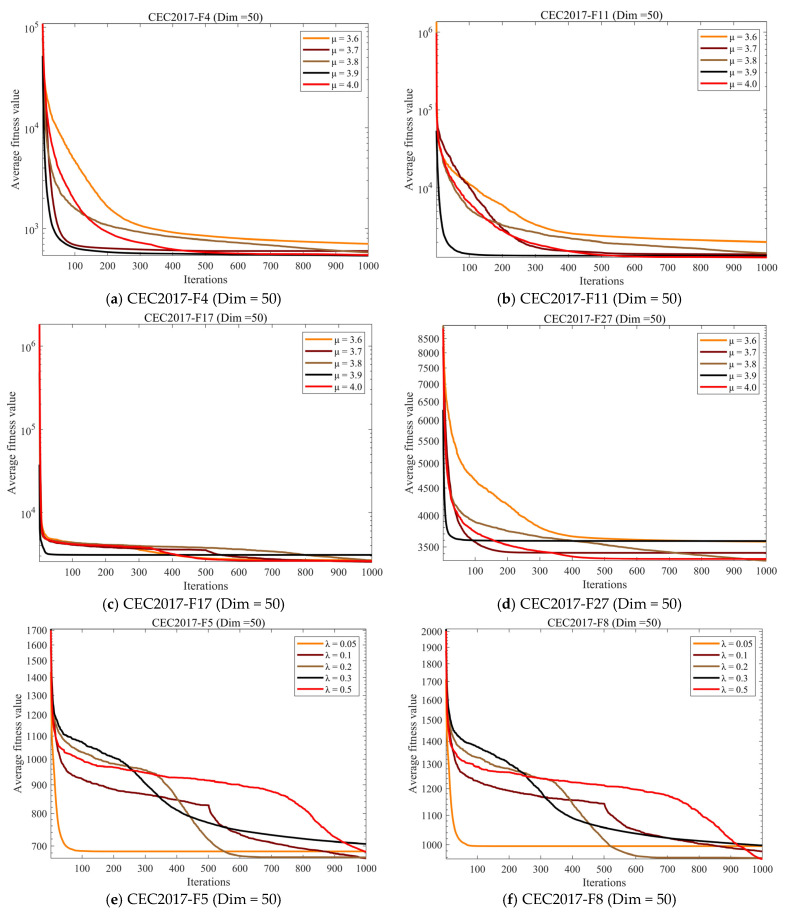

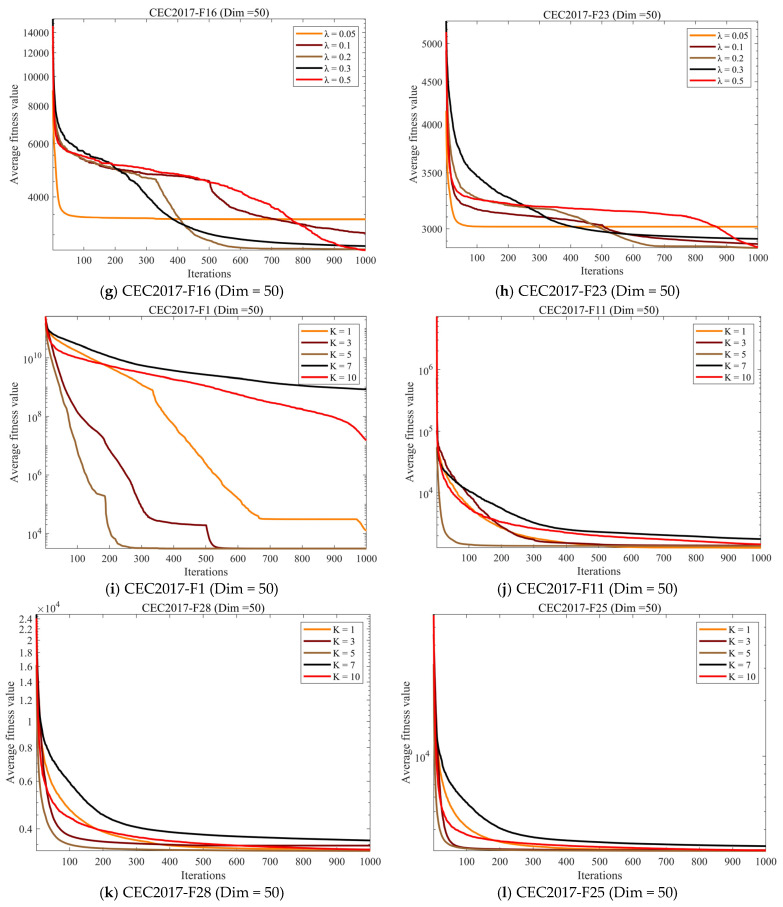
Experimental results of parameter sensitivity analysis.

Figure 7 presents the experimental results of the parameter sensitivity analysis for μ, λ, and K on representative CEC2017 benchmark functions with Dim = 50. As shown in Figure 7a–d, different values of μ affect the convergence behavior of MICBSO to a certain extent. When μ is relatively small, such as μ=3.6, the convergence speed is generally slower and the final fitness values are less competitive on several functions, indicating that insufficient chaotic intensity may weaken the quality of population initialization. In contrast, larger values of μ, especially μ=3.9 and μ=4.0, usually show faster convergence and better final optimization accuracy. This is because the Logistic map can generate stronger chaotic behavior when μ approaches 4, thereby improving the randomness and coverage of the initial population. Although μ=4.0 does not always achieve the best result on every single function, it maintains stable and competitive performance across different test functions. Therefore, μ=4.0 is adopted in MICBSO.

Figure 7e–h shows the influence of the reflection threshold coefficient λ in the hybrid boundary control strategy. It can be observed that different λ values lead to different boundary-handling behaviors. When λ is too small, such as λ=0.05, more out-of-bounds individuals are handled by random reinitialization. This may accelerate the early-stage decrease in the fitness value on some functions, but it may also weaken the continuity of the search direction and cause performance fluctuations in the later stage. When λ is too large, such as λ=0.5, more individuals are repaired by reflection, which helps preserve search direction information but may also increase the risk of boundary oscillation and slow down convergence on some functions. In comparison, the moderate setting λ=0.2 provides a better balance between reflective correction and random reinitialization, maintaining both search continuity and population diversity. Therefore, λ=0.2 is selected as the default threshold coefficient.

Figure 7i–l further illustrates the sensitivity of MICBSO to the number of elite individuals K. The results show that K has an obvious influence on the multi-elite guidance mechanism. When K=1, the algorithm degenerates into a nearly single-elite-guided search, which may lead to insufficient population information and unstable convergence. When K is too large, such as K=7 or K=10, more medium-quality individuals participate in the construction of the guidance vector, which may weaken the dominant role of high-quality elites and reduce convergence efficiency. In contrast, K=5 achieves faster convergence and more competitive final fitness values on most representative functions, especially on F1, F11, F28, and F25. This indicates that selecting five elite individuals can effectively balance elite guidance and population diversity. Overall, the sensitivity analysis demonstrates that the adopted parameter settings, namely μ=4.0, λ=0.2, and K=5, are reasonable and can help MICBSO maintain stable convergence performance across different types of benchmark functions.

## 5. MICBSO for Real Environment UAV Path Planning

In this section, we focus on applying the proposed MICBSO to unmanned aerial vehicle (UAV) path planning in real-world environments. First, a realistic UAV flight environment is constructed to reflect practical constraints and operational scenarios. Then, a fitness function is formulated by jointly considering key factors such as path length, flight safety, and environmental threat cost. Finally, comprehensive simulation experiments are conducted to evaluate the effectiveness and applicability of the proposed MICBSO approach. The detailed procedures are presented as follows.

The experiments in this section are conducted in simulation because real-world UAV flight tests require a complete physical UAV platform, onboard sensing and communication systems, safety control mechanisms, and suitable field-test conditions. Nevertheless, to improve the realism of the simulation environment, a LiDAR-based DEM is used as the basic terrain model, and different obstacle/threat distributions are introduced to emulate complex practical UAV navigation scenarios.

### 5.1. Scenarios and Objective Functions

In this section, the UAV flight environment and the corresponding objective function are formulated. The detailed modeling process and definitions are presented as follows.

#### 5.1.1. Scenario Setting

**Figure 8:**
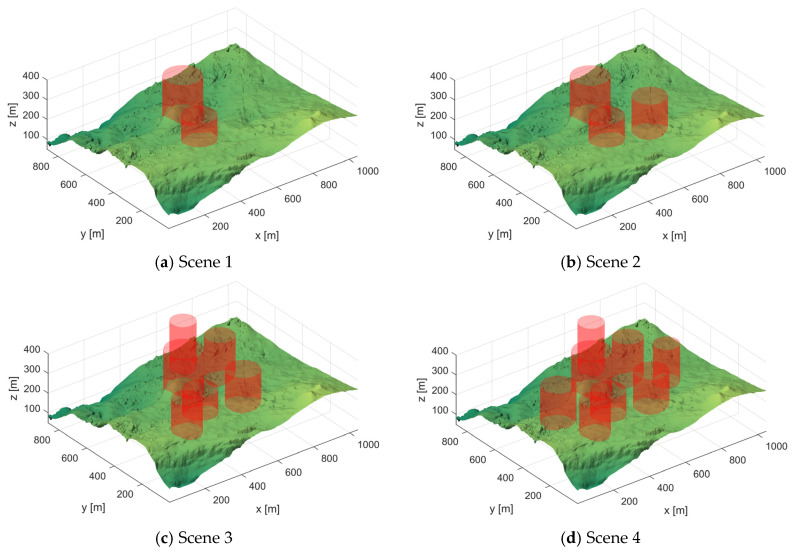
Four different scenario views.

In this section, a real-world digital elevation model (DEM) obtained from LiDAR sensing is adopted as the foundational flight environment. To emulate typical obstacles encountered in practical scenarios, cylindrical objects are introduced to represent environmental hazards such as trees. Through data augmentation, four representative UAV flight environments are constructed. Figure 8 illustrates schematic views of these environments, each characterized by different densities of hazardous elements, thereby enabling a comprehensive evaluation under diverse threat conditions commonly present in real-world environments.

#### 5.1.2. Optimization Problem Definition

In this section, the objective function for UAV path planning is formulated by jointly considering multiple cost components, including path length cost, threat cost, altitude cost, and smoothness cost. The detailed definitions and formulations are presented as follows.

1. Path Length Costs

In UAV path planning, path length is regarded as one of the most critical evaluation metrics, as it directly influences the UAV’s energy consumption and mission completion time. A shorter flight path generally leads to higher operational efficiency and improved safety, especially in complex environments. Therefore, minimizing path length constitutes a fundamental objective for ensuring reliable and efficient UAV operations.

Let the j-th waypoint of the i-th UAV be denoted as $P_{ij}=(x_{ij},y_{ij},z_{ij})$. The Euclidean distance between two consecutive waypoints is expressed as $∥\vec{P_{ij}P_{i,j+1}}∥$. Accordingly, the total flight cost of the UAV is calculated as shown in Equation (34).

(34) $$F_{1}(X_{i})=\sum_{j=1}^{n-1}\;∥\vec{P_{i,j}P_{i,j+1}}∥,$$

2. Threat Costs

In UAV path planning, threat cost is also a critical evaluation metric. If a path planning algorithm cannot effectively guide the UAV to avoid environmental obstacles and hazardous regions, its practical applicability is severely limited. Ensuring flight safety throughout mission execution is therefore a fundamental requirement for any reliable path planning approach. Accordingly, the threat cost of the UAV is formulated and computed as shown in Equation (35).

(35) $$F_{2}\left(X_{i}\right)=\sum_{j=1}^{n-1}\;\sum_{k=1}^{K}\;T_{k}\left(\vec{P_{ij}P_{i,j+1}}\right),$$

where $T_{k}\left(p_{ij}p_{i,j+1}\right)$ denotes flight constraint costs, which is calculated by Equation (36).

(36) $$T_{k}\left(p_{ij}p_{i,j+1}\right)=\left\{\begin{array}{c} 0,\;\;\;\;\;\;d_{k}>S+D+R_{k}\\ \left(S+D+R_{k}\right)-d_{k},\;\;\;\;\;\;{D+R}_{k}<d_{k}\leq S+D+R_{k}\\ \infty ,\;\;\;\;\;\;d_{k}\leq {D+R}_{k} \end{array}\right.,$$

where $R_{k}$ denotes the radius of the Kth cylindrical obstacle, D denotes the peripheral collision region, and $d_{k}$ denotes the distance from the center of the obstacle to the path $L_{p_{ij}p_{i+1,j}}$.

3. High Costs

In practical UAV flight operations, variations in air density at different altitudes can significantly affect the UAV’s energy consumption. Moreover, altitude constraints are imposed in certain areas, such as restricted low-altitude zones near airports. Consequently, flight altitude must be carefully considered during path planning. Based on these considerations, the altitude cost of the UAV is formulated and calculated as shown in Equation (37).

(37) $$F_{3}\left(X_{i}\right)=\sum_{j=1}^{n}\;H_{ij},$$

where $H_{ij}$ denotes the cost of the height of the $X_{i}$ location, which is calculated by Equation (38).

(38) $$H_{ij}=\left\{\begin{array}{cc} |h_{ij}-\frac{\left(h_{max}+h_{min}\right)}{2}|, & h_{min}\leq h_{ij}\leq h_{max}\\ \infty , & otherwise \end{array}\right.,$$

where $h_{ij}$ is the altitude at which the UAV is located; $h_{min}$ is the minimum altitude at which flight is allowed; and $h_{max}$ is the maximum altitude at which flight is allowed.

4. Smoothness Costs

Moreover, due to the continuous nature of UAV flight dynamics, a drone cannot undergo abrupt changes in its motion state during flight. Therefore, path smoothness constitutes an important metric in the fitness function for UAV path planning. A smooth flight trajectory helps ensure stable maneuvering and reduces excessive control effort. Accordingly, the smoothness cost of the UAV is formulated and computed as shown in Equation (39).

(39) $$F_{4}\left(X_{i}\right)=a_{1}\sum_{j=1}^{n-2}\;\alpha_{ij}+a_{2}\sum_{j=1}^{n-1}\;\left|\beta_{ij}-\beta_{i,j-1}\right|,$$

where $a_{1}$ denotes the UAV horizontal turn angle constraint penalty coefficient, and $a_{2}$ denotes the UAV vertical pitch angle constraint penalty coefficient. $\alpha_{ij}$ denotes the horizontal turn angle constraint, which is computed by Equation (40). $\beta_{ij}$ denotes the vertical pitch angle, which is computed by Equation (41).

(40) $$\alpha_{ij}=\arctan\frac{L_{p_{ij}^{\prime }p_{i,j+1}^{\prime }}\times L_{p_{ij}^{\prime }p_{i,j+2}^{\prime }}}{L_{p_{ij}^{\prime }p_{i,j+1}^{\prime }}\cdot L_{p_{ij}^{\prime }p_{i,j+2}^{\prime }}},$$

(41) $$\beta_{ij}=\arctan\frac{Z_{i,j+1}-Z_{ij}}{∥L_{p_{ij}p_{i,j+1}}∥},$$

where $L_{p_{ij}^{\prime }p_{i,j+1}^{\prime }}$ is their projections on the plane, which is calculated by Equation (42).

(42) $$L_{p_{ij}^{\prime }p_{i,j+1}^{\prime }}=k\times \left(L_{p_{ij}p_{i,j+1}}\times k\right),$$

where k is the unit vector in the positive direction of the axis.

5. Overall Objective Function (OEF)

By jointly considering the path length cost, threat cost, altitude cost, and smoothness cost, a comprehensive multi-cost objective function is constructed to evaluate UAV flight paths. The overall objective function is defined and calculated as shown in Equation (43).

(43) $$F\left(X_{i}\right)=\sum_{k=1}^{4}\;{b_{k}*F}_{k}\left(X_{i}\right).$$

where $b_{k}$ is the weight coefficient.

#### 5.1.3. Analysis of Experimental Results

To comprehensively evaluate the performance of the proposed MICBSO, extensive experimental analyses were conducted under four representative scenarios. The detailed experimental settings and evaluation procedures are described as follows.

**Figure 9:**
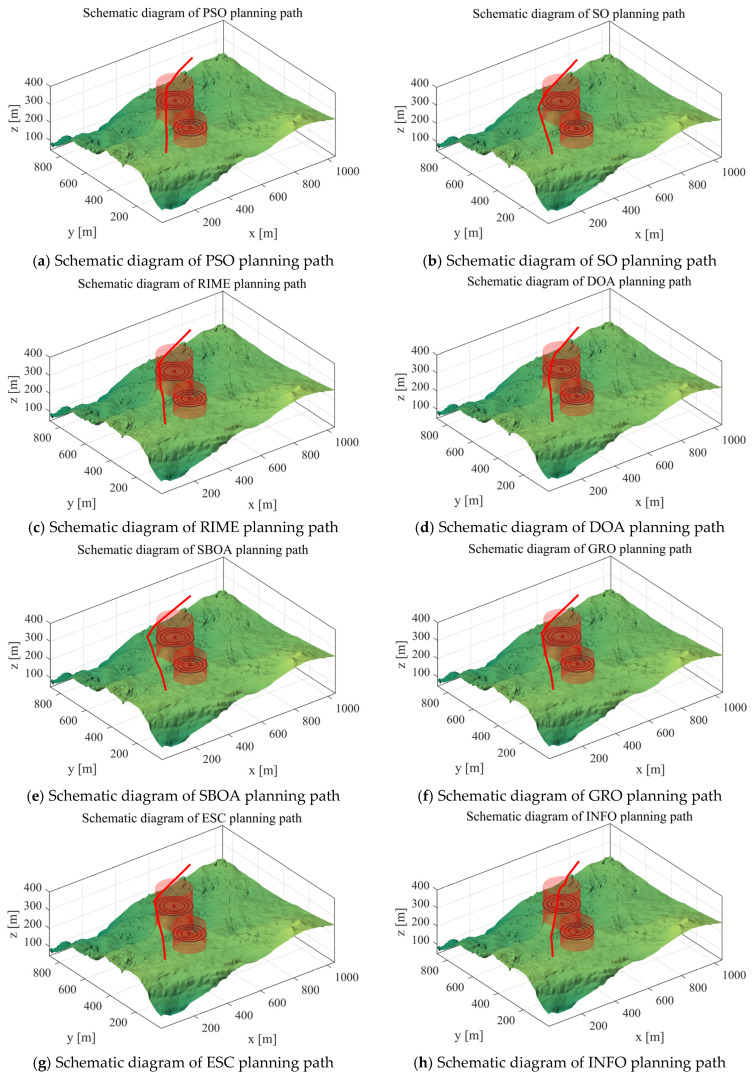

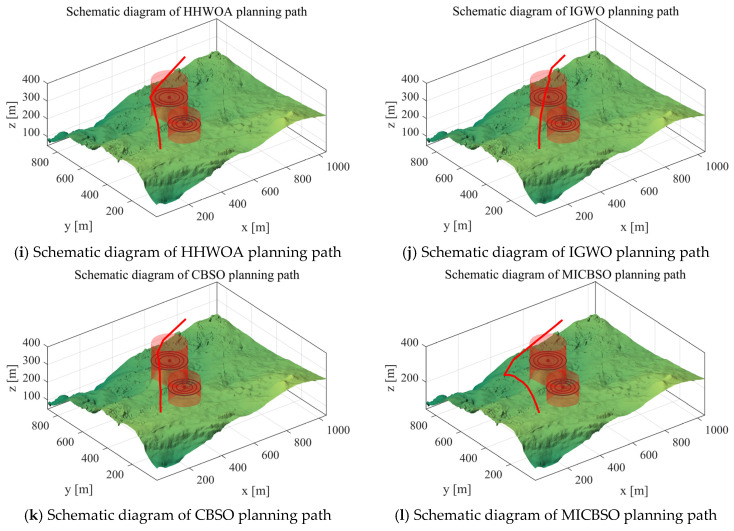
Scenario 1 Schematic diagram of path planning.

Scenario 1: In this scenario, two cylindrical obstacles are introduced to simulate real-world environmental threats, aiming to evaluate the UAV path planning performance of MICBSO under low-threat conditions. In the experimental setup, the UAV’s starting point is set to [100, 100, 150], the destination is defined as [800, 800, 150], and the number of waypoints is fixed at 10. The corresponding total path cost results are reported in Table 15, where “mean” denotes the average value obtained from 30 independent runs, “max” represents the maximum cost among these runs, and “min” indicates the best (optimal) cost achieved. The flight paths generated by each algorithm in Scenario 1 are visualized in Figure 9. The red line in the image represents the drone’s flight path.

Figure 9 shows the schematic diagram of Scenario 1 for UAV path planning. The three-dimensional environment is constructed based on a digital elevation model with cylindrical obstacles representing environmental threats. The start and target positions are located at different terrain elevations, forming a complex nonconvex search space with significant terrain variations and obstacle constraints. This scenario provides a challenging and realistic testbed for evaluating the performance of path planning algorithms.

The quantitative results obtained by different algorithms under Scenario 1 are summarized in Table 15. The evaluation metrics reflect the overall quality of the planned paths, including path cost and feasibility. As shown in Table 15, the proposed MICBSO achieves the best overall performance among all compared algorithms, producing paths with lower comprehensive costs than the other methods. This indicates that MICBSO can effectively balance path length minimization and threat avoidance in complex three-dimensional environments.

Moreover, MICBSO exhibits more stable performance across independent runs, suggesting strong robustness and reliability. By combining the visual representation in Figure 9 with the quantitative results in Table 15, it can be concluded that MICBSO is capable of generating high-quality and feasible UAV paths in realistic environments with multiple constraints.

**Figure 10:**
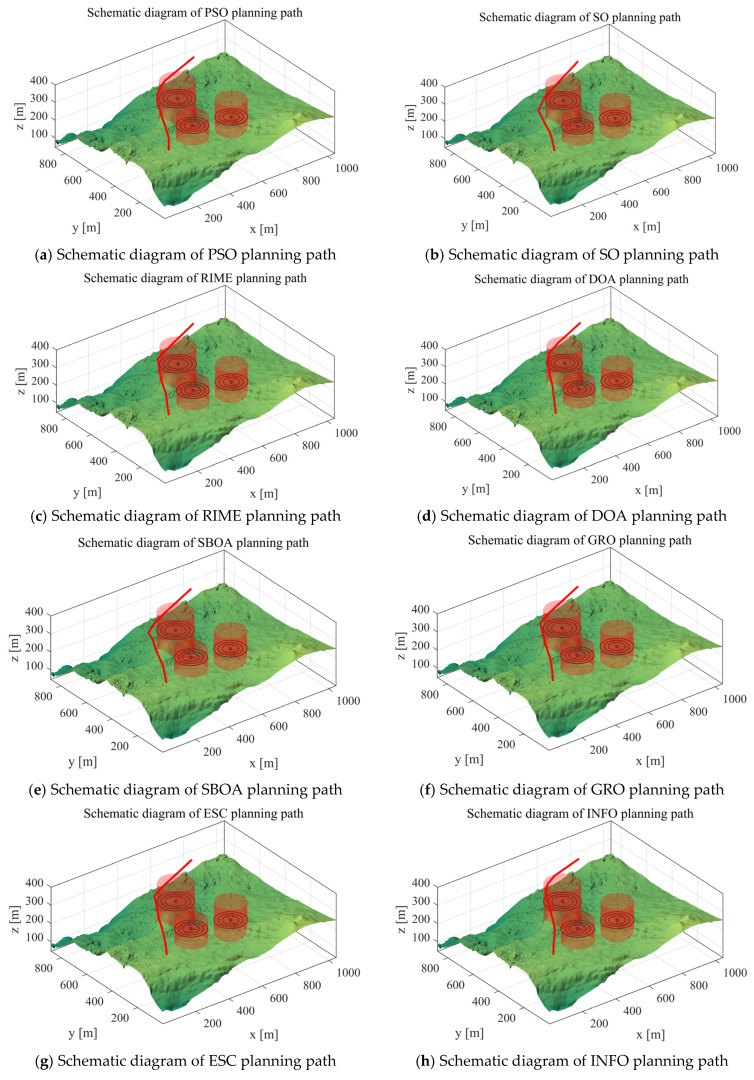

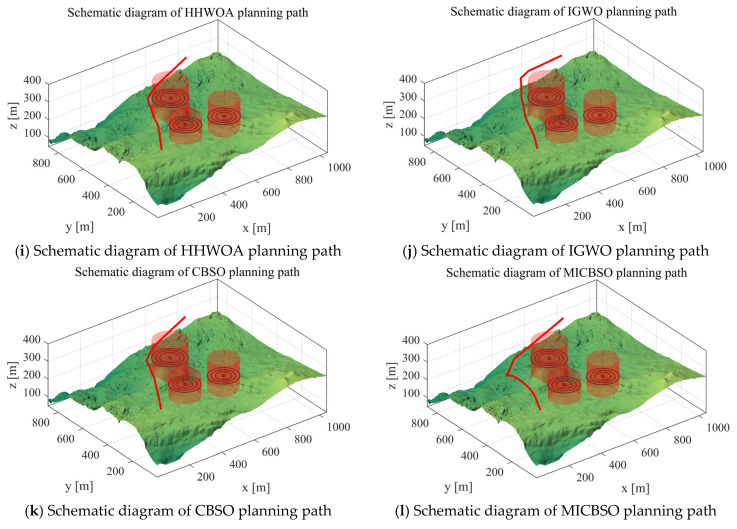
Scenario 2 Schematic diagram of path planning.

Scenario 2: In this scenario, three cylindrical obstacles are introduced to simulate real-world environmental threats, aiming to evaluate the UAV path planning performance of MICBSO under low-threat conditions. In the experimental setup, the UAV’s starting point is set to [100, 100, 150], the destination is defined as [800, 800, 150], and the number of waypoints is fixed at 10. The corresponding total path cost results are reported in Table 16, where “mean” denotes the average value obtained from 30 independent runs, “max” represents the maximum cost among these runs, and “min” indicates the best (optimal) cost achieved. The flight paths generated by each algorithm in Scenario 1 are visualized in Figure 10. The red line in the image represents the drone’s flight path.

Figure 10 presents the schematic diagram of Scenario 2 for UAV path planning. Compared with Scenario 1, this scenario features more complex terrain variations and a higher density of obstacles, resulting in a more constrained three-dimensional search space and increased planning difficulty. The experimental results of different algorithms under Scenario 2 are summarized in Table 16. The evaluation metrics reflect the overall quality and feasibility of the planned paths. As shown in Table 16, MICBSO achieves the best overall performance among the compared algorithms, producing paths with lower comprehensive costs despite the increased environmental complexity. This indicates that MICBSO can effectively adapt to more challenging environments while maintaining high-quality path planning performance. Moreover, MICBSO exhibits relatively stable results across independent runs, whereas some comparison algorithms show obvious performance degradation or increased result fluctuation. By jointly analyzing Figure 10 and Table 16, it can be concluded that MICBSO demonstrates strong adaptability and robustness in complex UAV path planning scenarios with dense constraints.

**Figure 11:**
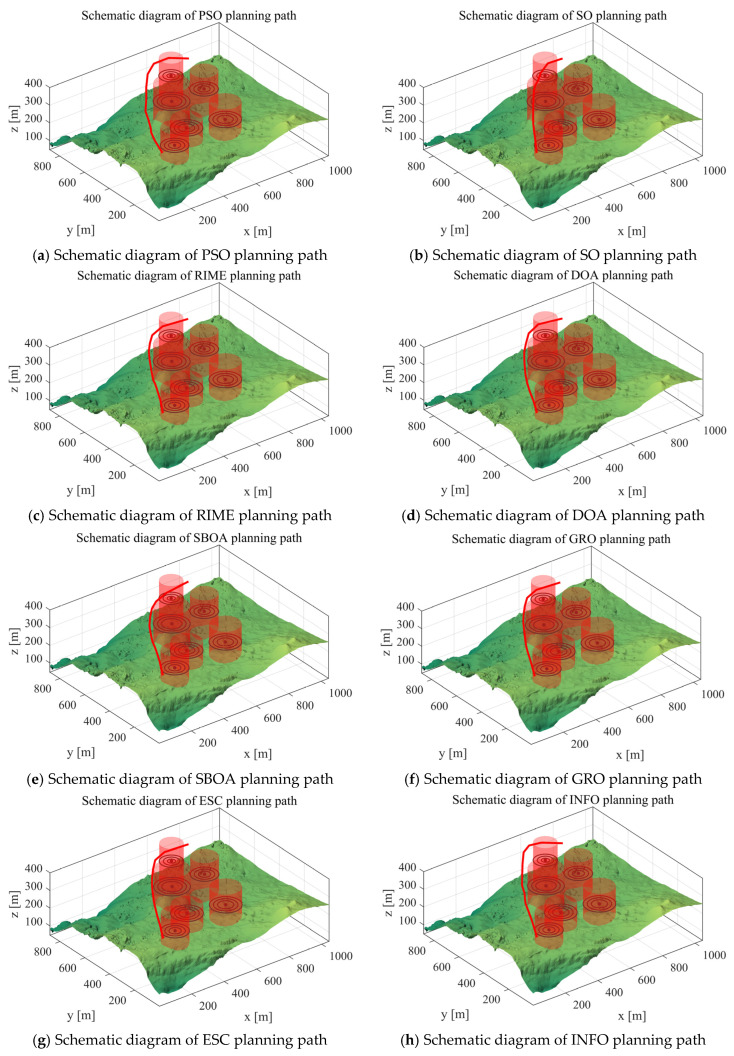

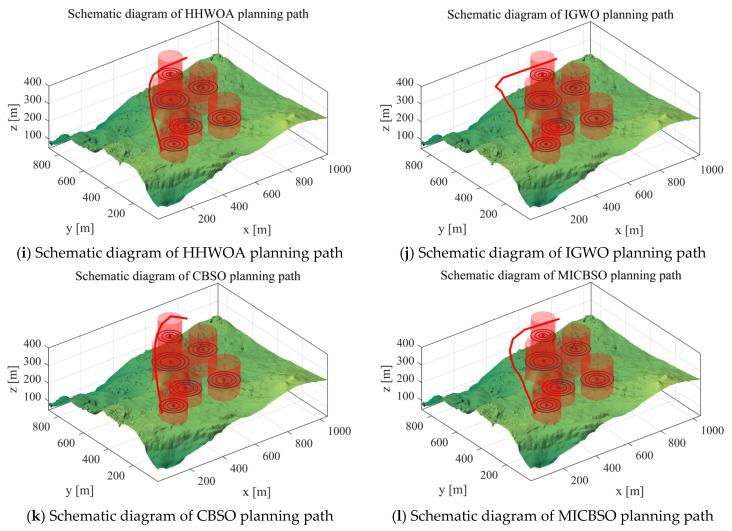
Scenario 3 Schematic diagram of path planning.

Scenario 3: In this scenario, six cylindrical obstacles are introduced to simulate real-world environmental threats, aiming to evaluate the UAV path planning performance of MICBSO under low-threat conditions. In the experimental setup, the UAV’s starting point is set to [100, 100, 150], the destination is defined as [800, 800, 150], and the number of waypoints is fixed at 10. The corresponding total path cost results are reported in Table 17, where “mean” denotes the average value obtained from 30 independent runs, “max” represents the maximum cost among these runs, and “min” indicates the best (optimal) cost achieved. The flight paths generated by each algorithm in Scenario 1 are visualized in Figure 11. The red line in the image represents the drone’s flight path.

Figure 11 illustrates the schematic diagram of Scenario 3 for UAV path planning. Compared with the previous scenarios, this environment exhibits further increased complexity in both terrain structure and obstacle distribution. The dense and irregular arrangement of environmental threats significantly narrows the feasible flight corridors, leading to a highly constrained and nonconvex three-dimensional search space. Based on the environment shown in Figure 11, the experimental results of different algorithms are summarized in Table 17. The evaluation metrics comprehensively assess the quality of the planned paths under this more challenging scenario. As indicated in Table 17, all algorithms experience an increase in path cost due to the heightened environmental difficulty; however, MICBSO still achieves the best overall performance among the compared methods. Specifically, MICBSO can generate paths with lower comprehensive costs while maintaining feasibility and safety constraints. This suggests that the proposed algorithm effectively preserves its search efficiency and solution quality even under severe terrain and obstacle constraints. In contrast, several comparison algorithms show significant performance degradation, indicating that they are more prone to getting trapped in local optima or generating unstable paths in complex environments. By jointly analyzing Figure 11 and Table 17, it can be concluded that MICBSO demonstrates strong robustness and adaptability in highly complex UAV path planning scenarios. These results further confirm the suitability of MICBSO for practical UAV applications in realistic environments with dense obstacles and multiple constraints.

**Figure 12:**
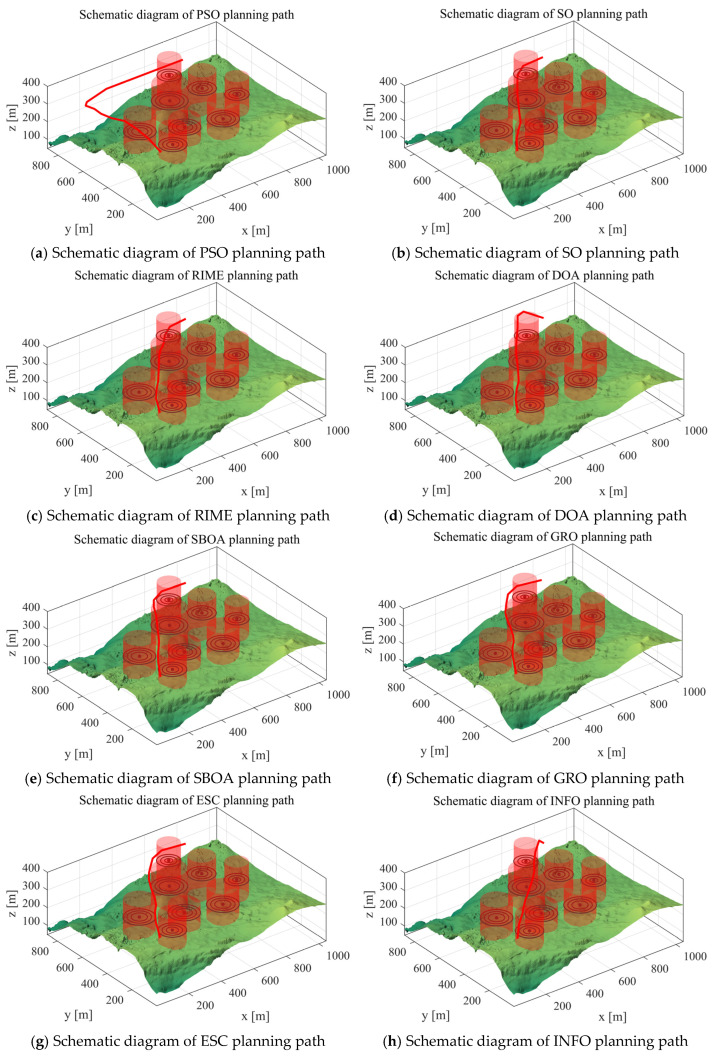

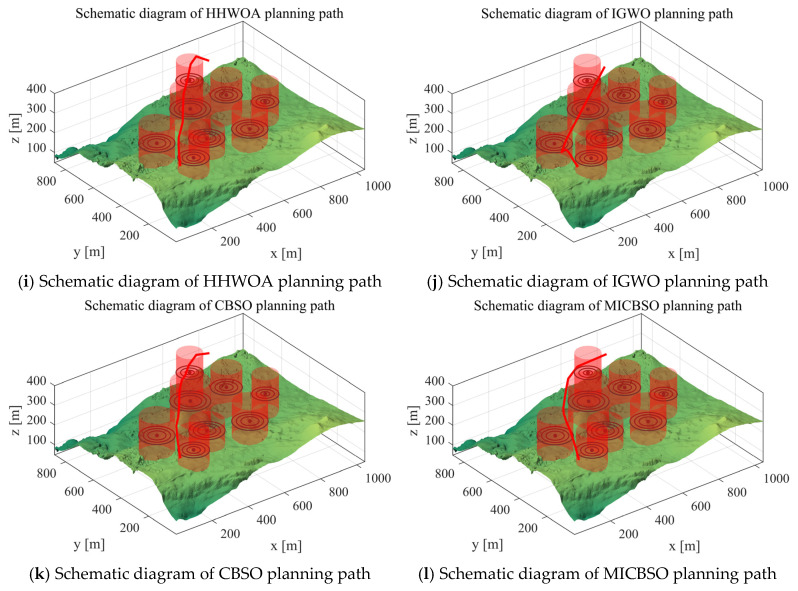
Scenario 4 Schematic diagram of path planning.

Scenario 4: In this scenario, eight cylindrical obstacles are introduced to simulate real-world environmental threats, aiming to evaluate the UAV path planning performance of MICBSO under low-threat conditions. In the experimental setup, the UAV’s starting point is set to [100, 100, 150], the destination is defined as [800, 800, 150], and the number of waypoints is fixed at 10. The corresponding total path cost results are reported in Table 18, where “mean” denotes the average value obtained from 30 independent runs, “max” represents the maximum cost among these runs, and “min” indicates the best (optimal) cost achieved. The flight paths generated by each algorithm in Scenario 1 are visualized in Figure 12. The red line in the image represents the drone’s flight path.

Figure 12 presents the schematic diagram of Scenario 4 for UAV path planning. This scenario represents the most challenging environment among all tested cases, featuring highly irregular terrain variations and densely distributed obstacles. The feasible flight space is significantly constrained, resulting in a complex three-dimensional search space with severe nonlinearity and multiple local optima. The experimental results obtained under Scenario 4 are summarized in Table 18. The evaluation metrics reflect the overall quality and feasibility of the planned paths in this highly constrained environment. As shown in Table 18, the difficulty of the scenario leads to increased path costs for all algorithms; however, MICBSO consistently achieves the best overall performance among the compared methods. Despite extreme environmental complexity, MICBSO generates paths with relatively lower comprehensive costs while maintaining feasibility and safety constraints. In contrast, several comparison algorithms exhibit significant performance degradation or increased result volatility, indicating that they are more prone to getting trapped in local optima or generating unstable solutions in highly complex scenarios. This highlights the strong global search capability and robustness of the proposed MICBSO. By jointly analyzing Figure 12 and Table 18, it can be concluded that MICBSO maintains superior adaptability and robustness even in the most complex UAV path planning scenario. These results further demonstrate the effectiveness and practical applicability of MICBSO for UAV path planning tasks in realistic three-dimensional environments with dense obstacles and severe constraints.

## 6. Conclusions

In this paper, a Multi-Strategy Improved Connected Banking System Optimizer, named MICBSO, was proposed for numerical optimization and three-dimensional UAV path planning in complex terrain. To overcome the limitations of the original CBSO in population initialization, elite guidance, and boundary handling, three improvement strategies were designed. The chaos–opposition learning initialization strategy was introduced to improve the quality and diversity of the initial population. The Gaussian perturbation-based multi-elite guidance mechanism was developed to reduce overdependence on a single best solution and enhance the balance between exploration and exploitation. The hybrid boundary control strategy was further designed to handle boundary violations by combining reflective correction and random reinitialization. In addition, theoretical analysis, time complexity analysis, exploration–exploitation analysis, population diversity analysis, ablation experiments, and parameter sensitivity analysis were conducted to provide a more comprehensive evaluation of the proposed algorithm.

The experimental results demonstrate that MICBSO has strong optimization capability and robustness. On the CEC2017 benchmark suite, MICBSO achieved competitive performance compared with 11 representative optimization algorithms under different dimensional settings. The convergence curves, boxplots, statistical tests, and ablation results further verified the effectiveness of the proposed strategies. The population diversity and exploration–exploitation analyses showed that MICBSO can maintain sufficient diversity in the early search stage and gradually strengthen exploitation in the later stage. For UAV path planning, MICBSO was tested in four complex three-dimensional terrain scenarios with different threat conditions. The simulation results showed that MICBSO can generate feasible, safe, and lower-cost flight paths, indicating its applicability to constrained path planning problems.

Although MICBSO shows promising performance, several limitations remain. First, the current UAV path planning experiments are mainly conducted in simulated three-dimensional environments, and real flight tests have not yet been performed. Real-world UAV navigation may involve more complex factors, such as dynamic obstacles, sensor noise, wind disturbance, communication delay, and unexpected environmental changes. Second, the current path planning model mainly considers static terrain and obstacle threats, while highly dynamic obstacles and real-time environmental changes are not fully investigated. Third, several parameters in MICBSO are selected based on empirical analysis, and more advanced adaptive parameter control mechanisms may further improve its robustness. Therefore, future work will focus on extending MICBSO to real-time UAV path planning in dynamic and uncertain environments, developing real-time replanning mechanisms, exploring multi-UAV cooperative planning, and validating the proposed method on physical UAV platforms. In addition, adaptive parameter adjustment and hybrid learning-based mechanisms will be further investigated to improve the search efficiency and practical applicability of the proposed algorithm.

## Figures and Tables

**Table 1:** Qualitative comparison of different chaotic maps for population initialization.

Chaotic Map	Mathematical Expression	Main Characteristics	Potential Limitation	Reason for Selection or Non-Selection in MICBSO
Logistic map [17]	$x_{t+1}=\mu x_{t}(1-x_{t}),\mu =4$	Simple structure, strong chaotic behavior, easy mapping to (0, 1), and low computational cost.	The distribution may be sensitive to the initial value.	Selected because it provides sufficient randomness and ergodicity with very low implementation cost, and can be directly combined with opposition-based learning in the initialization stage.
Tent map [18]	$\begin{array}{c} x_{t+1}=2x_{t},x_{t}<0.5;x_{t+1}=\\ 2(1-x_{t}),x_{t}\geq 0.5 \end{array}$	Simple piecewise structure and good uniformity.	Piecewise calculation is required, and the sequence may show degradation under finite numerical precision.	Not selected because its advantage over the Logistic map is limited in implementation simplicity, while the Logistic map is more commonly used and easier to integrate.
Sine map [19]	$x_{t+1}=sin(\pi x_{t})$	Nonlinear mapping and strong randomness.	The sequence distribution may be affected by the sine transformation and may be less stable for initialization.	Not selected because it may introduce stronger oscillation, which is not always beneficial for stable initial population construction.
Circle map [20]	$\begin{array}{c} x_{t+1}=\\ mod\left(x_{t}+b-\frac{a}{2\pi }sin(2\pi x_{t}),1\right) \end{array}$	Flexible chaotic behavior controlled by parameters.	Additional parameters are required, increasing parameter-tuning complexity.	Not selected because MICBSO aims to avoid adding extra control parameters during initialization.
Chebyshev map [21]	$x_{t+1}=cos(karccos(x_{t}))$	Strong nonlinear transformation and high sensitivity to initial values.	The generated sequence is usually defined in [−1, 1] and requires additional normalization before initialization.	Not selected because additional transformation is needed before mapping the sequence to the optimization search space.

**Table 2:** Comparison of Adaptive schedule Strategy.

Adaptive Schedule	Mathematical Expression	Main Characteristics	Potential Limitation	Suitability for MICBSO
Linear decay	$\eta (t)=\eta_{0}\left(1-\frac{t}{t_{max}}\right)$	Simple structure, monotonic decrease, and smooth transition from exploration to exploitation.	The attenuation rate is fixed and does not respond to search feedback.	Suitable for α(t), because multi-elite guidance should decrease smoothly to avoid excessive elite attraction.
Exponential decay	$\eta (t)=\eta_{0}exp\left[-k{\left(\frac{t}{t_{max}}\right)}^{2}\right]$	Strong early-stage influence and rapid attenuation in the middle and late stages.	The decay rate depends on k.	Suitable for β(t), because Gaussian perturbation should be strong in early exploration and weak in late exploitation.
Polynomial decay	$\eta (t)=\eta_{0}{\left(1-\frac{t}{t_{max}}\right)}^{p}$	Flexible attenuation controlled by exponent p.	Introduces an additional parameter p, increasing tuning complexity.	Not adopted to avoid introducing more empirical parameters.
Cosine decay	$\eta (t)=\frac{\eta_{0}}{2}\left(1+cos\left(\pi \frac{t}{t_{max}}\right)\right)$	Smooth nonlinear decrease with slow changes at the beginning and end.	May retain relatively large values in the middle stage and slow convergence.	Not adopted because MICBSO requires more direct control of guidance and perturbation reduction.
Sigmoid decay	$\eta (t)=\frac{\eta_{0}}{1+exp\left(a\left(\frac{t}{t_{max}}-b\right)\right)}$	Can realize a slow-fast-slow adaptive transition.	Requires additional parameters a and b.	Not adopted because it increases parameter-tuning burden.
Feedback-based adaptive schedule	η(t) is adjusted according to fitness improvement or population diversity.	Can dynamically respond to the search state.	Requires extra monitoring indicators and increases implementation complexity.	Considered as a promising future extension, but not adopted in the current MICBSO to keep the mechanism simple and efficient.

**Table 3:** Comparison of different boundary-handling strategies.

Boundary-Handling Strategy	Mathematical Form	Main Advantage	Potential Limitation	Difference from the Proposed MICBSO Strategy
Saturation/Clipping [23]	$X_{ij}=min(max(X_{ij},lb_{j}),ub_{j})$	Simple implementation and guarantees feasibility.	Out-of-bounds individuals are directly forced to the boundary, which may cause population aggregation near the boundary and reduce diversity.	MICBSO does not simply clip infeasible solutions to the boundary. It uses reflection or random reinitialization according to the violation degree, which helps preserve search direction and diversity.
Periodic mapping [24]	$\begin{array}{c} X_{ij}=lb_{j}+mod(X_{ij}-\\ \left.lb_{j},ub_{j}-lb_{j}\right) \end{array}$	Can map infeasible solutions back into the feasible region and maintain search continuity.	The mapped position may be far from the original search direction, and the physical meaning of periodicity may not be suitable for all optimization problems.	MICBSO avoids periodic wrapping because UAV path planning and benchmark optimization usually do not have periodic boundaries.
Random repair [25]	$X_{ij}=lb_{j}+rand(ub_{j}-lb_{j})$	Improves diversity and avoids boundary stagnation.	Useful search information may be destroyed if all violations are randomly reset, which may slow convergence.	MICBSO only applies random repair to severe violations. Minor violations are handled by reflection to retain useful search information.
Pure reflection [26]	$\begin{array}{c} X_{ij}=2ub_{j}-X_{ij}\;if\;X_{ij}>ub_{j};\\ X_{ij}=2lb_{j}-X_{ij}\;if\;X_{ij}<lb_{j} \end{array}$	Preserves part of the original search direction and improves search continuity.	For large violations, a single reflection may still produce infeasible solutions, while repeated reflection may cause boundary oscillation.	MICBSO applies reflection only to minor violations. Severe violations are randomly reinitialized to avoid repeated reflection and boundary oscillation.
Proposed hybrid boundary control	Reflection is used when the violation is smaller than δ; otherwise, random reinitialization is adopted.	Balances search continuity and population diversity; avoids both boundary aggregation and excessive random resetting.	Requires setting the threshold δ.	MICBSO combines the advantages of reflection and random repair. It preserves useful search direction for minor violations and introduces diversity for severe violations.

**Table 4:** Computational complexity comparison between CBSO and MICBSO.

Algorithm	Initialization Cost	Iterative Update Cost	Main Additional Operations	Overall Complexity
CBSO	$O(ND+NC_{f})$	$O(T(ND+NC_{f}))$	None	$O(T(ND+NC_{f}))$
MICBSO	$O(2ND+2NC_{f})$	$\begin{array}{c} O(T(ND+NC_{f}+\\ \left.NlogN+KD)\right) \end{array}$	Opposition-based learning, elite selection, Gaussian perturbation, and boundary control	$O(T(ND+NC_{f}+NlogN))$

**Table 5:** Comparison of average execution time of different algorithms.

Algorithm/Runtime (s)	Dim = 30	Dim = 50	Dim = 100
PSO	14.72	28.76	85.45
SO	16.27	29.70	85.92
RIME	17.00	30.95	88.35
DOA	20.02	38.93	119.04
SBOA	26.58	50.65	152.92
GRO	32.65	35.60	88.67
ESC	79.55	99.58	171.10
INFO	27.79	60.53	101.66
HHWOA	211.38	409.51	1250.40
IGWO	61.08	93.06	214.92
CBSO	22.60	43.49	150.38
MICBSO	23.94	48.33	172.33

**Table 6:** Parameter settings of the comparison algorithms.

Algorithms	Parameter Name	Parameter Value	Reference
PSO	Vmax, wMax, wMin, c1, c2	6, 0.9, 0.6, 2, 2	[28]
SO	c1, c2, c3	0.5, 0.5, 2	[29]
RIME	W	5	[30]
DOA	PC	15	[31]
SBOA	u	0.5	[32]
GRO	sigma_initial	2	[33]
ESC	eliteSize, beta_base	5, 1.5	[34]
INFO	e	1 × 10^−25^	[35]
HHWOA	w	3	[36]
IGWO	at	2	[37]
CBSO	beta	1.5	[14]

**Table 7:** Results of various algorithms tested on the CEC 2017 benchmark (dim = 30).

ID	Metric	PSO	SO	RIME	DOA	SBOA	GRO	ESC	INFO	HHWOA	IGWO	CBSO	MICBSO
F1	mean	2.2445E+05	4.5024E+06	4.2545E+05	6.5161E+03	5.1934E+03	1.5116E+06	4.3093E+03	1.8815E+02	2.8634E+03	3.6026E+05	2.7357E+06	2.0201E+02
	std	1.1018E+05	8.4897E+06	1.5928E+05	6.4955E+03	6.4388E+03	3.5924E+06	3.7369E+03	2.8680E+02	3.2628E+03	2.0191E+05	2.7949E+06	7.8949E+01
F2	mean	6.1304E+09	4.0824E+22	6.6657E+12	7.7421E+15	8.0268E+12	3.6012E+23	1.7556E+18	3.0388E+20	2.0724E+17	3.9001E+16	7.6376E+16	2.2570E+07
	std	1.3826E+10	1.2605E+23	1.1027E+13	4.2397E+16	1.3238E+13	1.4463E+24	9.6132E+18	1.6640E+21	8.4806E+17	1.1377E+17	3.4500E+17	2.3085E+07
F3	mean	5.3563E+03	5.3338E+04	5.5154E+03	3.5936E+04	6.2990E+03	3.5854E+04	4.4835E+04	9.1783E+02	3.0000E+02	5.5913E+03	8.5585E+02	4.6485E+04
	std	2.5030E+03	1.0667E+04	3.1565E+03	1.6391E+04	2.5648E+03	7.4730E+03	1.0526E+04	1.2275E+03	3.0069E−03	2.6216E+03	5.5166E+02	5.7464E+03
F4	mean	4.6682E+02	4.9760E+02	5.0956E+02	4.8575E+02	4.9505E+02	5.0906E+02	5.0170E+02	4.7338E+02	4.7161E+02	4.9444E+02	4.9921E+02	4.7040E+02
	std	2.7945E+01	8.3996E+00	3.0225E+01	2.0271E+01	2.6449E+01	2.5434E+01	1.6549E+01	3.0972E+01	3.0802E+01	1.4348E+01	1.3563E+01	1.6590E+01
F5	mean	6.7023E+02	5.7610E+02	5.8464E+02	6.9289E+02	5.6363E+02	5.7444E+02	5.7578E+02	6.4602E+02	5.8700E+02	5.7077E+02	5.9061E+02	5.3209E+02
	std	3.4040E+01	4.6332E+01	2.0844E+01	5.2110E+01	1.8322E+01	1.7595E+01	1.7924E+01	3.9557E+01	2.7269E+01	3.9051E+01	2.6411E+01	3.9701E+00
F6	mean	6.4249E+02	6.0215E+02	6.0406E+02	6.2770E+02	6.0065E+02	6.0443E+02	6.0000E+02	6.2264E+02	6.0403E+02	6.0038E+02	6.1211E+02	6.0000E+02
	std	6.6906E+00	7.5533E−01	2.0413E+00	1.4512E+01	1.2217E+00	1.8510E+00	3.7794E−03	9.8947E+00	2.4216E+00	1.9310E−01	4.2418E+00	7.9266E−06
F7	mean	8.5400E+02	7.8215E+02	8.1807E+02	1.0575E+03	8.0608E+02	8.0233E+02	8.1970E+02	9.4449E+02	8.6462E+02	8.3866E+02	8.2625E+02	7.6193E+02
	std	3.4736E+01	1.4279E+01	2.1570E+01	1.0161E+02	2.7693E+01	1.9755E+01	1.5248E+01	4.9385E+01	3.6127E+01	6.6612E+01	2.5734E+01	4.1615E+00
F8	mean	9.1101E+02	8.7190E+02	8.7921E+02	9.4635E+02	8.6133E+02	8.6771E+02	8.7254E+02	9.3094E+02	8.8412E+02	8.6289E+02	8.8281E+02	8.3472E+02
	std	2.1333E+01	3.6456E+01	1.8974E+01	5.2320E+01	1.3717E+01	1.4590E+01	2.0568E+01	2.5578E+01	2.6392E+01	3.6470E+01	1.9880E+01	4.7312E+00
F9	mean	4.5545E+03	9.5783E+02	1.4533E+03	2.8577E+03	9.8767E+02	1.1812E+03	9.0054E+02	2.4738E+03	1.3795E+03	9.2615E+02	1.5701E+03	9.0020E+02
	std	1.2445E+03	4.4378E+01	5.4215E+02	1.4705E+03	2.1186E+02	2.1805E+02	8.5218E−01	5.4900E+02	4.0196E+02	1.1784E+02	4.4562E+02	1.8447E−01
F10	mean	4.6302E+03	6.6582E+03	4.3025E+03	7.6654E+03	4.0712E+03	4.1712E+03	6.5190E+03	5.0205E+03	4.7149E+03	6.6319E+03	4.5158E+03	2.9157E+03
	std	5.4621E+02	1.3492E+03	6.2116E+02	6.7173E+02	7.2153E+02	6.0855E+02	4.5172E+02	8.4464E+02	9.0174E+02	1.9008E+03	5.6526E+02	1.6920E+02
F11	mean	1.2159E+03	1.2784E+03	1.2899E+03	1.2715E+03	1.1782E+03	1.2304E+03	1.1753E+03	1.2398E+03	1.2015E+03	1.1724E+03	1.2171E+03	1.1197E+03
	std	3.5659E+01	6.3421E+01	3.6225E+01	6.0245E+01	3.4519E+01	3.6705E+01	2.9753E+01	4.9913E+01	4.5882E+01	2.9683E+01	3.0368E+01	4.4640E+00
F12	mean	1.2846E+06	5.6551E+05	8.3256E+06	2.3123E+05	3.7671E+05	9.4387E+05	8.7272E+05	6.7619E+04	4.4696E+04	1.6247E+06	1.5054E+04	5.9746E+04
	std	1.0991E+06	6.5613E+05	6.3952E+06	3.0626E+05	3.3103E+05	6.6262E+05	5.9014E+05	6.3904E+04	3.5333E+04	9.3389E+05	8.0077E+03	2.1423E+04
F13	mean	1.5704E+04	3.2165E+05	5.0494E+04	1.9412E+04	2.0097E+04	2.3680E+04	1.4957E+04	1.8311E+04	1.6779E+04	1.1047E+05	2.5926E+03	2.1658E+03
	std	1.4348E+04	1.1281E+06	3.2957E+04	1.9272E+04	1.8804E+04	1.4207E+04	1.2741E+04	1.7568E+04	1.9350E+04	5.9541E+04	5.9769E+02	7.0380E+02
F14	mean	1.7193E+04	2.3853E+04	3.4218E+04	7.2917E+03	1.4564E+04	1.0237E+04	8.5669E+04	2.3562E+03	1.4789E+03	9.1363E+03	1.4801E+03	4.3178E+03
	std	1.3567E+04	2.1176E+04	3.0942E+04	4.8910E+03	1.8608E+04	9.7605E+03	1.2708E+05	1.2611E+03	2.7485E+01	6.5199E+03	1.2351E+01	1.6715E+03
F15	mean	6.4899E+03	1.5596E+04	1.2765E+04	1.1066E+04	1.0341E+04	7.7608E+03	6.7266E+03	1.0360E+04	4.9003E+03	2.5799E+04	1.7029E+03	1.5971E+03
	std	7.5133E+03	1.6300E+04	1.1383E+04	1.1660E+04	8.8361E+03	5.2031E+03	6.6256E+03	1.0890E+04	1.0595E+04	2.2402E+04	6.6731E+01	6.6473E+01
F16	mean	2.6481E+03	2.4988E+03	2.5516E+03	2.7941E+03	2.1175E+03	2.1310E+03	2.0927E+03	2.6922E+03	2.5236E+03	2.1671E+03	2.3074E+03	1.9276E+03
	std	2.8172E+02	3.6653E+02	2.9794E+02	3.1518E+02	2.1690E+02	2.2685E+02	1.7137E+02	2.7406E+02	3.2934E+02	4.4609E+02	2.4162E+02	1.3300E+02
F17	mean	2.3366E+03	1.9192E+03	2.0842E+03	2.2191E+03	1.8628E+03	1.8298E+03	1.8124E+03	2.2132E+03	2.1724E+03	1.8380E+03	1.9431E+03	1.7537E+03
	std	2.4853E+02	1.1531E+02	1.7278E+02	1.7258E+02	8.0754E+01	7.3421E+01	8.2278E+01	2.0173E+02	2.2599E+02	1.1819E+02	1.1479E+02	1.3145E+01
F18	mean	3.2829E+05	4.3929E+05	5.3792E+05	1.0801E+05	3.3899E+05	2.4865E+05	5.1162E+05	3.9057E+04	5.5929E+03	2.2061E+05	1.9335E+03	6.8157E+04
	std	2.9776E+05	4.4473E+05	3.0906E+05	8.6927E+04	2.8587E+05	2.0226E+05	5.5885E+05	2.4607E+04	5.1324E+03	1.6789E+05	8.1376E+01	2.3139E+04
F19	mean	8.1613E+03	1.5874E+04	1.3011E+04	1.0901E+04	1.3715E+04	6.0572E+03	1.1384E+04	3.7665E+03	3.8095E+03	1.2606E+04	1.9562E+03	2.0992E+03
	std	6.7568E+03	1.9121E+04	1.4066E+04	1.1555E+04	1.1827E+04	4.5824E+03	9.4733E+03	4.2308E+03	9.9081E+03	1.1208E+04	1.1444E+01	1.4591E+02
F20	mean	2.6146E+03	2.2522E+03	2.3796E+03	2.5762E+03	2.1934E+03	2.2530E+03	2.1775E+03	2.5973E+03	2.4726E+03	2.2442E+03	2.3491E+03	2.1332E+03
	std	1.5888E+02	1.7199E+02	2.2115E+02	1.7536E+02	1.2639E+02	7.1708E+01	9.4811E+01	2.3063E+02	1.7739E+02	1.9150E+02	1.0173E+02	4.0792E+01
F21	mean	2.4781E+03	2.3837E+03	2.3892E+03	2.4361E+03	2.3464E+03	2.3550E+03	2.3777E+03	2.4185E+03	2.3748E+03	2.3665E+03	2.3840E+03	2.3315E+03
	std	4.7864E+01	3.5896E+01	2.3844E+01	4.4595E+01	1.1291E+01	1.2414E+01	1.6519E+01	3.0279E+01	2.2072E+01	4.3168E+01	1.7279E+01	4.5328E+00
F22	mean	4.6084E+03	4.3714E+03	4.1636E+03	4.4441E+03	2.3005E+03	2.3119E+03	3.5146E+03	5.0740E+03	4.2709E+03	3.3703E+03	2.5789E+03	2.3000E+03
	std	2.1101E+03	2.8096E+03	1.7931E+03	3.0934E+03	1.0556E+00	1.6275E+01	2.2570E+03	2.2095E+03	2.1974E+03	2.2419E+03	9.6813E+02	1.2620E−08
F23	mean	3.0915E+03	2.7245E+03	2.7565E+03	2.8278E+03	2.6962E+03	2.7149E+03	2.7048E+03	2.8328E+03	2.7597E+03	2.7106E+03	2.7526E+03	2.6834E+03
	std	1.3550E+02	2.6805E+01	2.4592E+01	6.7261E+01	1.8243E+01	1.9525E+01	2.5124E+01	4.1968E+01	4.2667E+01	4.2628E+01	2.5092E+01	5.9522E+00
F24	mean	3.1724E+03	2.9486E+03	2.9251E+03	2.9910E+03	2.8634E+03	2.8781E+03	2.9182E+03	2.9834E+03	2.9166E+03	2.8765E+03	2.9185E+03	2.8539E+03
	std	7.5108E+01	4.5929E+01	2.6986E+01	7.1573E+01	1.4772E+01	1.9206E+01	1.0649E+01	5.0238E+01	2.6552E+01	4.3941E+01	2.9438E+01	4.5210E+00
F25	mean	2.8854E+03	2.8941E+03	2.8946E+03	2.8890E+03	2.8968E+03	2.9083E+03	2.8885E+03	2.8944E+03	2.9047E+03	2.8874E+03	2.8949E+03	2.8843E+03
	std	1.4559E+01	5.1656E+00	1.1056E+01	1.0489E+01	1.8408E+01	1.9252E+01	1.1020E+00	1.4077E+01	2.0809E+01	1.7972E+00	8.8850E+00	1.4194E+00
F26	mean	5.7727E+03	4.1922E+03	4.6241E+03	5.3358E+03	4.0075E+03	3.7930E+03	3.9838E+03	5.6628E+03	4.9525E+03	3.9942E+03	4.2553E+03	3.6256E+03
	std	1.9794E+03	1.9300E+02	5.5500E+02	1.2054E+03	5.3479E+02	6.8329E+02	1.7276E+02	1.0068E+03	7.1843E+02	5.2408E+02	6.1960E+02	5.0760E+02
F27	mean	3.2912E+03	3.2192E+03	3.2296E+03	3.2620E+03	3.2124E+03	3.2361E+03	3.2179E+03	3.2525E+03	3.2490E+03	3.2008E+03	3.2281E+03	3.2046E+03
	std	1.7159E+02	6.4844E+00	1.2673E+01	5.6803E+01	1.2106E+01	1.6062E+01	1.0567E+01	3.1434E+01	2.6938E+01	8.9998E+00	1.1852E+01	5.6429E+00
F28	mean	3.2239E+03	3.2620E+03	3.2640E+03	3.2112E+03	3.2136E+03	3.2712E+03	3.2241E+03	3.2043E+03	3.1749E+03	3.2204E+03	3.2356E+03	3.1641E+03
	std	3.2583E+01	1.6851E+01	3.6890E+01	3.2494E+01	1.8171E+01	2.8704E+01	1.7411E+01	3.3318E+01	5.9471E+01	9.8525E+00	2.0049E+01	3.5191E+01
F29	mean	3.9890E+03	3.6351E+03	3.8745E+03	3.9041E+03	3.5113E+03	3.6382E+03	3.4456E+03	4.0097E+03	4.0210E+03	3.4714E+03	3.7635E+03	3.4292E+03
	std	2.4187E+02	1.5541E+02	2.1408E+02	2.2199E+02	1.4313E+02	1.1655E+02	7.3570E+01	2.5473E+02	2.4599E+02	1.3666E+02	1.8355E+02	5.2060E+01
F30	mean	3.8900E+04	2.9427E+04	2.2741E+05	1.4100E+04	1.5340E+04	1.2967E+04	1.0706E+04	9.5397E+03	9.7848E+03	1.5429E+05	9.3416E+03	5.6321E+03
	std	2.8477E+04	5.3972E+04	1.9396E+05	6.3801E+03	7.8800E+03	4.3355E+03	3.6815E+03	3.7205E+03	3.7585E+03	8.3793E+04	2.9398E+03	3.1044E+02

**Table 8:** Results of various algorithms tested on the CEC 2017 benchmark (dim = 50).

ID	Metric	PSO	SO	RIME	DOA	SBOA	GRO	ESC	INFO	HHWOA	IGWO	CBSO	MICBSO
F1	mean	9.3168E+06	1.0989E+08	4.3375E+06	5.9364E+05	1.4676E+04	5.6209E+08	3.8961E+03	6.2164E+03	2.9300E+03	1.4707E+07	3.0718E+08	2.4889E+02
	std	2.8133E+06	6.2461E+07	1.6518E+06	3.0938E+06	1.1875E+04	4.6670E+08	3.6324E+03	7.8336E+03	3.8784E+03	8.3585E+06	1.8394E+08	7.5131E+01
F2	mean	4.7032E+22	1.4122E+50	2.6137E+31	6.4647E+32	9.6994E+34	2.5197E+47	6.7198E+43	6.8875E+43	1.4557E+47	1.1070E+38	3.0605E+42	3.2794E+28
	std	1.2040E+23	4.2588E+50	8.7321E+31	2.6791E+33	5.3000E+35	7.4210E+47	3.4869E+44	3.6974E+44	5.4731E+47	4.8480E+38	1.5876E+43	5.0593E+28
F3	mean	7.5980E+04	1.3845E+05	8.1194E+04	1.4532E+05	3.9953E+04	1.0580E+05	1.6878E+05	2.6752E+04	1.1240E+03	3.3158E+04	2.6915E+04	1.6717E+05
	std	1.4808E+04	1.3357E+04	2.4567E+04	4.5739E+04	1.0631E+04	1.2920E+04	3.2208E+04	6.6863E+03	8.2458E+02	1.0645E+04	8.6768E+03	1.2995E+04
F4	mean	5.4804E+02	6.2986E+02	6.2517E+02	5.5154E+02	5.4971E+02	7.2682E+02	5.9329E+02	5.4306E+02	5.1695E+02	5.9121E+02	6.5157E+02	4.7076E+02
	std	6.2897E+01	1.5959E+01	5.3761E+01	5.5856E+01	5.5131E+01	7.6739E+01	4.5052E+01	5.6373E+01	5.1169E+01	4.5456E+01	3.6925E+01	2.4022E+01
F5	mean	7.7958E+02	6.7753E+02	6.8133E+02	8.5215E+02	6.6124E+02	7.0617E+02	6.7092E+02	7.9434E+02	7.0489E+02	6.4575E+02	6.8939E+02	5.8402E+02
	std	3.0702E+01	6.9064E+01	3.9589E+01	6.7504E+01	4.2323E+01	3.1203E+01	4.9089E+01	4.6182E+01	4.8948E+01	4.8397E+01	3.8500E+01	7.5091E+00
F6	mean	6.5171E+02	6.0465E+02	6.1491E+02	6.5654E+02	6.0512E+02	6.1557E+02	6.0010E+02	6.3844E+02	6.1824E+02	6.0211E+02	6.2632E+02	6.0010E+02
	std	4.9232E+00	8.2934E−01	4.7957E+00	1.2395E+01	3.3681E+00	4.2104E+00	1.4861E−01	8.6125E+00	8.3615E+00	1.3846E+00	4.8099E+00	2.3780E−02
F7	mean	1.1020E+03	8.8434E+02	9.7929E+02	1.5522E+03	9.8882E+02	1.0040E+03	9.7144E+02	1.3280E+03	1.1377E+03	9.5442E+02	1.0127E+03	1.0733E+03
	std	7.4691E+01	3.1602E+01	5.4391E+01	1.2773E+02	7.1519E+01	4.7111E+01	4.0614E+01	9.8033E+01	9.1083E+01	9.6775E+01	4.3340E+01	4.1927E+01
F8	mean	1.0993E+03	9.7522E+02	9.9520E+02	1.1673E+03	9.6439E+02	1.0067E+03	9.8069E+02	1.1019E+03	1.0067E+03	9.5489E+02	1.0118E+03	8.8294E+02
	std	2.9457E+01	5.2345E+01	4.1897E+01	7.5132E+01	2.9604E+01	3.5462E+01	4.7774E+01	4.9265E+01	3.6589E+01	7.6501E+01	2.8108E+01	7.7060E+00
F9	mean	2.0326E+04	1.1528E+03	5.0793E+03	1.8595E+04	2.8642E+03	4.0067E+03	9.5151E+02	7.9593E+03	3.0970E+03	1.4627E+03	4.7432E+03	9.0513E+02
	std	4.8307E+03	7.1778E+01	2.3904E+03	6.5172E+03	1.1846E+03	1.6876E+03	7.2691E+01	1.5674E+03	1.4786E+03	5.1219E+02	1.1131E+03	2.3718E+00
F10	mean	7.5555E+03	1.3549E+04	7.3655E+03	1.3933E+04	6.7644E+03	7.3112E+03	1.2162E+04	8.2181E+03	7.8804E+03	1.0847E+04	7.9532E+03	5.1953E+03
	std	9.4247E+02	1.4598E+03	8.0844E+02	1.0639E+03	1.1232E+03	6.6391E+02	7.2948E+02	8.7932E+02	8.6056E+02	3.9048E+03	7.8449E+02	3.4328E+02
F11	mean	1.3035E+03	1.6478E+03	1.5766E+03	1.3640E+03	1.2589E+03	1.9085E+03	1.4818E+03	1.3237E+03	1.3526E+03	1.4405E+03	1.4066E+03	1.2569E+03
	std	4.5375E+01	1.4570E+02	1.1423E+02	8.1431E+01	5.2703E+01	4.2123E+02	4.1330E+02	6.6385E+01	5.7777E+01	8.1687E+01	5.1989E+01	3.2104E+01
F12	mean	1.1801E+07	1.2070E+07	9.2433E+07	3.1136E+06	4.0208E+06	1.2072E+07	5.1967E+06	1.8915E+06	8.7377E+05	2.4278E+07	1.0773E+07	8.3315E+05
	std	7.3731E+06	8.6002E+06	4.9167E+07	2.1107E+06	2.2684E+06	7.0701E+06	2.9306E+06	1.5715E+06	7.0655E+05	1.0313E+07	6.2090E+06	2.3467E+05
F13	mean	2.9464E+04	4.8504E+04	1.9100E+05	1.5080E+04	1.0892E+04	9.5988E+03	8.9203E+03	8.7026E+03	1.0480E+04	3.3180E+05	1.7198E+04	1.6020E+03
	std	1.2775E+04	3.0634E+04	9.4552E+04	1.1403E+04	1.0698E+04	7.0050E+03	3.5820E+03	5.3515E+03	8.6133E+03	1.8741E+05	6.2326E+03	1.3346E+02
F14	mean	1.5699E+05	2.3429E+05	2.1240E+05	8.8373E+04	1.3506E+05	1.3694E+05	4.8380E+05	1.7882E+04	8.6129E+03	7.6413E+04	1.6178E+03	1.9137E+04
	std	1.4217E+05	2.8972E+05	1.5071E+05	7.0705E+04	1.0803E+05	9.3117E+04	6.1969E+05	1.7438E+04	8.4316E+03	6.7134E+04	2.8098E+01	5.0947E+03
F15	mean	8.1432E+03	1.8082E+04	5.1507E+04	8.3700E+03	1.2479E+04	8.8785E+03	7.4238E+03	1.2766E+04	9.8761E+03	7.8023E+04	2.6048E+03	2.4371E+03
	std	8.1207E+03	1.2325E+04	2.3178E+04	8.2223E+03	6.3221E+03	5.3877E+03	4.2686E+03	7.8441E+03	8.0813E+03	5.4955E+04	3.3700E+02	5.9122E+02
F16	mean	3.3335E+03	3.8450E+03	3.5548E+03	3.8947E+03	2.8000E+03	2.7605E+03	3.1736E+03	3.3855E+03	3.2405E+03	2.5293E+03	3.2423E+03	2.4030E+03
	std	4.1071E+02	7.3937E+02	4.3838E+02	5.6887E+02	3.5827E+02	3.6238E+02	2.9417E+02	4.5063E+02	4.4851E+02	3.2463E+02	3.7151E+02	2.1212E+02
F17	mean	3.1323E+03	3.3760E+03	3.3367E+03	3.4587E+03	2.5898E+03	2.7194E+03	2.5987E+03	3.4201E+03	3.1532E+03	2.5623E+03	2.9792E+03	2.2312E+03
	std	3.0157E+02	3.6395E+02	2.4362E+02	4.2374E+02	2.2983E+02	2.2640E+02	2.3768E+02	2.9090E+02	2.7376E+02	4.7610E+02	2.8068E+02	1.3655E+02
F18	mean	1.3660E+06	2.8554E+06	2.9141E+06	4.2511E+05	1.4518E+06	1.4493E+06	4.6443E+06	1.3633E+05	3.7610E+04	8.1260E+05	5.0636E+03	3.1040E+05
	std	8.5911E+05	2.3447E+06	2.3020E+06	3.3207E+05	9.1363E+05	7.6051E+05	4.3066E+06	1.0273E+05	3.1745E+04	6.7318E+05	5.7293E+03	1.0379E+05
F19	mean	1.5215E+04	2.0145E+04	4.4548E+04	1.6036E+04	1.4131E+04	1.5329E+04	1.6309E+04	1.7864E+04	1.6139E+04	5.4519E+04	2.2395E+03	5.3721E+03
	std	1.0809E+04	1.0541E+04	2.6534E+04	1.4160E+04	1.2445E+04	8.9955E+03	9.8522E+03	1.1074E+04	1.0169E+04	2.2523E+04	5.0166E+02	1.9634E+03
F20	mean	3.2502E+03	3.5249E+03	3.1282E+03	3.5495E+03	2.7022E+03	2.6988E+03	2.8123E+03	3.2077E+03	3.0669E+03	2.8101E+03	3.0326E+03	2.3393E+03
	std	2.9764E+02	2.8243E+02	2.8854E+02	4.0995E+02	2.5285E+02	1.9038E+02	2.6339E+02	3.6036E+02	2.8403E+02	5.0285E+02	2.7672E+02	1.0322E+02
F21	mean	2.6614E+03	2.4818E+03	2.4921E+03	2.6786E+03	2.4323E+03	2.4632E+03	2.4776E+03	2.5693E+03	2.4992E+03	2.4361E+03	2.5017E+03	2.3816E+03
	std	4.7769E+01	6.1836E+01	3.5895E+01	1.1377E+02	2.3776E+01	2.8163E+01	4.7074E+01	5.6407E+01	3.8346E+01	5.1219E+01	3.1607E+01	9.9537E+00
F22	mean	9.4830E+03	1.4479E+04	8.9782E+03	1.4997E+04	7.4659E+03	5.7576E+03	1.3602E+04	9.7967E+03	9.5515E+03	1.2500E+04	9.6200E+03	4.2647E+03
	std	1.0049E+03	1.3364E+03	9.6793E+02	3.0300E+03	2.8185E+03	3.4279E+03	7.3888E+02	1.2709E+03	9.5100E+02	3.9898E+03	7.3099E+02	2.0074E+03
F23	mean	3.6493E+03	2.8676E+03	2.9588E+03	3.1859E+03	2.8545E+03	2.9153E+03	2.8858E+03	3.1229E+03	2.9959E+03	2.9068E+03	2.9823E+03	2.8105E+03
	std	1.3714E+02	4.2042E+01	5.9310E+01	1.3489E+02	3.0607E+01	2.6979E+01	6.0736E+01	9.3836E+01	7.5485E+01	1.0876E+02	6.2766E+01	1.0911E+01
F24	mean	3.5567E+03	3.1789E+03	3.1101E+03	3.3043E+03	3.0163E+03	3.0828E+03	3.1289E+03	3.2329E+03	3.1792E+03	3.0149E+03	3.1596E+03	2.9746E+03
	std	1.4668E+02	6.6204E+01	4.3344E+01	1.2968E+02	3.3545E+01	2.8095E+01	3.1022E+01	6.8977E+01	9.4571E+01	5.5961E+01	4.4857E+01	1.1013E+01
F25	mean	3.0027E+03	3.0836E+03	3.0825E+03	3.0689E+03	3.0911E+03	3.2537E+03	3.0957E+03	3.0693E+03	3.0562E+03	3.0869E+03	3.1103E+03	3.0042E+03
	std	4.4659E+01	1.5331E+01	3.9171E+01	2.2772E+01	2.6649E+01	5.6490E+01	4.1876E+01	2.8111E+01	3.8632E+01	2.9536E+01	2.9909E+01	1.8664E+01
F26	mean	7.8035E+03	4.9282E+03	5.9942E+03	6.6139E+03	4.6977E+03	5.5939E+03	4.8678E+03	8.4416E+03	6.9803E+03	4.9350E+03	6.3950E+03	3.9103E+03
	std	3.5770E+03	3.1202E+02	4.4331E+02	3.8988E+03	1.5179E+03	1.3525E+03	4.5494E+02	1.4664E+03	7.4073E+02	6.7021E+02	4.4875E+02	7.8372E+02
F27	mean	3.7132E+03	3.3943E+03	3.5050E+03	3.6550E+03	3.3226E+03	3.5412E+03	3.3983E+03	3.6203E+03	3.6621E+03	3.3020E+03	3.5016E+03	3.2651E+03
	std	6.3092E+02	4.2860E+01	7.5036E+01	2.0771E+02	6.2768E+01	7.1556E+01	3.9746E+01	1.6698E+02	1.5961E+02	4.6206E+01	1.0129E+02	1.3745E+01
F28	mean	3.2824E+03	3.3740E+03	3.3630E+03	3.3220E+03	3.3597E+03	3.6476E+03	3.4776E+03	3.3213E+03	3.3031E+03	3.3519E+03	3.4169E+03	3.2623E+03
	std	2.3438E+01	3.4376E+01	4.2185E+01	2.8548E+01	4.2413E+01	8.7294E+01	9.8703E+01	2.6040E+01	1.3205E+01	3.7753E+01	4.1684E+01	2.2131E+00
F29	mean	5.0638E+03	4.2969E+03	4.7798E+03	5.0705E+03	3.7750E+03	4.2227E+03	3.6782E+03	4.8365E+03	4.6860E+03	3.8038E+03	4.5699E+03	3.4848E+03
	std	4.3460E+02	4.2552E+02	3.8333E+02	5.6584E+02	2.6009E+02	3.1624E+02	2.2391E+02	3.6911E+02	4.5430E+02	2.0016E+02	3.0719E+02	9.8611E+01
F30	mean	5.1526E+06	2.7008E+06	2.1275E+07	1.6095E+06	9.8761E+05	1.6054E+06	1.2301E+06	9.0056E+05	1.0055E+06	8.7491E+06	3.5887E+06	7.3057E+05
	std	1.9581E+06	1.5948E+06	6.9936E+06	5.9062E+05	2.4022E+05	3.5198E+05	2.8800E+05	2.2682E+05	3.0049E+05	2.9860E+06	1.4811E+06	4.5269E+04

**Table 9:** Results of various algorithms tested on the CEC 2017 benchmark (dim = 100).

ID	Metric	PSO	SO	RIME	DOA	SBOA	GRO	ESC	INFO	HHWOA	IGWO	CBSO	MICBSO
F1	mean	1.3458E+08	2.2089E+09	9.5809E+07	1.9059E+09	5.0579E+08	3.0467E+10	7.8195E+09	5.4101E+08	7.4666E+03	8.5098E+09	8.7681E+09	6.2200E+07
	std	2.8513E+07	4.9585E+08	2.4757E+07	3.4608E+09	1.5641E+09	8.2422E+09	2.6020E+09	1.5608E+09	1.0348E+04	3.9533E+09	2.2157E+09	1.0134E+07
F2	mean	2.6058E+72	1.7907E+129	3.3966E+105	6.2752E+109	1.0971E+95	2.5835E+127	4.1959E+124	8.3033E+123	4.4236E+118	4.3815E+107	1.2561E+123	1.8457E+97
	std	1.3465E+73	9.7391E+129	1.8344E+106	3.4350E+110	5.7019E+95	1.4150E+128	2.2963E+125	4.5479E+124	2.3529E+119	2.3998E+108	6.8802E+123	3.3491E+97
F3	mean	3.9472E+05	3.2782E+05	5.0477E+05	5.5522E+05	2.5111E+05	3.1678E+05	4.8199E+05	1.9475E+05	3.1126E+05	2.7170E+05	1.7146E+05	4.9407E+05
	std	4.3936E+04	1.2382E+04	5.6369E+04	1.1413E+05	2.1842E+04	3.6399E+04	6.4267E+04	2.5336E+04	5.5494E+04	4.1611E+04	2.3564E+04	3.9264E+04
F4	mean	6.9326E+02	9.2126E+02	9.2038E+02	9.5091E+02	9.2720E+02	3.0562E+03	1.5142E+03	8.9010E+02	7.0716E+02	1.3731E+03	1.6065E+03	6.7925E+02
	std	8.5833E+01	4.7581E+01	7.4913E+01	1.1186E+02	7.9032E+01	7.9086E+02	3.3516E+02	1.1423E+02	4.2954E+01	2.3744E+02	2.2732E+02	1.4450E+01
F5	mean	1.2647E+03	9.6063E+02	1.0526E+03	1.3761E+03	1.0278E+03	1.2077E+03	1.0222E+03	1.2725E+03	1.0830E+03	9.5664E+02	1.1557E+03	8.8173E+02
	std	5.6931E+01	8.9365E+01	8.7869E+01	1.4217E+02	7.2964E+01	7.8022E+01	1.3771E+02	7.9021E+01	7.3152E+01	1.0058E+02	7.3272E+01	2.8733E+01
F6	mean	6.6517E+02	6.0970E+02	6.3621E+02	6.8343E+02	6.2469E+02	6.3968E+02	6.0649E+02	6.5451E+02	6.3697E+02	6.1109E+02	6.4641E+02	6.0757E+02
	std	4.5048E+00	9.9950E−01	5.6908E+00	9.6018E+00	5.1906E+00	6.3777E+00	1.7602E+00	6.4023E+00	6.7457E+00	2.1919E+00	4.0127E+00	7.1453E−01
F7	mean	1.9967E+03	1.3185E+03	1.6608E+03	2.9973E+03	1.9017E+03	2.1270E+03	1.5476E+03	2.7019E+03	2.2729E+03	1.5321E+03	1.9297E+03	1.8790E+03
	std	1.4347E+02	5.2344E+01	1.3758E+02	2.2099E+02	1.9583E+02	1.7870E+02	1.2062E+02	2.3626E+02	2.2052E+02	1.4462E+02	1.4195E+02	3.0239E+01
F8	mean	1.6673E+03	1.2519E+03	1.3641E+03	1.7777E+03	1.3078E+03	1.5062E+03	1.3836E+03	1.6452E+03	1.4058E+03	1.2674E+03	1.4810E+03	1.1693E+03
	std	9.1554E+01	8.7860E+01	9.0533E+01	1.5298E+02	4.5761E+01	6.1611E+01	1.0119E+02	9.5991E+01	7.7934E+01	1.2097E+02	7.3350E+01	3.2216E+01
F9	mean	6.0656E+04	2.6457E+03	2.5253E+04	7.0096E+04	1.8896E+04	2.0268E+04	6.1721E+03	2.0906E+04	1.3122E+04	2.1351E+04	2.1019E+04	4.1267E+03
	std	7.1218E+03	4.1024E+02	1.0018E+04	6.7081E+03	3.4960E+03	4.3840E+03	1.4238E+03	2.5879E+03	2.7299E+03	8.7584E+03	3.2357E+03	7.1134E+02
F10	mean	1.5773E+04	3.0717E+04	1.7078E+04	2.8923E+04	1.4610E+04	1.9020E+04	2.7824E+04	1.6194E+04	1.5963E+04	2.6470E+04	1.9981E+04	1.3725E+04
	std	1.4518E+03	1.4418E+03	1.6445E+03	4.3010E+03	1.5426E+03	1.2395E+03	1.2820E+03	1.7976E+03	1.3776E+03	7.2126E+03	1.1108E+03	7.7882E+02
F11	mean	5.7471E+03	8.9848E+04	7.3803E+03	2.8129E+04	1.3680E+04	5.3280E+04	4.0770E+04	4.3414E+03	2.3017E+03	1.2776E+04	1.1266E+04	8.6985E+04
	std	1.0700E+03	2.3716E+04	1.5185E+03	1.5953E+04	4.8999E+03	9.3796E+03	1.0779E+04	1.1763E+03	2.8001E+02	4.7997E+03	4.2382E+03	9.0938E+03
F12	mean	2.2711E+08	4.3572E+08	6.9617E+08	5.9759E+07	4.9571E+07	1.1975E+09	5.0701E+08	4.1097E+07	7.0055E+06	4.4698E+08	7.7970E+08	1.8253E+07
	std	8.7203E+07	1.4063E+08	3.6702E+08	3.5715E+07	2.3212E+07	6.2351E+08	3.5206E+08	1.9599E+07	2.4855E+06	1.7413E+08	2.2797E+08	3.1331E+06
F13	mean	2.1153E+05	2.1661E+06	3.0265E+05	1.9185E+04	9.6800E+03	1.3433E+06	3.5899E+04	2.5771E+04	1.0123E+04	5.2503E+05	2.2036E+06	2.4825E+03
	std	8.6526E+04	2.9984E+06	9.5761E+04	1.1944E+04	6.2751E+03	3.6817E+06	1.2363E+04	4.8678E+04	4.0467E+03	2.2478E+05	3.6793E+06	1.9921E+02
F14	mean	1.5872E+06	3.7638E+06	3.9603E+06	1.2417E+06	1.7943E+06	2.6597E+06	5.5346E+06	4.0803E+05	8.9000E+04	1.2066E+06	4.0284E+03	3.0764E+05
	std	5.7503E+05	3.4174E+06	2.0283E+06	7.6407E+05	9.4170E+05	1.4262E+06	3.6870E+06	1.9386E+05	4.3266E+04	6.4488E+05	4.9661E+03	7.6289E+04
F15	mean	3.8695E+04	5.8283E+04	3.7802E+05	7.4480E+03	4.4866E+03	7.9690E+03	1.6455E+04	6.4373E+03	9.4608E+03	1.3343E+05	3.1385E+04	1.9565E+03
	std	1.8335E+04	6.0701E+04	1.0857E+06	5.5348E+03	2.3442E+03	2.5974E+03	5.3273E+03	4.6473E+03	8.3018E+03	6.4895E+04	9.0709E+03	6.0942E+01
F16	mean	5.7681E+03	8.8535E+03	6.6319E+03	6.3618E+03	4.9905E+03	5.9127E+03	6.5779E+03	6.0864E+03	5.6119E+03	4.8708E+03	6.4572E+03	4.0446E+03
	std	6.3054E+02	1.4518E+03	7.5656E+02	9.5908E+02	6.5833E+02	6.8844E+02	7.7747E+02	7.5048E+02	9.3875E+02	6.1415E+02	6.5878E+02	2.4737E+02
F17	mean	4.9785E+03	5.7100E+03	5.6358E+03	5.9585E+03	4.4775E+03	4.5297E+03	5.0077E+03	5.5281E+03	5.3322E+03	4.2313E+03	5.1960E+03	3.7326E+03
	std	6.5182E+02	8.8903E+02	7.3455E+02	1.0401E+03	4.1535E+02	5.6919E+02	4.3801E+02	4.5782E+02	5.7133E+02	1.0164E+03	4.5382E+02	2.4074E+02
F18	mean	3.3112E+06	5.9029E+06	6.5430E+06	1.3577E+06	3.1534E+06	4.9541E+06	5.6343E+06	5.8775E+05	3.0133E+05	2.6419E+06	8.2746E+04	1.3017E+06
	std	1.4890E+06	3.2575E+06	3.4882E+06	7.6396E+05	1.8059E+06	2.5352E+06	3.2444E+06	3.3664E+05	1.4599E+05	1.1465E+06	4.2468E+04	2.6117E+05
F19	mean	2.2663E+05	1.8556E+05	8.2628E+06	5.2832E+03	6.4210E+03	2.2231E+04	3.7203E+04	6.7542E+03	1.0887E+04	2.8406E+05	2.5232E+05	2.1663E+03
	std	1.9720E+05	2.7895E+05	4.2071E+06	3.2140E+03	6.3357E+03	6.5604E+04	3.8298E+04	3.8456E+03	9.6822E+03	2.6184E+05	2.4127E+05	6.9860E+01
F20	mean	4.9648E+03	6.9513E+03	5.2805E+03	7.0857E+03	4.4955E+03	4.6770E+03	5.7106E+03	5.3892E+03	5.3947E+03	5.8273E+03	5.3309E+03	3.6570E+03
	std	5.0301E+02	2.2217E+02	6.2630E+02	5.3183E+02	5.2760E+02	3.3044E+02	3.4896E+02	6.0191E+02	4.8965E+02	1.5691E+03	3.9118E+02	2.0702E+02
F21	mean	3.5347E+03	2.7886E+03	2.9225E+03	3.5371E+03	2.7606E+03	2.9369E+03	2.8718E+03	3.2298E+03	2.9779E+03	2.7708E+03	3.0238E+03	2.6862E+03
	std	1.7435E+02	1.1353E+02	8.4552E+01	2.1386E+02	6.2348E+01	6.1420E+01	1.2584E+02	1.4939E+02	9.9472E+01	1.4196E+02	9.5321E+01	2.2163E+01
F22	mean	1.9412E+04	3.2943E+04	1.9409E+04	3.2100E+04	1.8069E+04	2.1421E+04	2.9846E+04	1.9885E+04	1.9019E+04	2.5914E+04	2.2413E+04	1.5189E+04
	std	1.5309E+03	1.6621E+03	1.6183E+03	5.6721E+03	3.2841E+03	2.7455E+03	1.0790E+03	1.9682E+03	1.3577E+03	7.9321E+03	1.3698E+03	1.8222E+03
F23	mean	4.8016E+03	3.2108E+03	3.4410E+03	4.0162E+03	3.2216E+03	3.5943E+03	3.1291E+03	3.8008E+03	3.6321E+03	3.2249E+03	3.6254E+03	3.1031E+03
	std	2.7902E+02	5.6962E+01	9.8873E+01	2.0988E+02	5.9925E+01	7.1101E+01	5.8104E+01	1.4938E+02	1.3100E+02	1.1220E+02	1.0496E+02	1.4634E+01
F24	mean	5.3578E+03	3.6853E+03	3.9769E+03	5.0814E+03	3.8378E+03	4.3044E+03	3.6646E+03	4.6210E+03	4.4803E+03	3.7311E+03	4.1930E+03	3.5496E+03
	std	3.4247E+02	6.9039E+01	8.9133E+01	4.8020E+02	1.0338E+02	1.4043E+02	9.0772E+01	2.5821E+02	2.7898E+02	1.2835E+02	1.4340E+02	1.6432E+01
F25	mean	3.3774E+03	3.7698E+03	3.6384E+03	3.6740E+03	3.6065E+03	5.3035E+03	4.4831E+03	3.5593E+03	3.3625E+03	4.0918E+03	4.3491E+03	3.4023E+03
	std	3.9692E+01	7.6078E+01	7.7605E+01	1.0572E+02	7.0508E+01	5.2412E+02	3.0577E+02	1.0135E+02	5.1431E+01	1.7351E+02	2.0366E+02	1.9404E+01
F26	mean	1.6083E+04	1.0033E+04	1.3077E+04	2.1516E+04	1.5083E+04	2.0039E+04	9.7205E+03	2.1645E+04	1.7199E+04	1.1020E+04	1.5608E+04	8.7168E+03
	std	8.5673E+03	5.8382E+02	1.3657E+03	6.2972E+03	3.8639E+03	2.9553E+03	6.2346E+02	3.0671E+03	1.9646E+03	1.5598E+03	1.1626E+03	1.8779E+02
F27	mean	3.5963E+03	3.6255E+03	3.8025E+03	3.8281E+03	3.6278E+03	4.0724E+03	3.6883E+03	3.8934E+03	4.0533E+03	3.5204E+03	3.8196E+03	3.3621E+03
	std	4.1395E+02	6.1942E+01	1.0682E+02	2.1055E+02	8.3996E+01	1.5255E+02	9.0405E+01	1.8611E+02	2.4692E+02	5.2420E+01	1.7207E+02	1.1902E+01
F28	mean	3.4170E+03	4.2971E+03	3.6940E+03	3.9274E+03	3.7697E+03	6.7537E+03	6.2766E+03	3.6866E+03	3.4596E+03	4.5943E+03	5.0790E+03	3.4613E+03
	std	5.5356E+01	2.6869E+02	5.8305E+01	3.3702E+02	1.3825E+02	7.5636E+02	6.9478E+02	1.0765E+02	4.1406E+01	4.7128E+02	5.1716E+02	1.7890E+01
F29	mean	8.2170E+03	7.1691E+03	8.8964E+03	7.7713E+03	6.4096E+03	7.5557E+03	6.3075E+03	7.9646E+03	7.6432E+03	6.3430E+03	8.1710E+03	5.3127E+03
	std	6.0379E+02	6.5341E+02	7.1453E+02	1.0999E+03	6.2816E+02	4.1840E+02	7.6966E+02	5.1470E+02	6.9136E+02	7.1354E+02	8.0597E+02	2.2743E+02
F30	mean	6.7917E+06	3.9748E+06	6.9370E+07	1.8505E+05	4.7536E+04	4.2046E+06	4.1593E+06	3.5405E+05	2.5573E+04	1.1850E+07	1.6967E+07	1.1548E+04
	std	2.5484E+06	2.6975E+06	3.8285E+07	1.8967E+05	2.1598E+04	6.2487E+06	3.2635E+06	1.1068E+06	2.0181E+04	3.7984E+06	1.1623E+07	1.5517E+03

**Table 10:** *p*-values for various algorithms on the CEC 2017 (dim = 30).

Item	PSO	SO	RIME	DOA	SBOA	GRO	ESC	INFO	HHWOA	IGWO	CBSO
F1	3.0199E−11	3.0199E−11	3.0199E−11	1.5581E−08	2.3897E−08	3.0199E−11	1.8731E−07	1.1738E−03	6.0104E−08	3.0199E−11	3.0199E−11
F2	1.0702E−09	3.0199E−11	3.0199E−11	2.1540E−06	6.1210E−10	3.0199E−11	3.0199E−11	3.0199E−11	3.0199E−11	3.0199E−11	3.0199E−11
F3	3.0199E−11	3.8481E−03	3.0199E−11	2.8129E−02	3.0199E−11	1.2541E−07	2.3985E−01	3.0199E−11	3.0199E−11	3.0199E−11	3.0199E−11
F4	2.3985E−01	3.0199E−11	1.0937E−10	7.2951E−04	1.6813E−04	4.1825E−09	1.5581E−08	7.2827E−01	4.2039E−01	1.0105E−08	8.8910E−10
F5	3.0199E−11	1.8731E−07	3.0199E−11	3.0199E−11	3.0199E−11	3.0199E−11	2.3715E−10	3.0199E−11	3.3384E−11	3.0199E−11	3.0199E−11
F6	3.0199E−11	3.0199E−11	3.0199E−11	3.0199E−11	3.0199E−11	3.0199E−11	3.0199E−11	3.0199E−11	3.0199E−11	3.0199E−11	3.0199E−11
F7	3.0199E−11	2.0152E−08	3.0199E−11	3.0199E−11	2.1544E−10	3.0199E−11	3.0199E−11	3.0199E−11	3.0199E−11	5.0723E−10	3.0199E−11
F8	3.0180E−11	3.3505E−08	1.6123E−10	3.0180E−11	3.4722E−10	3.0180E−11	2.4374E−09	3.0180E−11	2.3701E−10	7.7688E−09	3.0180E−11
F9	3.0180E−11	3.0180E−11	3.0180E−11	3.0180E−11	3.0180E−11	3.0180E−11	3.6436E−02	3.0180E−11	3.0180E−11	3.0180E−11	3.0180E−11
F10	3.0199E−11	3.0199E−11	5.4941E−11	3.0199E−11	6.0104E−08	3.0199E−11	3.0199E−11	3.0199E−11	4.6159E−10	7.3891E−11	3.0199E−11
F11	3.0199E−11	3.0199E−11	3.0199E−11	3.0199E−11	2.6099E−10	3.0199E−11	1.8500E−08	3.0199E−11	3.0199E−11	1.3289E−10	3.0199E−11
F12	5.5727E−10	6.7220E−10	3.0199E−11	1.1738E−03	5.9673E−09	8.9934E−11	3.4742E−10	1.3732E−01	6.6689E−03	3.0199E−11	1.2870E−09
F13	2.1947E−08	3.0199E−11	3.0199E−11	1.8567E−09	8.1975E−07	4.9752E−11	2.9215E−09	1.9568E−10	1.6980E−08	3.0199E−11	1.1228E−02
F14	9.5332E−07	6.0459E−07	1.0702E−09	2.6243E−03	1.1747E−04	2.8913E−03	1.4918E−06	6.0459E−07	3.0199E−11	3.8481E−03	3.0199E−11
F15	3.0199E−11	3.0199E−11	3.0199E−11	1.0937E−10	1.2057E−10	3.0199E−11	3.8202E−10	3.0199E−11	7.2446E−02	3.0199E−11	1.6062E−06
F16	3.0199E−11	4.1825E−09	1.6132E−10	3.0199E−11	3.7704E−04	8.5641E−04	4.3531E−05	3.0199E−11	2.9215E−09	3.7782E−02	2.0283E−07
F17	3.0199E−11	4.0772E−11	7.3891E−11	3.0199E−11	1.6947E−09	5.4617E−09	1.3272E−02	3.0199E−11	3.3384E−11	7.6588E−05	3.0199E−11
F18	1.4110E−09	9.7555E−10	1.6132E−10	3.1119E−01	5.5329E−08	7.0881E−08	2.6099E−10	2.4327E−05	3.3384E−11	6.2828E−06	3.0199E−11
F19	2.6099E−10	7.3891E−11	3.0199E−11	1.0937E−10	9.7555E−10	1.0702E−09	9.8329E−08	2.5306E−04	3.5708E−06	3.0199E−11	4.7138E−04
F20	3.0199E−11	1.1711E−02	1.7290E−06	3.0199E−11	4.0595E−02	2.3897E−08	4.6371E−03	1.4110E−09	4.1825E−09	2.4157E−02	3.1589E−10
F21	3.0199E−11	8.1014E−10	3.0199E−11	3.0199E−11	3.3520E−08	6.0658E−11	3.6897E−11	3.0199E−11	3.0199E−11	2.4913E−06	3.0199E−11
F22	3.0199E−11	3.0199E−11	3.0199E−11	3.0199E−11	3.0199E−11	3.0199E−11	3.0199E−11	5.6073E−05	1.9527E−03	3.0199E−11	3.0199E−11
F23	3.0199E−11	3.0199E−11	3.0199E−11	3.0199E−11	6.9724E−03	2.0338E−09	1.4423E−03	3.0199E−11	3.0199E−11	2.2539E−04	3.0199E−11
F24	3.0199E−11	3.0199E−11	3.0199E−11	3.0199E−11	3.8481E−03	2.5721E−07	3.0199E−11	3.0199E−11	3.0199E−11	6.6689E−03	3.0199E−11
F25	3.7782E−02	3.0199E−11	1.2057E−10	1.6062E−06	1.8567E−09	2.3715E−10	3.0199E−11	4.6159E−10	1.2057E−10	8.8910E−10	8.8910E−10
F26	6.5486E−04	4.1825E−09	1.6947E−09	1.7294E−07	7.7387E−06	1.1228E−02	3.5137E−02	6.5183E−09	5.5727E−10	8.3146E−03	3.1573E−05
F27	7.6183E−01	3.0199E−11	3.0199E−11	1.3289E−10	1.0035E−03	3.0199E−11	4.4440E−07	3.0199E−11	2.3715E−10	2.9205E−02	4.1997E−10
F28	5.0723E−10	3.0199E−11	3.0199E−11	1.5964E−07	2.6099E−10	3.0199E−11	3.0199E−11	7.0881E−08	5.6922E−01	3.0199E−11	3.0199E−11
F29	3.0199E−11	2.1947E−08	5.4941E−11	8.1527E−11	7.2884E−03	1.7769E−10	5.6922E−01	3.0199E−11	3.0199E−11	5.7929E−01	5.5727E−10
F30	3.0199E−11	1.9568E−10	3.0199E−11	8.8910E−10	3.0199E−11	3.0199E−11	3.6897E−11	6.0104E−08	1.4110E−09	3.0199E−11	5.0723E−10

**Table 11:** *p*-values for various algorithms on the CEC 2017 (dim = 50).

Item	PSO	SO	RIME	DOA	SBOA	GRO	ESC	INFO	HHWOA	IGWO	CBSO
F1	3.0199E−11	3.0199E−11	3.0199E−11	3.0199E−11	3.0199E−11	3.0199E−11	3.0199E−11	2.6695E−09	1.2493E−05	3.0199E−11	3.0199E−11
F2	3.0199E−11	3.0199E−11	5.1060E−01	6.0971E−03	5.9428E−02	3.0199E−11	3.0199E−11	1.2870E−09	1.3853E−06	3.4742E−10	3.0199E−11
F3	3.0199E−11	4.5726E−09	3.6897E−11	1.2362E−03	3.0199E−11	3.6897E−11	8.3026E−01	3.0199E−11	3.0199E−11	3.0199E−11	3.0199E−11
F4	4.4440E−07	3.0199E−11	3.0199E−11	2.5721E−07	2.0283E−07	3.0199E−11	4.9752E−11	4.8011E−07	4.9818E−04	3.6897E−11	3.0199E−11
F5	3.0199E−11	3.0199E−11	3.0199E−11	3.0199E−11	4.0772E−11	3.0199E−11	8.1014E−10	3.0199E−11	3.0199E−11	3.0199E−11	3.0199E−11
F6	3.0199E−11	3.0199E−11	3.0199E−11	3.0199E−11	3.0199E−11	3.0199E−11	2.3800E−03	3.0199E−11	3.0199E−11	3.0199E−11	3.0199E−11
F7	1.6687E−01	3.6897E−11	1.4733E−07	3.0199E−11	4.4205E−06	1.8608E−06	3.4971E−09	3.0199E−11	9.5207E−04	2.5974E−05	4.4205E−06
F8	3.0199E−11	3.0199E−11	3.0199E−11	3.0199E−11	3.0199E−11	3.0199E−11	5.5727E−10	3.0199E−11	3.0199E−11	1.2057E−10	3.0199E−11
F9	3.0199E−11	3.0199E−11	3.0199E−11	3.0199E−11	3.0199E−11	3.0199E−11	3.3384E−11	3.0199E−11	3.0199E−11	3.0199E−11	3.0199E−11
F10	3.0199E−11	3.0199E−11	3.3384E−11	3.0199E−11	4.8011E−07	3.0199E−11	3.0199E−11	3.0199E−11	3.0199E−11	2.6695E−09	3.0199E−11
F11	1.4067E−04	3.0199E−11	3.0199E−11	1.4294E−08	6.2040E−01	3.0199E−11	3.3520E−08	3.8307E−05	9.2603E−09	1.9568E−10	5.4941E−11
F12	3.0199E−11	3.0199E−11	3.0199E−11	1.1567E−07	8.1014E−10	3.0199E−11	3.4742E−10	3.1466E−02	2.3399E−01	3.0199E−11	3.0199E−11
F13	3.0199E−11	3.0199E−11	3.0199E−11	3.0199E−11	1.5465E−09	3.0199E−11	3.0199E−11	3.0199E−11	9.9186E−11	3.0199E−11	3.0199E−11
F14	8.1527E−11	8.1014E−10	3.0199E−11	5.0922E−08	3.0199E−11	3.0199E−11	3.0199E−11	2.5101E−02	9.5332E−07	7.1186E−09	3.0199E−11
F15	6.5261E−07	3.3384E−11	3.0199E−11	2.6784E−06	8.4848E−09	1.8500E−08	2.0283E−07	9.7555E−10	2.6015E−08	3.0199E−11	1.9579E−01
F16	3.3384E−11	3.4742E−10	3.0199E−11	3.0199E−11	3.0939E−06	9.7917E−05	8.1527E−11	3.6897E−11	1.9568E−10	2.2823E−01	2.6099E−10
F17	3.0199E−11	3.0199E−11	3.0199E−11	3.0199E−11	3.3520E−08	1.7769E−10	4.6856E−08	3.0199E−11	3.0199E−11	9.8834E−03	4.9752E−11
F18	9.7555E−10	1.2057E−10	1.9568E−10	4.7335E−01	2.3768E−07	5.4941E−11	3.0199E−11	2.3768E−07	4.5043E−11	4.3531E−05	3.0199E−11
F19	4.6856E−08	9.2603E−09	3.0199E−11	3.7704E−04	6.5671E−02	3.5201E−07	4.0840E−05	2.5721E−07	9.5332E−07	3.0199E−11	9.0632E−08
F20	3.0199E−11	3.0199E−11	3.0199E−11	3.0199E−11	1.5964E−07	1.6947E−09	5.0723E−10	3.0199E−11	5.4941E−11	4.6390E−05	6.1210E−10
F21	3.0199E−11	3.0199E−11	3.0199E−11	3.0199E−11	4.6159E−10	3.0199E−11	3.0199E−11	3.0199E−11	3.0199E−11	1.2057E−10	3.0199E−11
F22	3.0199E−11	3.0199E−11	3.0199E−11	2.8716E−10	8.1975E−07	1.9527E−03	3.0199E−11	3.0199E−11	3.0199E−11	1.4643E−10	3.0199E−11
F23	3.0199E−11	1.0702E−09	3.0199E−11	3.0199E−11	7.1186E−09	3.0199E−11	1.7479E−05	3.0199E−11	3.0199E−11	3.9648E−08	3.0199E−11
F24	3.0199E−11	3.0199E−11	3.0199E−11	3.0199E−11	1.8567E−09	3.0199E−11	3.0199E−11	3.0199E−11	3.0199E−11	9.5332E−07	3.0199E−11
F25	6.2040E−01	3.0199E−11	1.9568E−10	7.3891E−11	3.0199E−11	3.0199E−11	4.6159E−10	3.1967E−09	6.0459E−07	3.0199E−11	3.0199E−11
F26	1.2477E−04	2.9215E−09	3.0199E−11	3.1466E−02	3.0339E−03	1.6351E−05	5.1857E−07	1.9568E−10	3.0199E−11	1.3111E−08	3.0199E−11
F27	1.0000E+00	3.0199E−11	3.0199E−11	3.0199E−11	1.8608E−06	3.0199E−11	3.0199E−11	3.0199E−11	3.0199E−11	2.7726E−05	3.0199E−11
F28	3.5201E−07	3.0199E−11	3.0199E−11	3.0199E−11	3.3384E−11	3.0199E−11	3.0199E−11	3.0199E−11	1.6132E−10	3.0199E−11	3.0199E−11
F29	3.0199E−11	3.0199E−11	3.0199E−11	3.0199E−11	1.2493E−05	3.0199E−11	5.8737E−04	3.0199E−11	9.9186E−11	4.5726E−09	3.0199E−11
F30	3.0199E−11	3.0199E−11	3.0199E−11	3.6897E−11	1.2541E−07	3.0199E−11	3.0199E−11	1.4423E−03	1.3703E−03	3.0199E−11	3.0199E−11

**Table 12:** *p*-values for various algorithms on the CEC 2017 (dim = 100).

Item	PSO	SO	RIME	DOA	SBOA	GRO	ESC	INFO	HHWOA	IGWO	CBSO
F1	3.0199E−11	3.0199E−11	7.6950E−08	3.0199E−11	1.5798E−01	3.0199E−11	3.0199E−11	2.4327E−05	3.0199E−11	3.0199E−11	3.0199E−11
F2	3.0199E−11	3.0199E−11	2.8129E−02	5.9428E−02	5.4617E−09	3.0199E−11	5.4941E−11	2.1947E−08	1.0666E−07	2.5805E−01	1.3289E−10
F3	1.8567E−09	3.0199E−11	3.5547E−01	5.5699E−03	3.0199E−11	3.0199E−11	2.7719E−01	3.0199E−11	4.5043E−11	3.0199E−11	3.0199E−11
F4	2.5188E−01	3.0199E−11	3.0199E−11	3.0199E−11	3.0199E−11	3.0199E−11	3.0199E−11	3.0199E−11	8.5641E−04	3.0199E−11	3.0199E−11
F5	3.0199E−11	8.8829E−06	9.9186E−11	3.0199E−11	3.6897E−11	3.0199E−11	6.7362E−06	3.0199E−11	3.0199E−11	1.8682E−05	3.0199E−11
F6	3.0199E−11	4.1997E−10	3.0199E−11	3.0199E−11	3.0199E−11	3.0199E−11	7.6973E−04	3.0199E−11	3.0199E−11	1.5581E−08	3.0199E−11
F7	1.0407E−04	3.0199E−11	1.0702E−09	3.0199E−11	8.1875E−01	8.4848E−09	3.0199E−11	3.0199E−11	7.3803E−10	3.8249E−09	1.5798E−01
F8	3.0199E−11	1.6813E−04	2.8716E−10	3.0199E−11	3.0199E−11	3.0199E−11	2.6695E−09	3.0199E−11	3.0199E−11	7.6950E−08	3.0199E−11
F9	3.0199E−11	1.6947E−09	3.0199E−11	3.0199E−11	3.0199E−11	3.0199E−11	3.0103E−07	3.0199E−11	3.0199E−11	3.0199E−11	3.0199E−11
F10	1.1567E−07	3.0199E−11	4.5043E−11	4.0772E−11	4.3584E−02	3.0199E−11	3.0199E−11	4.1825E−09	2.0283E−07	4.5726E−09	3.0199E−11
F11	3.0199E−11	9.3519E−01	3.0199E−11	3.3384E−11	3.0199E−11	3.6897E−11	3.0199E−11	3.0199E−11	3.0199E−11	3.0199E−11	3.0199E−11
F12	3.0199E−11	3.0199E−11	3.0199E−11	1.4110E−09	3.0811E−08	3.0199E−11	3.0199E−11	1.4294E−08	5.4941E−11	3.0199E−11	3.0199E−11
F13	3.0199E−11	3.0199E−11	3.0199E−11	3.0199E−11	3.0199E−11	3.0199E−11	3.0199E−11	3.0199E−11	3.0199E−11	3.0199E−11	3.0199E−11
F14	3.0199E−11	6.6955E−11	3.0199E−11	1.1023E−08	3.0199E−11	1.7769E−10	3.0199E−11	4.2067E−02	7.3891E−11	1.9568E−10	3.0199E−11
F15	3.0199E−11	3.0199E−11	3.0199E−11	3.0199E−11	3.0199E−11	3.0199E−11	3.0199E−11	3.0199E−11	3.0199E−11	3.0199E−11	3.0199E−11
F16	3.0199E−11	3.0199E−11	3.0199E−11	3.0199E−11	1.5581E−08	3.3384E−11	3.0199E−11	3.0199E−11	5.4617E−09	1.6980E−08	3.0199E−11
F17	6.6955E−11	3.0199E−11	3.0199E−11	3.0199E−11	2.4386E−09	3.9648E−08	4.5043E−11	3.0199E−11	4.9752E−11	2.5101E−02	3.0199E−11
F18	1.1737E−09	2.6099E−10	3.0199E−11	5.7929E−01	3.8349E−06	3.3384E−11	6.6955E−11	3.8249E−09	3.0199E−11	7.7387E−06	3.0199E−11
F19	3.0199E−11	3.0199E−11	3.0199E−11	3.0199E−11	2.0338E−09	3.0199E−11	3.0199E−11	3.0199E−11	3.6897E−11	3.0199E−11	3.0199E−11
F20	3.0199E−11	3.0199E−11	3.0199E−11	3.0199E−11	5.4617E−09	3.0199E−11	3.0199E−11	3.0199E−11	3.0199E−11	4.9980E−09	3.0199E−11
F21	3.0199E−11	5.4620E−06	3.0199E−11	3.0199E−11	2.0023E−06	3.0199E−11	4.6856E−08	3.0199E−11	3.0199E−11	2.6806E−04	3.0199E−11
F22	3.0199E−11	3.0199E−11	3.0199E−11	5.5727E−10	5.9673E−09	5.0723E−10	3.0199E−11	1.0937E−10	3.6897E−11	3.4971E−09	3.0199E−11
F23	3.0199E−11	3.0199E−11	3.0199E−11	3.0199E−11	2.3715E−10	3.0199E−11	5.3685E−02	3.0199E−11	3.0199E−11	3.4742E−10	3.0199E−11
F24	3.0199E−11	3.0199E−11	3.0199E−11	3.0199E−11	3.0199E−11	3.0199E−11	1.8500E−08	3.0199E−11	3.0199E−11	3.0199E−11	3.0199E−11
F25	2.7548E−03	3.0199E−11	3.0199E−11	2.1544E−10	3.0199E−11	3.0199E−11	3.0199E−11	7.7725E−09	7.6973E−04	3.0199E−11	3.0199E−11
F26	7.2884E−03	3.0199E−11	3.0199E−11	1.0666E−07	5.5727E−10	3.0199E−11	1.4643E−10	3.0199E−11	3.0199E−11	5.5727E−10	3.0199E−11
F27	7.5991E−07	3.0199E−11	3.0199E−11	3.0199E−11	3.0199E−11	3.0199E−11	3.0199E−11	3.0199E−11	3.0199E−11	3.0199E−11	3.0199E−11
F28	3.8307E−05	3.0199E−11	3.0199E−11	3.0199E−11	3.0199E−11	3.0199E−11	3.0199E−11	3.0199E−11	7.7312E−01	3.0199E−11	3.0199E−11
F29	3.0199E−11	3.0199E−11	3.0199E−11	3.0199E−11	1.7769E−10	3.0199E−11	2.7829E−07	3.0199E−11	3.0199E−11	1.1737E−09	3.0199E−11
F30	3.0199E−11	3.0199E−11	3.0199E−11	3.0199E−11	3.3384E−11	3.0199E−11	3.0199E−11	3.0199E−11	4.4592E−04	3.0199E−11	3.0199E−11

**Table 13:** Friedman rankings for various algorithms.

Suites	CEC2017					
Dimensions	30		50		100	
Algorithms	M.R	T.R	M.R	T.R	M.R	T.R
PSO	8.17	10	7.63	9	6.90	6
SO	8.13	9	7.90	10	7.20	8
RIME	8.70	12	8.03	11	7.57	9
DOA	8.47	11	8.50	12	8.67	12
SBOA	4.70	2	4.20	2	4.20	2
GRO	6.83	7	7.07	6	8.37	11
ESC	5.57	3	6.13	5	6.93	7
INFO	7.77	8	7.47	8	6.70	5
HHWOA	5.80	5	6.00	3	5.17	3
IGWO	5.63	4	6.03	4	5.97	4
CBSO	6.43	6	7.10	7	7.93	10
MICBSO	1.80	1	1.93	1	2.40	1

**Table 14:** Analysis of ablation experiment results.

Suites	CEC2017					
Dimensions	30		50		100	
Algorithms	M.R	T.R	M.R	T.R	M.R	T.R
CBSO	3.73	5	3.70	5	4.03	5
CBSO+Chaos-OBL	3.37	4	3.33	3	3.43	4
CBSO+Elite Guidance	3.30	3	3.30	2	3.13	3
CBSO+Boundary Control	3.20	2	3.37	4	2.93	2
MICBSO	1.40	1	1.30	1	1.47	1

**Table 15:** Experimental results of path planning for each algorithm in Scenario 1.

Algorithm	PSO	SO	RIME	DOA	SBOA	GRO	ESC	INFO	HHWOA	IGWO	CBSO	MICBSO
mean	449.37	416.56	420.25	424.46	415.47	417.30	416.16	540.82	416.64	528.87	421.46	414.07
max	507.80	427.99	431.47	447.83	422.07	420.84	421.09	570.65	428.14	559.74	431.58	414.17
min	420.73	414.53	414.31	416.12	409.07	415.88	415.32	503.35	414.45	470.77	411.16	413.89

**Table 16:** Experimental results of path planning for each algorithm in Scenario 2.

Algorithm	PSO	SO	RIME	DOA	SBOA	GRO	ESC	INFO	HHWOA	IGWO	CBSO	MICBSO
mean	455.03	416.81	419.04	429.23	415.00	416.93	416.10	65,535	416.70	532.53	420.93	413.91
max	498.88	424.23	431.54	458.63	416.13	419.37	417.46	65,535	431.59	587.73	434.60	414.15
min	422.62	414.81	412.80	417.85	414.29	411.53	414.79	497.05	414.42	469.72	411.88	410.26

**Table 17:** Experimental results of path planning for each algorithm in Scenario 3.

Algorithm	PSO	SO	RIME	DOA	SBOA	GRO	ESC	INFO	HHWOA	IGWO	CBSO	MICBSO
mean	524.93	453.37	451.83	460.42	432.12	436.88	435.26	65,535	436.36	65,535	451.75	423.15
max	623.02	492.64	466.83	504.32	438.23	442.14	436.99	65,535	481.10	65,535	472.51	424.57
min	455.86	436.70	439.35	442.87	423.31	419.49	432.86	558.84	432.28	552.54	429.65	414.56

**Table 18:** Experimental results of path planning for each algorithm in Scenario 4.

Algorithm	PSO	SO	RIME	DOA	SBOA	GRO	ESC	INFO	HHWOA	IGWO	CBSO	MICBSO
mean	633.67	495.21	468.25	512.28	65,535	444.04	440.49	65,535	462.09	65,535	461.79	421.99
max	780.38	634.43	499.75	553.75	65,535	458.78	470.04	65,535	575.48	65,535	526.11	427.80
min	549.41	442.93	435.07	488.55	415.21	436.93	434.97	624.29	426.80	577.61	421.03	413.69

## Data Availability

All experimental data generated and analyzed in this study are original and self-produced by the authors. All data and code that support the findings of this study are available from the corresponding author upon reasonable request.

## Appendix A. Theoretical Convergence Analysis of MICBSO

Convergence is an important theoretical property for evaluating the reliability and effectiveness of metaheuristic optimization algorithms. For the proposed MICBSO, although its search process contains several stochastic operations, including chaotic initialization, opposition-based learning, Gaussian perturbation, random variables in the CBSO update phases, and hybrid boundary control, the population update at each iteration only depends on the current population state and newly generated random variables. Therefore, the search process of MICBSO can be analyzed using Markov chain theory. In this subsection, the convergence property of MICBSO is discussed from the perspectives of state transition, optimal state preservation, and global convergence.

**Definition** **A1.**
*Individual state and individual state space.*

For a minimization problem (Ω,f), let the feasible search space be defined as

(A1) $$\Omega =\prod_{j=1}^{D}\;[lb_{j},ub_{j}],$$

where D is the problem dimension, and $lb_{j}$ and $ub_{j}$ denote the lower and upper bounds of the j-th decision variable, respectively. The position of the i-th individual in MICBSO is denoted as

(A2) $$X_{i}=(x_{i,1},x_{i,2},\ldots ,x_{i,D})\in \Omega .$$

Under finite-precision computation, the feasible search space can be regarded as a finite state space, denoted as Y. Therefore, each individual state $X_{i}$ belongs to Y.

**Definition** **A2.**
*Population state and population state space.*

Assume that the population size is N. The population state of MICBSO at iteration t is defined as

(A3) $$S(t)=(X_{1}(t),X_{2}(t),\ldots ,X_{N}(t),X_{best}(t))$$

where $X_{i}(t)$ represents the i-th individual at iteration t, and $X_{best}(t)$ denotes the best solution found so far. The population state space of MICBSO is denoted as

(A4) $$S=\left\{S(t)|X_{i}(t)\in Y,i=1,2,\ldots ,N\right\}.$$

Since the individual state space Y is finite under finite-precision computation and the population size N is also finite, the population state space S is finite.

**Definition** **A3.**
*State transition of MICBSO.*

During each iteration, MICBSO generates a candidate solution for each individual through the CBSO update mechanism, the Gaussian perturbation-based multi-elite guidance mechanism, and the hybrid boundary control strategy. Let the trial solution of the i-th individual at iteration t be denoted as

(A5) $${\tilde{X}}_{i}(t+1)=\Psi \left(\Phi_{t}(X_{i}(t),S(t),\xi_{i}(t))\right),$$

where $\Phi_{t}(\cdot )$ represents the stochastic position update operator of MICBSO, $\xi_{i}(t)$ denotes the random variables generated at iteration t, and Ψ(⋅) represents the hybrid boundary control operator. For a minimization problem, the greedy selection rule can be expressed as:

(A6) $$X_{i}(t+1)=\left\{\begin{array}{c} {\tilde{X}}_{i}(t+1),\;\;\;\;\;\;f({\tilde{X}}_{i}(t+1))\leq f(X_{i}(t)),\\ X_{i}(t),\;\;\;\;\;\;\;\;\;\;\;f({\tilde{X}}_{i}(t+1))>f(X_{i}(t)). \end{array}\right.$$

Meanwhile, the historical best solution is preserved as

(A7) $$X_{best}(t+1)=argmin\left\{f(X_{best}(t)),f(X_{1}(t+1)),\ldots ,f(X_{N}(t+1))\right\}.$$

Therefore, the best fitness value obtained by MICBSO satisfies

(A8) $$f(X_{best}(t+1))\leq f(X_{best}(t)).$$

This indicates that the best fitness value of MICBSO is monotonically non-increasing during the iterative process.

**Theorem** **A1.**
*The population state sequence of MICBSO has the Markov property.*

The population state sequence $\left\{S(t),t\geq 0\right\}$ generated by MICBSO is a Markov chain.

**Proof.** At iteration t, the next population state S(t+1) is determined by the current population state S(t), the current elite information, the current best solution, and the random variables generated at the current iteration. The chaotic initialization and opposition-based learning only determine the initial population. After initialization, the subsequent state transition does not depend on the historical population states S(0),S(1),…,S(t−1). In addition, the random variables used in Gaussian perturbation, CBSO position update, and boundary control are independently generated at each iteration. Therefore, for any population states $s_{0},s_{1},\ldots ,s_{t},s_{t+1}\in S$, the following relationship holds:

(A9) $$P(S(t+1)=s_{t+1}|S(0)=s_{0},S(1)=s_{1},\ldots ,S(t)=s_{t})=P(S(t+1)=s_{t+1}|S(t)=s_{t}).$$

Thus, the population state sequence of MICBSO satisfies the Markov property. Since S is finite under finite-precision computation, the sequence $\left\{S(t),t\geq 0\right\}$ can be regarded as a finite Markov chain. Theorem A1 is proved. □

**Definition** **A4.**
*Optimal individual state set.*

Let $f^{*}$ be the global optimal fitness value of the optimization problem. For any ε>0, the ε-optimal individual state set is defined as

(A10) $$G_{\varepsilon }=\{X\in Y|f(X)\leq f^{*}+\varepsilon \}.$$

When ε=0, $G_{\varepsilon }$ degenerates into the global optimal solution set.

**Definition** **A5.**
*Optimal population state set.*

The ε-optimal population state set of MICBSO is defined as

(A11) $$H_{\varepsilon }=\left\{S\in S|X_{best}\in G_{\varepsilon }\right\}.$$

That is, if the best solution stored in the population state belongs to $G_{\varepsilon }$, then the corresponding population state belongs to $H_{\varepsilon }$.

**Lemma** **A1.** *The optimal population state set* $H_{\varepsilon }$ *is a closed set.*

**Proof.** According to the best solution preservation mechanism of MICBSO, the best solution found so far is retained during the iterative process. If $S(t)\in H_{\varepsilon }$, then $X_{best}(t)\in G_{\varepsilon }$, which means

(A12) $$f(X_{best}(t))\leq f^{*}+\varepsilon .$$

Since MICBSO preserves the historical best solution, we have

(A13) $$f(X_{best}(t+1))\leq f(X_{best}(t)).$$

Therefore,

(A14) $$f(X_{best}(t+1))\leq f^{*}+\varepsilon .$$

This implies that $X_{best}(t+1)\in G_{\varepsilon }$, and thus $S(t+1)\in H_{\varepsilon }$. Hence, once the population state enters $H_{\varepsilon }$, it will not leave this set. Therefore, $H_{\varepsilon }$ is a closed set. Lemma A1 is proved. □

**Lemma** **A2.**
*MICBSO has a positive probability of reaching the ε-optimal state set.*

For any population state $S(t)\notin H_{\varepsilon }$, there exists a positive probability that MICBSO generates at least one candidate solution in $G_{\varepsilon }$ after a finite number of iterations.

**Proof.** In MICBSO, the search process contains several stochastic operators. First, the chaos–opposition-based initialization strategy generates candidate solutions and their opposite solutions in the feasible search space, which increases the probability that the initial population covers promising regions. Second, the Gaussian perturbation-based multi-elite guidance mechanism introduces random perturbation around elite individuals, enabling the population to explore the neighborhood of high-quality solutions. Third, the original CBSO update mechanism contains random variables and stochastic search behaviors. Fourth, the hybrid boundary control strategy introduces random reinitialization for severe boundary violations, which allows individuals to be resampled within the feasible search space. Since the random variables used in these operators have nonzero probability density in the feasible region, for any nonempty region $G_{\varepsilon }\subseteq Y$, there exists a positive probability $q_{\varepsilon }>0$ such that at least one individual can be generated in $G_{\varepsilon }$ after a finite number of iterations. Therefore,

(A15) $$P(S(t+1)\in H_{\varepsilon }|S(t)\notin H_{\varepsilon }){≧}_{\varepsilon }>0.$$

Thus, MICBSO has a positive probability of reaching the ε-optimal population state set. Lemma A2 is proved. □

**Theorem** **A2.**
*The best fitness sequence of MICBSO is convergent.*

**Proof.** According to Definition A3, the historical best solution of MICBSO is preserved during the iteration process. Therefore, the best fitness value satisfies

(A16) $$f(X_{best}(t+1))\leq f(X_{best}(t)).$$

This means that $\left\{f(X_{best}(t))\right\}$ is a monotonically non-increasing sequence. Since $f^{*}$ is the global optimal fitness value, the sequence is bounded below by $f^{*}$, namely

(A17) $$f(X_{best}(t))\geq f^{*}.$$

A monotonic sequence with a lower bound must converge. Therefore, the best fitness sequence $\left\{f(X_{best}(t))\right\}$ generated by MICBSO is convergent. Theorem A2 is proved. □

**Theorem** **A3.**
*MICBSO has global convergence.*

As the number of iterations tends to infinity, the population state sequence generated by MICBSO converges to the optimal population state set $H_{\varepsilon }$ with probability 1. Equivalently, for any ε>0,

(A18) $$\underset{t\to \infty }{lim}\;P\left(f(X_{best}(t))\leq f^{*}+\varepsilon \right)=1.$$

**Proof.** According to Lemma A2, if the current population state does not belong to $H_{\varepsilon }$, MICBSO has a positive probability $q_{\varepsilon }$ of entering $H_{\varepsilon }$ after a finite number of iterations. Therefore, the probability that the population state does not enter $H_{\varepsilon }$ after t iterations satisfies

(A19) $$P(S(t)\notin H_{\varepsilon })\leq (1-q_{\varepsilon }{)}^{t}.$$

Since $0<q_{\varepsilon }<1$, we have

(A20) $$\underset{t\to \infty }{lim}\;(1-q_{\varepsilon }{)}^{t}=0.$$

Thus,

(A21) $$\underset{t\to \infty }{lim}\;P(S(t)\notin H_{\varepsilon })=0.$$

Consequently,

(A22) $$\underset{t\to \infty }{lim}\;P(S(t)\in H_{\varepsilon })=1.$$

According to Definition A5, $S(t)\in H_{\varepsilon }$ means that the best solution stored by MICBSO satisfies

(A23) $$f(X_{best}(t))\leq f^{*}+\varepsilon .$$

Therefore,

(A24) $$\underset{t\to \infty }{lim}\;P\left(f(X_{best}(t))\leq f^{*}+\varepsilon \right)=1.$$

Since ε>0 is arbitrary, the best solution obtained by MICBSO converges to the global optimum in probability. Thus, MICBSO has global convergence. Theorem A3 is proved. □

In summary, the convergence analysis shows that MICBSO satisfies the Markov property, and its best fitness value is monotonically non-increasing due to the best solution preservation mechanism. Moreover, the stochastic search operators, including chaos–opposition-based initialization, Gaussian perturbation, and random boundary reinitialization, provide a positive probability for the algorithm to reach the neighborhood of the global optimum. Therefore, MICBSO can converge to the global optimal solution with probability 1 under the finite-precision and elitist-preservation assumptions.
